# Expression of *Trichoderma* spp. *endochitinase* gene improves red rot disease resistance in transgenic sugarcane

**DOI:** 10.1371/journal.pone.0310306

**Published:** 2024-09-16

**Authors:** Amandeep Sahil Sharma, Anu Kalia, Anuradha Sharma, Mehar Singh Sidhu, Gulzar Singh Sanghera, Gautam Chhabra, Manveer Sharma, Manjinder Singh, Ekta Patel, Piyali Das, Somak Hazra, Ajinder Kaur, Deepak Singla, Jagdeep Singh Sandhu

**Affiliations:** 1 School of Agricultural Biotechnology, Punjab Agricultural University, Ludhiana, India; 2 Electron Microscopy and Nanoscience Laboratory, Punjab Agricultural University, Ludhiana, India; 3 Punjab Agricultural University, Regional Research Station, Kapurthala, India; ICAR - Indian Institute of Wheat and Barley Research, INDIA

## Abstract

Sugarcane (*Saccharum* spp.)is an economically useful crop grown globally for sugar, ethanol and biofuel production. The crop is vulnerable to fungus *Colletotrichum falcatum* known to cause red rot disease. The pathogen hydrolyses stalk parenchyma cells where sucrose is accumulated resulting in upto 75% losses in sugar recovery. In this study, transgenic sugarcane having resistance against red rot was developed by introducing *Trichoderma* spp. *endochitinase* following *Agrobacterium* mediated transformation. The transgene introduction and expression in genetically modified plants were verified through qRT-PCR revealing upto 6-fold enhancement in *endochitinase* expression than non-transgenic plants. Hyperspectral Imaging of transgenic plants displayed altered leaf reflectance spectra and vegetative indices that were positively correlated with ransgene expression. The bioassay with virulent pathotypes of *C*. *falcatum*CF08 and CF13 known for epiphytotic occurrence resulted in identification of resistant plant Chit 3-13.The plants with higher reflectance also displayed improved disease resistance, implying their early classification into resistant/susceptible. The losses in sucrose content were minimized (up to 4-fold) in inoculated resistant plant Chit 3–13 as compared to susceptible non-transgenic plant, and a fewer pathogen hyphae were detected in vascular cells of the former through optical microscopy. The electron micrographs confirmed sucrose-filled stalk parenchyma cells in Chit 3–13; in contrast, cells of non-transgenic inoculated plant were depleted of sucrose. The active sites involved in cleaving 1–4 β-glycoside bonds of N-acetyl-d-glucosaminein the pathogen hyphal walls were detected through endochitinase protein structural modelling. The transgenic sugarcane is an important source for in trogressingred rot resistance in plant breeding programs.

## Introduction

Sugarcane (*Saccharum* spp.) is an economically important crop grown world-wide primarily for producing raw sugar, alcohol and biofuel. The improvement in crop production and productivity is essential to meet demands of sugar industry. The crop is at risk from several pathogens and the fungal pathogen *Colletotrichum falcatum* Went causing red rot is responsible for up to 75% losses in sugar recovery [1,2]. The pathogen mainly spreads through spores in soil, diseased debris, infected cane setts, and secondary transmission occurs by irrigation and rain water [3]. The infection initiates from nodes, leaf scars, root primordia, buds, rootlets, growth cracks and cut edges of the setts [4]. The fungus forms characteristic intracellular, extracellular hyphae and infection pegs in cane stalks that result in drying and discolouration of the stalks due to necrosis of xylem and phloem tissues in vascular bundles [5,6].

Physical control, cultural techniques, resistance breeding, chemical control and biological control are the options available for preventing red rot. Physical and cultural measures are ineffective as no sugarcane cultivar is totally resistant to the pathogen infection attributable to monoculture and emergence of virulent pahto types [7]. The management of the fungus by pesticides is not economically viable and has deleterious effect on crop and environment [8,9]. However, the bio-control agents e.g. *Trichoderma* spp. have been shown to protect against red rot of sugarcane and abiotic stresses [10,11]. *Trichoderma* hydrolyzes the pathogen cell walls consisting of chitin, glucan polymers and proteins [12,13] by secreting enzymes including chitinases that target 1–4β-glycoside bond of N-acetyl-d-glucosamine in chitinand glucan polymers [14,15]. The antifungal mechanism of endochitinase from *Trichoderma* spp. is shown to restrict fungal pathogen growth and provide disease resistance [11]. The cloning, characterization and expression of *endochitinase* genes from *Trichoderma* spp., such as *chit42*,*ech*-*42*,*ThEn-42*,*cht42*, *chit33*, *chit36*,*ech36* in transgenic plants demonstrated their inhibitory action against phytopathogenic fungi [16]. Lorito et al.[17] were the first to develop transgenic tobacco and potato plants by expressing *chit42*that displayed resistance against *Alternaria* spp., *Botrytis cinerea*, *Rhizoctonia solani* and *Sclerotinia sclerotiorum*. However, there is no information about development of transgenic sugarcane carrying *endochitinase* from *Trichoderma* spp. for resistance against *C*. *falcatum*.

This study was carried out to genetically transform sugarcane (that is vegetatively propagated in the region)by expressing *endochitinase* for resistance against red rot. The leaf roll segments were agro-infected for transfer of gene construct carryingCaMV35S-driven*endochitinase*.The transgene was cloned from *Trichoderma* spp. that displayed considerable identity with other *endochitinase* genes in the GenBank database. The introduction of transgene was verified by PCR; qualitative and quantitative transgene expression were determined using RT-PCR and qRT-PCR, respectively, and resistance to red rot was confirmed through bioassay with two virulent *C*. *falcatum* pathotypes. The hyperspectral imaging pointed that the plants with higher *endochitinase* expression had more chlorophyll content, and positive covariance with vegetative indices. Further, the optical microscopy and fluorescence imaging displayed fewer pathogen hyphae in cells of inoculated transgenic plants as compared to non-transgenic plant, and the electron microscopy confirmed presence of sucrose in stalk parenchyma cells of transgenic plant. The losses in sucrose content were minimized (up to 4-fold) in inoculated resistant plant Chit 3–13 as compared to susceptible non-transgenic plant. The structural model of endochitinase protein revealed active sites involved in cleaving 1–4 β-glycoside bond of chitin in pathogen cell wall.

## Methods

### Plant material

The apical tips of Co 0238 cultivar grown and maintained at Regional Research Station, Kapurthala, Punjab, India during autumn season of 2019 were used to prepare explants for culture initiation. The spindle leaf rolls (10 cm in size) excised from the tips were surface sterilized, cut into segments and used for induction of direct shoot regeneration [18].

### Gene construct and its transformation into *Agrobacterium*

The gene construct comprising of *endochitinase* coding sequence (Accession No. KC708870) driven by CaMV35S promoter in pRI 101-ON vector was used to genetically transform leaf roll segments. The construct was moved to EHA105 strain of *Agrobacterium* following the procedure of Holsters et al. [19]. The bacterial culture was smeared onto Luria Bertani (LB) medium (tryptone 10 g/l, yeast extract 5 g/l, NaCl 10 g/l, agar 15 g/l) containing kanamycin (50 mg/l) and placed at 28°C for 48 h. Isolated colonies were picked, verified through PCR and grown in liquid LB medium at 28°C until the cultures reached optical density (OD_600 nn_) of 0.8–1.0. The suspensions were centrifuged at 10,000 rpm for 5 mins and *Agrobacterium* pellets were resuspended in MS medium (50 ml at pH 5.8) augmented with acetosyringone (9.8 mg/l) following the method of Manickavasagam et al. [20].

### Plant transformation and direct shoot regeneration

The sterile leaf segments were cultured for 4–6 days on MS medium containing Kin (0.5 mg/l), NAA (5 mg/l) and agar (8 g/l), proceeded by shaking (manually) for 15 mins in *Agrobacterium* suspension supplemented with acetosyringone according to Sandhu et al. [18]. The agro-infected segments were dried on sterile filter paper (Whatman No. 1), followed by co-cultivation for 36–48 h on basal MS medium supplemented with acetosyringone (9.8 mg/l). Subsequently, the segments were washed three times with autoclaved water carrying cefotaxime (500 mg/l), dried and moved for shoot regeneration on MS medium augmented with Kin (0.5 mg/l), NAA (5 mg/l), agar (8 g/l) and cefotaxime (500 mg/l) for three weeks. The cultures were kept at 25 ± 2°C with photoperiod of 16 h [18]. The shoots induced on leaf segments were detached aseptically and transferred to a fresh regeneration medium for shoot elongation and induction of roots. The putative transformed plantlets so obtained were placed on moistened cotton in plastic jars and covered with polythene sheet for hardening by incubating at room temperature for three days and then in a transgenic glasshouse for another three days. The plantlets were then transferred to soil mixed with organic manure (3:1 w/w) in earthen pots and maintained under a relative humidity of 80% at 30°C. These were watered at two-day interval and supplemented weekly with liquid MS containing half strength salts.

### PCR analysis

The young leaves of two month-old transformed plants were collected to isolate genomic DNA by CTAB method [21]. PCR was carried out with *endochitinase* gene specific forward (5ʹ- CGCGGATCCTTGGGCTTCCTCGGAAAA-3ʹ) and reverse (5ʹ- CGGGGCCCCTAGTTGAGACCGCTT-3ʹ) primerswith added *BamH*I and *Apa*I restriction sites at 5′ and 3′ ends, respectively. PCR mixture (10 μl) contained 30 ng DNA (1 μl), 10 μM of each primer (0.5 μl), master mix (5 μl) containing dNTPs, MgCl_2_, PCR buffer, *Taq* polymerase (Promega, USA) and water (3 μl). The mixtures were placed in a thermocycler (Eppendorf, Germany) using specific reaction profile set for preliminary denaturation at 95°C for 5 mins, and then 35 cycles of denaturation at 95°C for 1 min, annealing at 63°C for 2 mins, extension at 72°C for 1 min with a concluding extension at 72°C for 7 mins. PCR amplicons were resolved electrophoretically on ethidium bromide-stained 1% agarose gel run at 90 V for 1–2 h, and captured with photo gel documentation system (Analytic Jena, Germany).

### RT-PCR analysis

The leaf tissues of two and a half month-old sugarcane plants were collected to isolate RNA using FastRNA PRO^TM^ GREEN KIT (MP Biomedicals, India) in liquid nitrogen. The nucleic acid was subjected to DNase I treatment for cleanup of the DNA degradation. The RNA integrity was established using 1.2% denaturing gel made in 1X MOPS buffer (EDTA 10 mM, C_2_H_3_NaO_2_ 80 mM, MOPS 200 mM) and run at 90 V for 1–2 h. The first strand cDNA synthesis was carried out with 2 μg of RNA using TaKaRa Primescript^TM^ kit following instructions given in the kit. cDNA legitimacy was established with *26S rRNA* specific forward (5ʹ-CACAATGATAGGAGGAGCCGAC-3ʹ) and reverse (5ʹ- CAAGGGAACGGGCTTGGCAGAATC-3ʹ) primers [22] using a profile described earlier for PCR. The products were electrophoresed on ethidium bromide-stained 2% denaturing agarose gel at 90 V for 1 h. The authenticated cDNA was reverse transcribed using *endochitinase* specific primers for determining expression of the transgene.

### qRT-PCR analysis

The quantitative expression of *endochitinase* in the transformed plants was analysed using qRT-PCR, with *tubulin* as a reference gene. The *endochitinase* gene specific forward (5ʹ- GTATGTTCTGGGAGGCTTCTG-3ʹ), reverse (5ʹ- ATGTTATCATACTGGGAGTTGGG-3ʹ), and *tubulin* gene specific forward (5ʹ- CCAAGTTCTGGGAGGTGATCTG-3ʹ) and reverse (5ʹ-TTGTAGTAGACGTTGATGCGCTC-3ʹ) primers were used for relative expression analysis [23]. The analysis was done using SteponePlus^TM^ Realtime PCR machine (ABI, India). The reaction mixture of 20 μl contained 2 μl of cDNA, 1 μl of each primer, 10 μl of SYBR Premix Ex Taq™ II (Takara, Japan),and 6 μl of sterile water. The reactions for both *tubulin*(house keeping gene used for expression normalization) and *endochitinase* were run-on profile: 94°C for 3 min, 35 cycles for 10 sec at 94°C, 42 sec at 62°C, 30 sec at 72°C and melt curve at55°Cfor 30 sec. The ten-fold cDNA dilutions were used to prepare a standard curve for each assay, and the experiment was performed in triplicate. The C_T_ values obtained for *endochitinase* and *tubulin* were used to analyse expression, and ΔΔC_T_ method was used to determine relative expression of the transgene according to Livak & Schmittgen [24].

### Hyperspectral imaging

The four-month-old transgenic sugarcane plants were phenotyped according to reflectance spectra and vegetative indices through HSI. The third leaf from top was excised from each transgenic and non-transgenic plant. The leaf samples were kept under wet conditions to avoid moisture loss and change in chlorophyll content. The images were captured from central portion of each leaf (15 cm in length) placed at a distance of 30 cm from HSI system (Specim, Spectral Imaging Ltd., Finland) under natural sunlight (~1200–1500 h) within 30 min of excision. The data on reflectance spectra was extracted from ~ 4 x 4 cm leaf region using Specim studio software. The experiment was performed on three leaves of each plant and the standard reflectance spectrum was obtained using a white reference plate. The changes in light intensity were nullified by considering the background score for every sample.

The vegetative indices, such as Green Chlorophyll Index (GCI = λ_NIR_/λ_Green_− 1) and Green Normalized Difference Vegetation Index (GNDVI = λ_NIR_ + λ_Green_/λ_NIR_ + λ_Green_) were calculated by averaging the reflectance data over ± 20 nm wavelength intervals [25]. Pearson’s correlation coefficient was estimated between vegetative indices and fold-change in transgene expression (https://www.socscistatistics.com/tests/pearson/default2.aspx).

### Bioassay for red rot incidence

*C*. *falcatum* pathotypes i.e. CF08 and CF13 were used to assess red rot incidence on 10-month-old transgenic plants. The culture of CF08 (National Agriculturally Important Microbial Culture CollectionNo. F-03384) was collected from Indian Institute of Sugarcane Research, Lucknow during 2021-22and maintained on CoJ 64, its host differential. The pathotype CF13 was isolated and maintained on its differential Co 0238 following the technical procedure of Plant Pathology All India Coordinated Research Project on Sugarcane, New Delhi. The pathotype cultures were raised individually and conidial suspensions (70,000 conidia/ml) were used to plug inoculate main stalks of transgenic as well as non-transgenic plants following procedure outlined by Nayyar et al. [26]. The disease development in transgenic glasshouse was ensured by maintaining high humidity (>80%) and symptoms were evaluated in the stalks split longitudinally eight weeks after inoculation. The incidence of disease development was recorded based on disease lesions spread vertically on the stalk, lesion width, condition on top, nodal transgression and display of white spots. The experiment was performed in triplicate. The symptoms were scored on 0–9 scale given by Srinivasan and Bhat [27].

Pearson’s correlation coefficient was calculated for determining relationship between *x* (fold-change in *endochitinase* expression) and *y* (red rot incidence) with the formula:

$$r=\frac{\sum (x_{i}-\bar{x})(y_{i}-\bar{y})}{\sqrt{\sum {\left(x_{i}-\bar{x}\right)}^{2}\sum ({y_{i}-\bar{y})}^{2}}}$$

where, *r* = Pearson’s correlation coefficient; *x_i_* = x−variable value in a sample; $\bar{x}$ = x−variable values’ mean; *y_i_* = y−variable value in a sample; $\bar{y}$ = y−variable values’ mean.

### Assessment of sucrose content in cane juice

The sugarcane plants were analyzed for Brix (%) to estimate sucrose content (cane sugar) following the method given by Sugar Research Australia [28]. The drops (2–3) of juice extracted from lower most cane internode were placed on hand held refractometer (Thermocare, India) to determine Brix value. The experiment was performed on three different canes of each plant and the data was analysed for mean ± SD.

### Optical microscopy and fluorescence imaging of stalks

The stalks from *C*. *falcatum* inoculated and non-inoculated 12-month-old sugarcane plants were collected and cut longitudinally to obtain two equal halves for microscopic examination. The transverse sections of the stalk tissue were obtained by free hand sectioning with a surgical blade. The sections included some part of seemingly healthy tissue i.e. not containing red colouration and were placed on Whatman filter paper soaked with acetic acid and alcohol (1:1 v/v) for one day under refrigerated conditions. The sections were exposed to aqueous solution of lacto-glycerol (1:1:1 v/v) briefly, followed by staining with buffered aniline blue dye (0.1% w/v) that non-specifically stained chitin and cellulose components of fungal and plant cell walls, respectively [29]. These were then placed on glass slide in glycerol and water solution (30% v/v). The slides (ten sections per treatment) were viewed under epifluorescence microscope (Leica DM-5000, Germany) equipped with UV-source and CCD detector system.

### Scanning electron microscopy

The sugarcane stalks collected from 12-month-old plants (inoculated and non-inoculated) were sectioned diagonally about 4 cm above the bore hole to obtain discs of approximately 1 cm thickness. The sub-sectioned discs were pre-fixed, washed, dehydrated and dried according to the method described by Nayyar et al. [26]. The gold layering (10–20 nm thick) of the sections was carried out to make them conductive with ion sputter coater (Hitachi E-1010, Japan). The micrographs were taken using scanning electron microscope (Hitachi S-3400N, Japan).

### Estimation of endophytic microbial population in roots

The viable endophytic microbial cells inhabiting the roots of 12-month-old CF13inoculated (transgenic, non-transgenic) and non-inoculated (non-transgenic) plants were counted following the method given by Bacon and Hinton [30]. The root tissue (100 mg fresh weight) was excised, washed thoroughly under tap water and sterilized with sodium hypochlorite (4% w/v) containing Tween 20 (0.1% v/v) in Falcon tubes (50 ml; Tarsons, India) for 4 min. The roots were rinsed thrice in autoclaved water to remove the sterilant and dab dried on sterile paper towels placed in a petri plate. The surface-sterilized roots were i) macerated to prepare root extract serial dilutions in phosphate buffer (pH 7.1) that were plated on nutrient agar, PDA, Jensen’s agar, King’s B agar and Pikovskaya’s agar in triplicate and incubated at 25±2°C to obtain viable endophytic bacterial and fungal cell counts (10^3^ cfu/ml), and ii) cut into segments (10 mm long), cultured horizontally on nutrient agar, PDA media and incubated for the appearance of microbial colonies.

### Modelling and docking of *endochitinase* gene

The protein sequence of *endochitinase* (ID AGL13263.1) was selected for predicting three dimensional structure using Phyre_2_, and its evaluation through SAVES webserver [31]. The 3D structure of N-acetyl-glucosamine (NAG) [PubChem ID: 24139] was downloaded in SDF format and energy of ligand was minimized using obminimizer program of Open Babel [32]. A site-specific docking of best predicted protein model was carried out by AutoDock4.2 [33]. The ligand and receptor were prepared by addition of hydrogen ions, charged (i.e. gasteiger for ligand and Kollmann for protein), followed by merging of non-polar hydrogen atoms and defining AD4 atom types. A 3D grid box with coordinates (X-60.76Å, Y-31.74Å, Z-44.98Å) and box size (46, 40, 46) was used to cover the active region of protein by AutoGrid. A lamarckian genetic algorithm (LA) with default parameters keeping the receptor as rigid and ligand as flexible was used for docking study. The best docked conformation was selected from the scoring functions and ranked according to their binding efficiencies. Finally, the interactions between ligand and protein were visualized using ligplot software.

### Statistical analysis

The relative expression of *endochitinase* in transgenic plants was calculated by 2^-ΔΔC^_T_ formula from quantitative data (collected from three replications) and evaluated using CFX manager 3.0 software [24]. Pearson’s correlation coefficient was used to estimate the relationship of fold-change in transgene expression with red rot incidence, where P = 0.1 was regarded as significant statistically.

## Results

### Genetic transformation of sugarcane

**Fig 1:**
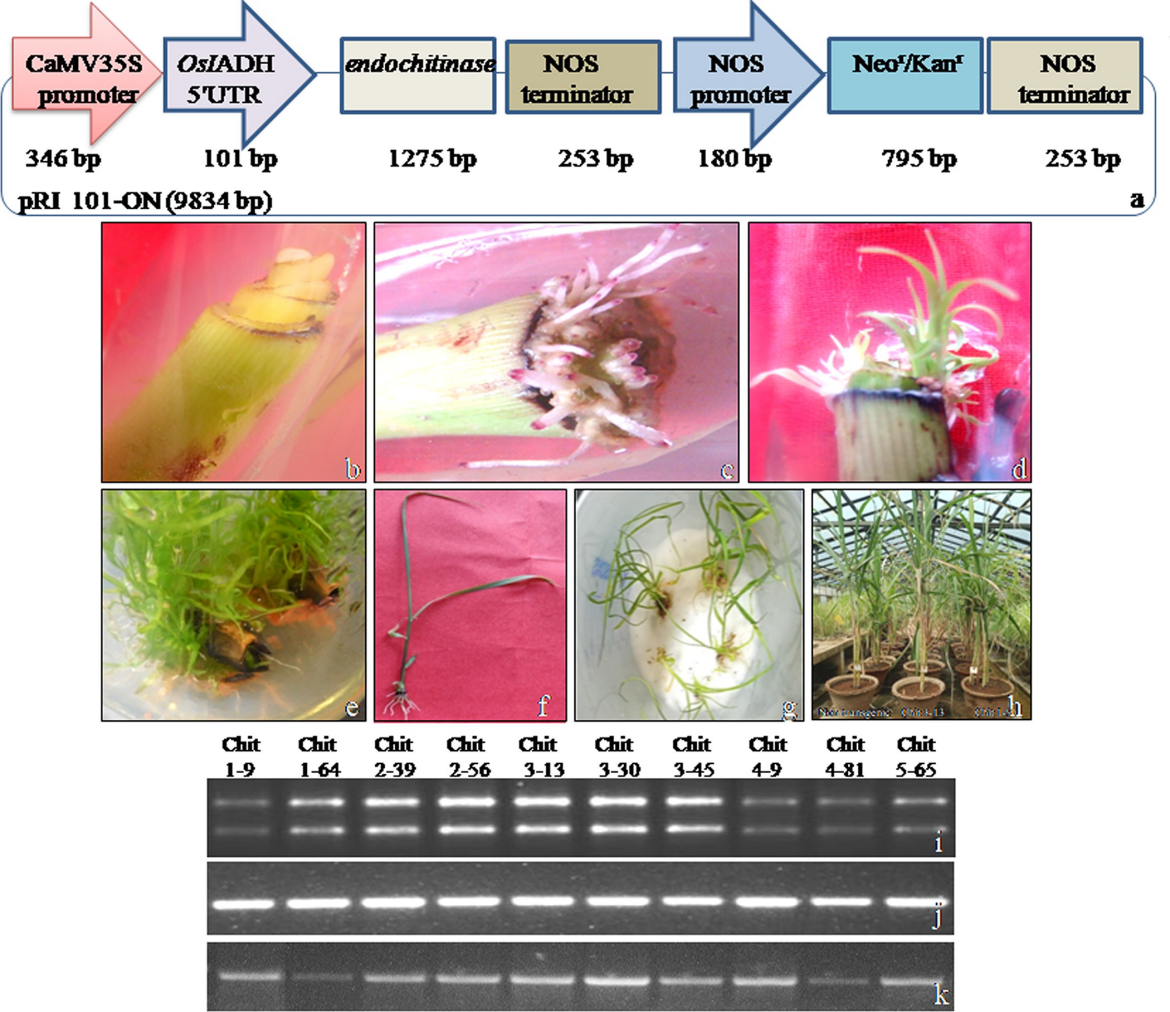
Genetic transformation of sugarcane spindle leaf roll segments following agro-infectionand semi-quantitative reverse transcription-PCRanalysis of putative plants. **(a)** Gene construct showing *endochitinase* driven by CaMV35S promoter.**(b)** Pre-cultured leaf roll segments. **(c)** Direct emergence of roots. **(d)**Direct emergence of shoots. **(e)** Multiple shoot formation. **(f)** Plantlet formation. **(g)** Hardening of plantlets on moist cotton. **(h)** Transfer of plants to soil. **(i)** RNA of PCR positive plants designated as 1–9 to 5–65. **(j)** cDNA amplified by *26S rRNA* gene specific primers. **(k)**cDNA amplified by *endochitinase* gene specific primers.

The leaf segments of Co 0238sugarcane cultivar were agro-infected with EHA105 strain carrying antifungal *endochitinase* gene driven by CaMV35S promoter (Fig 1). A total of 2722 segments were pre-cultured and 1911 were agro-infected. The roots carrying a distinguishable purple coloured marker on tips were induced on agro-infected leaf segments *in vitro*, followed by emergence of shoots directly from the segments without callus interphase (Fig 1). The shoots emerged in bunches from the cut ends of leaf segments at more than one place. The shoot induction was noticed in 738 segments; 534 putative plantlets were transferred to soil after hardening (S1 Table). The putative transformed plants displayed normal growth and development in transgenic glass house (Fig 1).

### Characterization of transformed plants

PCR analysis: The putative plants were analyzed for the introduction of transgene byPCR. The primers used in PCR had no similarity with in he rent sugarcane *endochitinase* genes. The results showed appearance of 1275 bp amplicon in ten plants designated as Chit 1–9, Chit 1–64, Chit 2–39, Chit 2–56, Chit 3–13, Chit 3–30, Chit 3–45, Chit 4–9, Chit 4–81 and Chit 5–65 (S1 Fig). PCR on genomic DNA from 10 putative plants using *AgrobacteriumvirG* region specific primers did not show any amplification (S2 Fig), whereas an amplicon of 359 bp was observed in plasmid DNA of *Agrobacterium* strain LBA4404, substantiating that the putative T_0_ plants were not contaminated by LBA4404 cells, and hence not false positives.

RT-PCRanalysis: The plants displaying introduction of the transgene were analyzed for transcription through RT-PCR. cDNA synthesis was validated using PCR primers specific to*26S rRNA* gene resulting in 534 bp amplicon, demonstrating the validity of the synthesized cDNA (Figs 1 and S3). The results showed differential expression of *endochitinase* in the ten plants.

qRT-PCR analysis: The quantitative expression of *endochitinase* in plants showing transcription of the transgene and non-transgenic control was analyzed through qRT-PCR that was standardized by generating a standard curve using *tubulin* and transgene specific primers (S4 Fig). The stability of the experiment was validated from negligible discrepancy in C_T_ values of *tubulin* and *endochitinase* replicates (S5 and S6 Figs).qRT-PCR analysis revealed that seven plants designated as Chit 1–64, Chit 2–39, Chit 2–56, Chit 3–13, Chit 4–9, Chit 4–81, Chit 5–65 had 3–6 fold more relative expression from non-transgenic control (Table 1; S2 and S3 Tables; S7 Fig). The plant designated as Chit 3–30 had an expression of 0.93 and was lower than relative expression of non-transgenic control, while plants designated as Chit 1–9 and Chit 3–45 had a relative expression of 1.83 and 1.12, respectively. The results indicated up-regulation of *endochitinase* expression in seven plants and stable integration of transgene in genome of transformed plants.

**Table 1:** Bioassay on transgenic sugarcane plants for red rot incidence.

Plant designation	Relative *endochitinase* expression (2^- ΔΔC^_T_)	Pathotype				
		CF08			CF13	
		Scale (0–9)*	Reaction		Scale (0–9)*	Reaction
NTC	1 (0.90–1.01)	8.0	S		9.0	HS
Chit1-9	1.83 (1.80–1.87)	4.2	MS		7.6	S
Chit 1–64	3.60 (3.41–3.81)	4.1	MS		8.6	HS
Chit 2–39	4.82 (4.79–4.86)	3.6	MR		8.3	HS
Chit 2–56	3.68 (3.63–3.73)	4.4	MS		7.7	S
Chit 3–13	6.87 (6.82–6.92)	2.0	R		2.0	R
Chit 3–30	0.93 (0.89–0.96)	6.3	S		8.0	S
Chit 3–45	1.12 (1.08–1.16)	4.7	MS		8.3	HS
Chit 4–9	5.39 (5.24–5.54)	2.9	MR		8.6	HS
Chit 4–81	4.99 (4.89–5.09)	3.3	MR		8.0	S
Chit 5–65	4.29 (4.14–4.44)	2.9	MR		8.3	HS

Values are means of three replicates and the numbers in parentheses represent range of change in *endochitinase* expression as determined by 2^-ΔΔC^_T_ method. NTC denotes non-transgenic control plant.

*Refers to red rot rating scale developed by Srinivasan and Bhat (1961), where R = Resistant (0–2), MR = Moderately Resistant (2.1–4), MS = Moderately Susceptible (4.1–6), S = Susceptible (6.1–8), HS = Highly Susceptible (>8).

### Hyperspectral imaging of transgenic plants for vegetative indices

**Fig 2:**
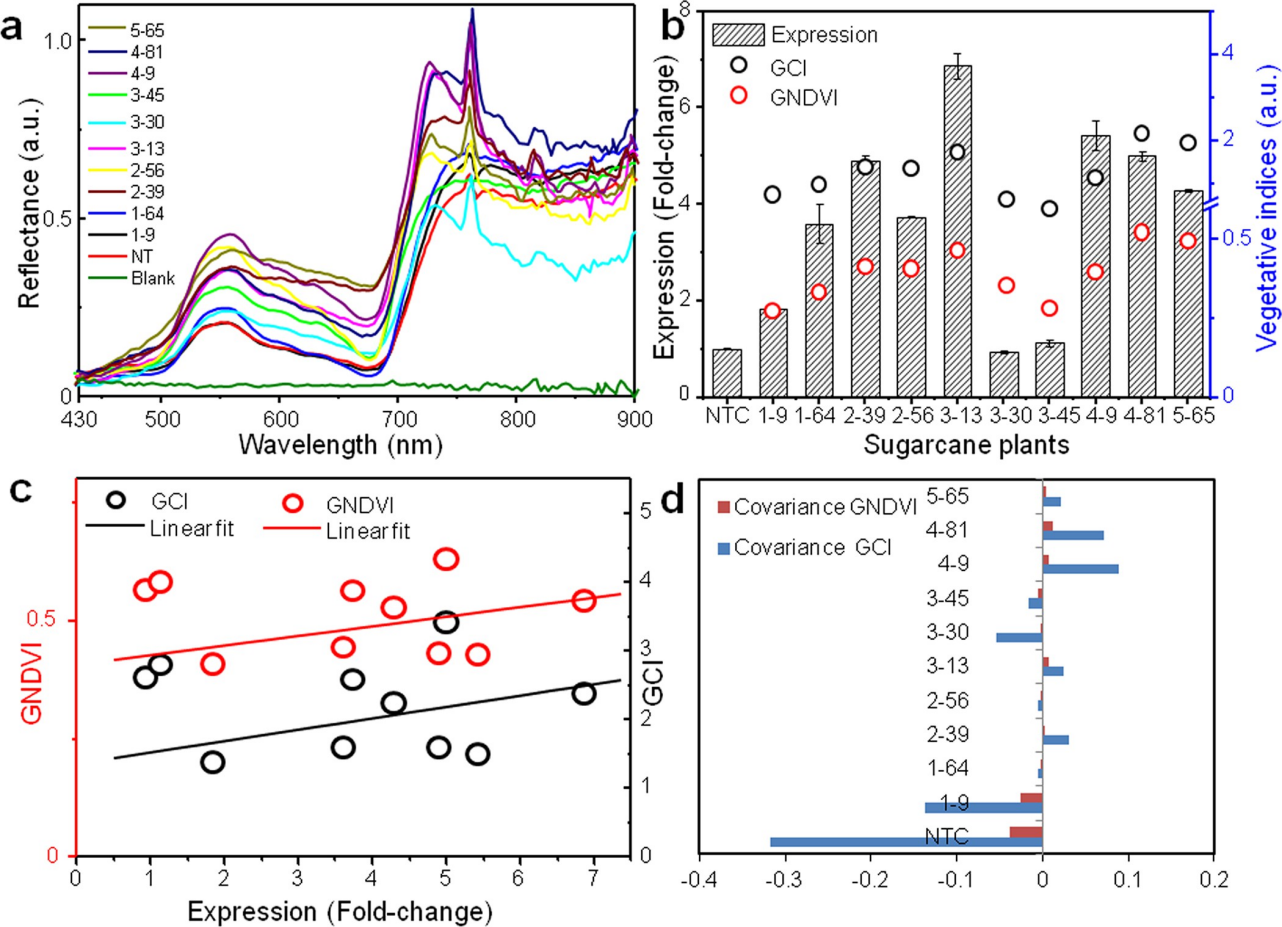
Hyperspectral imaging of transgenic sugarcane leaves. **(a)** Leaf reflectance spectra. **(b)** Double Y-plot of vegetative indices as a function of fold-change in *endochitinase* expression. The bars represent mean standard error.**(c)** Relationship between change in transgene expression and vegetative indices as determined by Pearson’s correlation coefficient. **(d)** Covariance plot of transgenic sugarcane plants against vegetative indices.

The leaves of transgenic sugarcane plants were examined through Hyperspectral Imaging (HSI) for distinguishing their vegetative indices on the basis of reflectance spectra (Fig 2A). Chit 2–39, 3–13, 4–9, 4–81, 5–65 displayed higher reflectance in the near-IR range (700 to 900 nm) in comparison to Chit 1–9, 1–64, 2–56, 3–30, 3–45 and non-transgenic plants that exhibited low reflectance (S8 Fig; S4 Table). The transgene expression and vegetative indices were associated, where GCI varied from 1.5 to 2.5 and GNDVI ranged from 0.25 to 0.5 (Fig 2B and 2C). Pearson’s correlation coefficient confirmed the association of GCI and GNDVI with transgene expression (R-value of 0.58 and 0.69, respectively) [S4 Table], suggesting that the plants with higher expression had more chlorophyll content. Further, the plants displaying higher reflectance demonstrated positive covariance of GCI and GNDVI with transgene expression, whereas those exhibiting low reflectance had negative covariance (Fig 2D). The analysis also indicated that relationship between expression and GNDVI was more potent than with GCI.

### Bioassay for red rot incidence

**Fig 3:**
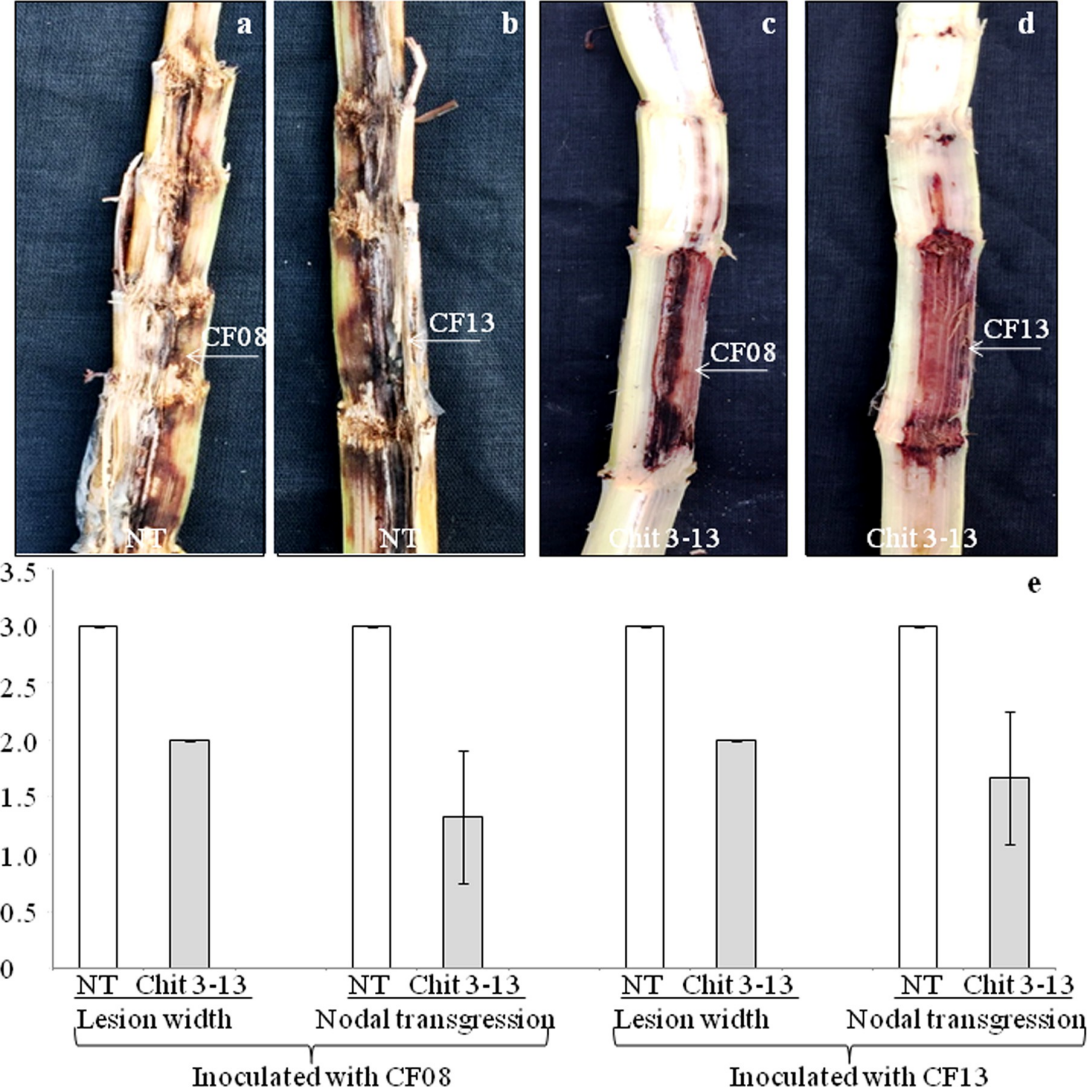
Bioassay for red rot incidence. **(a)**Non-transgenic plant showing susceptibility after inoculation with CF08. **(b)** Non-transgenic plant inoculated with CF13 revealing highly susceptible. **(c)** Transgenic plant Chit 3–13 inoculated with CF08 displaying resistance. **(d)** Transgenic plant Chit 3–13 inoculated with CF13 showing resistance. Arrows indicates point of inoculation. **(e)** Graphical representation of disease symptoms. The bars represent mean standard error.

The plants were assessed for disease incidence with virulent pathotypes i.e., CF08 and CF13 of *C*. *falcatum* known to cause red rot. The non-transgenic plants inoculated independently with either of the two pathotypes displayed susceptible reaction after eight weeks. The symptoms included occurrence of meagre white spots along the length of split stalk, transgression of disease lesions covering four or more nodes, spread of lesions to entire width of the stalk (Fig 3A and 3B) with drying of top leaves. In contrast, transgenic plant Chit 3–13 inoculated separately with CF08 and CF13 displayed no white spots and no lesion transgression with minimal discolouration(Fig 3C and 3D), 50% spread of the lesion in stalk along with green leaves on top, indicating resistance against the pathotypes (Table 1; Fig 3E;S5 Table). The plants Chit 1–9, Chit 1–64, Chit 2–39, Chit 2–56, Chit 3–30, Chit 3–45, Chit 4–9, Chit 4–81 and Chit 5–65 revealed moderately resistant to moderately susceptible reaction to CF08, and susceptible to highly susceptible reaction to CF13 (Table 1). The experiment pointed towards differential reaction of transgenic plants to CF13 and CF08 with former showing more virulence as compared to the latter. Further, the plants expressing *endochitinase*at high level had improved defence potential against virulent *C*. *falcatum* pathotypes and exhibitedreduced disease incidence having Pearson coefficient value of—0.82 for CF08,and—0.54 for CF13 (both significant at *P* = 0.1) [S6 Table].

### Assessment of sugarcane juice for sucrose content

**Fig 4:**
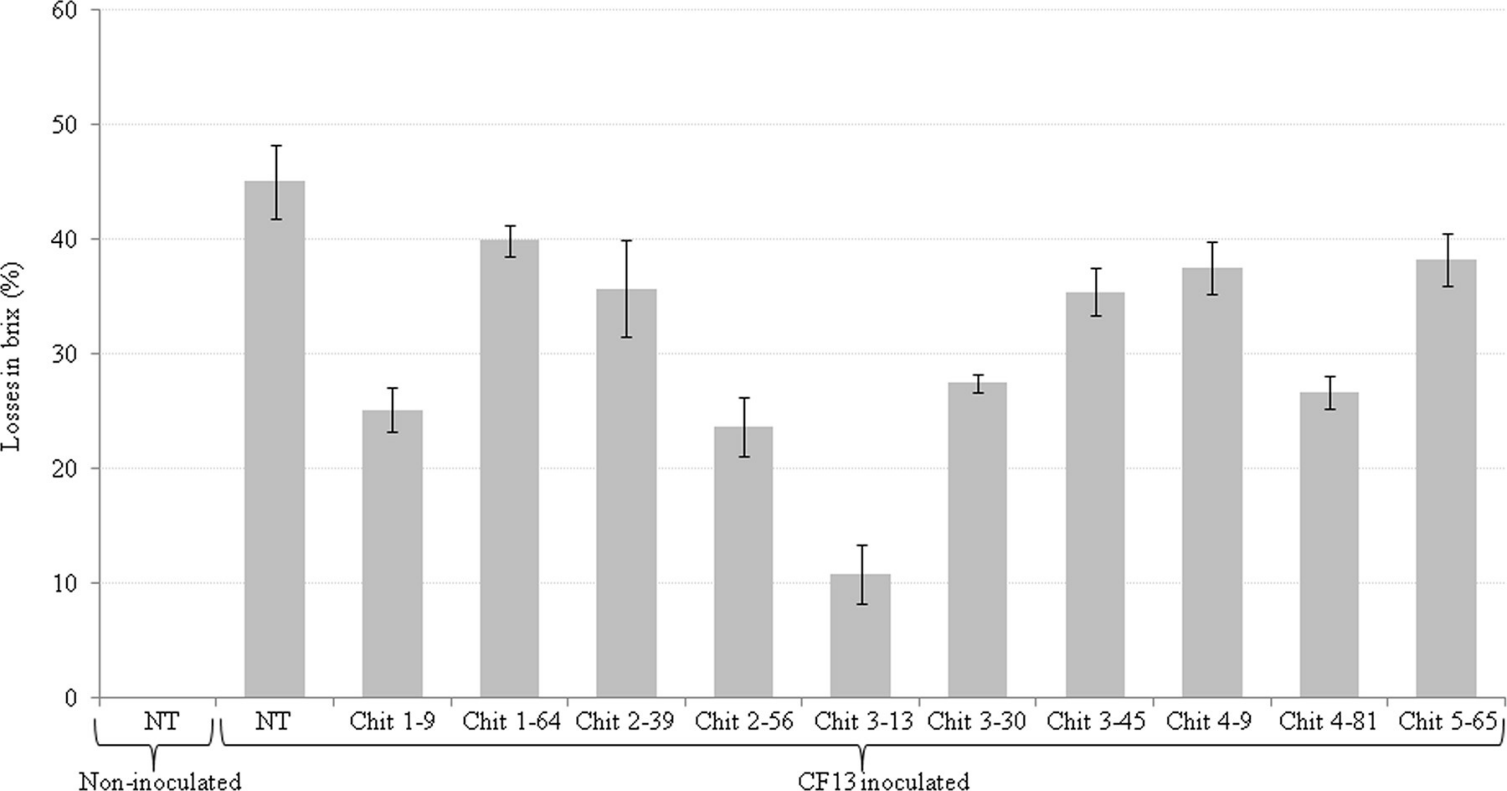
Sucrose content losses in transgenic plants inoculated with CF13.The bars represent mean standard error.

The cane juice of non-transgenic non-inoculated, transgenic and non-transgenic plants inoculated with virulent CF13 pathotype was analyzed for Brix (sucrose content). The Brix value of inoculated transgenic plants ranged from 12.03±0.35 to 17.86±0.30%that was significantly higher than the inoculated non-transgenic plant (11.0±0.50%) [S7 Table]. The losses in Brix were minimized(10.79±2.62%) in inoculated resistant transgenic plant Chit 3–13 as compared to higher losses (45.06±3.17%) in non-transgenic inoculated plant [Fig 4; S7 Table].The experiment demon started that transgenic plant displaying resistance had considerably higher sucrose content(4-fold)indicating the role of *endochitinase* transgene in curtailing sucrose losses despite prevalence of the virulent pathogen.

### Optical microscopy and fluorescence imaging of stalks for *C*. *falcatum* pathogenesis

**Fig 5:**
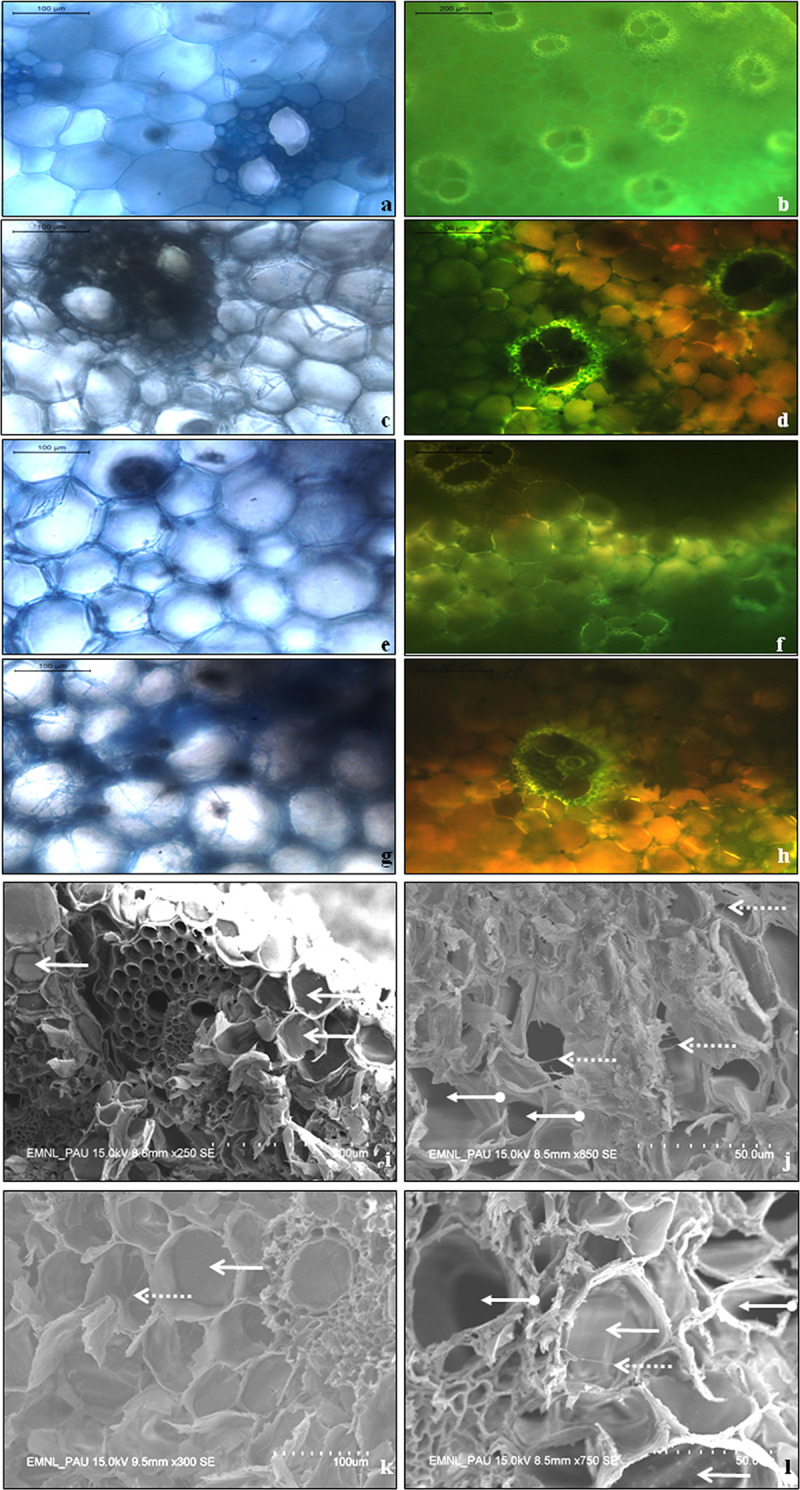
Microscopy of sugarcane stalk tissue and parenchyma cells. Optical microscopy and fluorescence imaging of sections from stalk tissue of non-inoculated non-transgenic sugarcane exhibiting **(a)** clear middle lamella **(b)** normal vascular bundles; Inoculated non-transgenic tissues displaying **(c)** fungal hyphae ramifying through the middle lamella **(d)** inflated vascular tissues showing loss of green autofluorescence; The transgenic plant Chit 3–13 inoculated with CF08 exhibiting **(e)** fewer hyphae in the middle lamella and inside the cells **(f)** normal green autofluorescence; The transgenic plant Chit 3–13 inoculated with CF13 depicting **(g)** fungal hyphae present in middle lamella as well as inside the cells **(h)** loss of normal autofluorescence. Scanning electron microscopy of sugarcane stalk parenchyma cells**(i)** The cells of non-transgenic non-inoculated plant, bold arrows indicate the characteristic turgid sucrose-filled cells**(j)** The cells of non-transgenic plant inoculated with CF08, dotted arrows indicate presence of pathogen hyphae and arrows with circular end show sucrose depleted cells **(k)** The cells of transgenic plant Chit 3–13 inoculated with CF08, bold arrows indicate turgid cells and dotted arrows depict presence of pathogen hyphae **(l)** The cells of Chit 3–13 inoculated with CF13, bold arrows indicated sucrose-filled turgid cells, dotted arrow represents presence of pathogen hyphae and arrow with circular end shows the sucrose depleted cell.

The optical microscopy and fluorescence imaging were carried out on stalk tissue to analyze incidence of the pathogen hyphae. The microscopy of non-inoculated non-transgenic tissues exhibited clear middle lamella (Fig 5A), and imaging depicted normal vascular bundles (Fig 5B). In contrast, the inoculated non-transgenic tissues displayed inflated vascular tissues with fungal hyphae ramifying through middle lamella as well as inside the cells (Fig 5C and 5D). The transgenic plant Chit 3–13 inoculated with CF08 exhibited a fewer hyphae in the middle lamella and inside the cells (Fig 5E) with normal green autofluorescence (Fig 5F). The inoculation of Chit 3–13 with CF13 depicted a few fungal hyphae present in the middle lamella as well as inside the cells (Fig 5G). The damage caused by the pathotype was identified as loss of green autofluorescence signal from sugarcane stalk cells (Fig 5H). The results confirmed that i) transgenic sugarcane plant cells restrained pathogen hyphal growth, and ii) CF13 pathotype was more virulent than CF08.

### Scanning electron microscopy of parenchymatous cells for occurrence of pathogen hyphae

The diagonal sections of sugarcane stalk were analyzed through scanning electron microscope for occurrence of pathogen hyphae in parenchyma cells where sucrose is accumulated. The micrographs of non-transgenic non-inoculated stalk sections revealed typical turgid parenchymatous cells filled with sucrose (Fig 5I). In comparison, the stalk sections of non-transgenic plant inoculated with CF08 displayed the presence of pathogen hyphae and parenchyma cells depleted of sucrose (Fig 5J). The sections of transgenic plant Chit 3–13 inoculated with CF08 exhibited turgid sucrose-filled parenchyma cells with trailing hyphae (Fig 5K). On the contrary, the sections of Chit 3–13 inoculated with CF13 revealed parenchyma cells filled with sucrose along with presence of pathogen hyphae and also the occurrence of a few sucrose-depleted cells (Fig 5L). The results confirmed that the transgenic plant Chit 3–13 restrained pathogen hyphae from depleting the parenchymatous cells of sucrose.

### Effect of *endochitinase* expression on endophytic microbial cell count in roots

The root tissues of non-transgenic (non-inoculated, inoculated) plants, and inoculated resistant transgenic plant Chit 3-13were assessed for ascertaining cell count of endophytic microbes. The non-transgenic non-inoculated plant demonstrated high cell count of total bacteria, nitrogen fixers and significant presence of pseudomonads, fungi and P-solubilizers (Table 2). On the contrary, the non-transgenic inoculated plant displayed maximum count of nitrogen fixers, followed by presence P-solubilizers, total bacteria, fungi and pseudomonads (Table 2). The transgenic plant Chit 3–13 displayed moderate number of total bacteria, followed by nitrogen fixers and P-solubilizers with no incidence of pseudomonads and total fungi (Table 2). The results pointed towards decline in viable cell counts of endophytic bacteria and nitrogen fixers colonizing roots of resistant transgenic plant Chit 3–13. Further, the culture of root segments from resistant plant showed complete absence of pseudomonads and total fungi (S9 Fig). The results demonstrated that high *endochitinase* expression in resistant plant was directly correlated with low viable endophytic microbial cell count.

**Table 2:** Endophytic microbial cell count in roots of transgenic sugarcane plants inoculated with CF13 pathotype.

Endophyte	Plant designation	Cell count (Mean ± SE)
**Total bacteria**	Non-transgenic (non-inoculated)	21.33±2.51
	Non-transgenic (inoculated)	2.06±0.25
	Chit 3–13	11.0±2.00
**P-solubilizers**	Non-transgenic (non-inoculated)	0.16±0.05
	Non-transgenic (inoculated)	2.4±0.43
	Chit 3–13	1.2±0.36
**Nitrogen fixers**	Non-transgenic (non-inoculated)	18.33±2.51
	Non-transgenic (inoculated)	15.66±1.52
	Chit 3–13	1.33±0.57
**Pseudomonads**	Non-transgenic (non-inoculated)	0.86±0.15
	Non-transgenic (inoculated)	0.06±0.05
	Chit 3–13	0.0±0.00
**Total fungi**	Non-transgenic (non-inoculated)	0.43±0.05
	Non-transgenic (inoculated)	0.56±0.05
	Chit 3–13	0.0±0.00

Note: Microbial cell count is presented as 10^3^cfu/ml; Values of three replicates are shown as mean ± SE.

### Structural modelling of the catalytic domain for detection of active site residues

**Fig 6:**
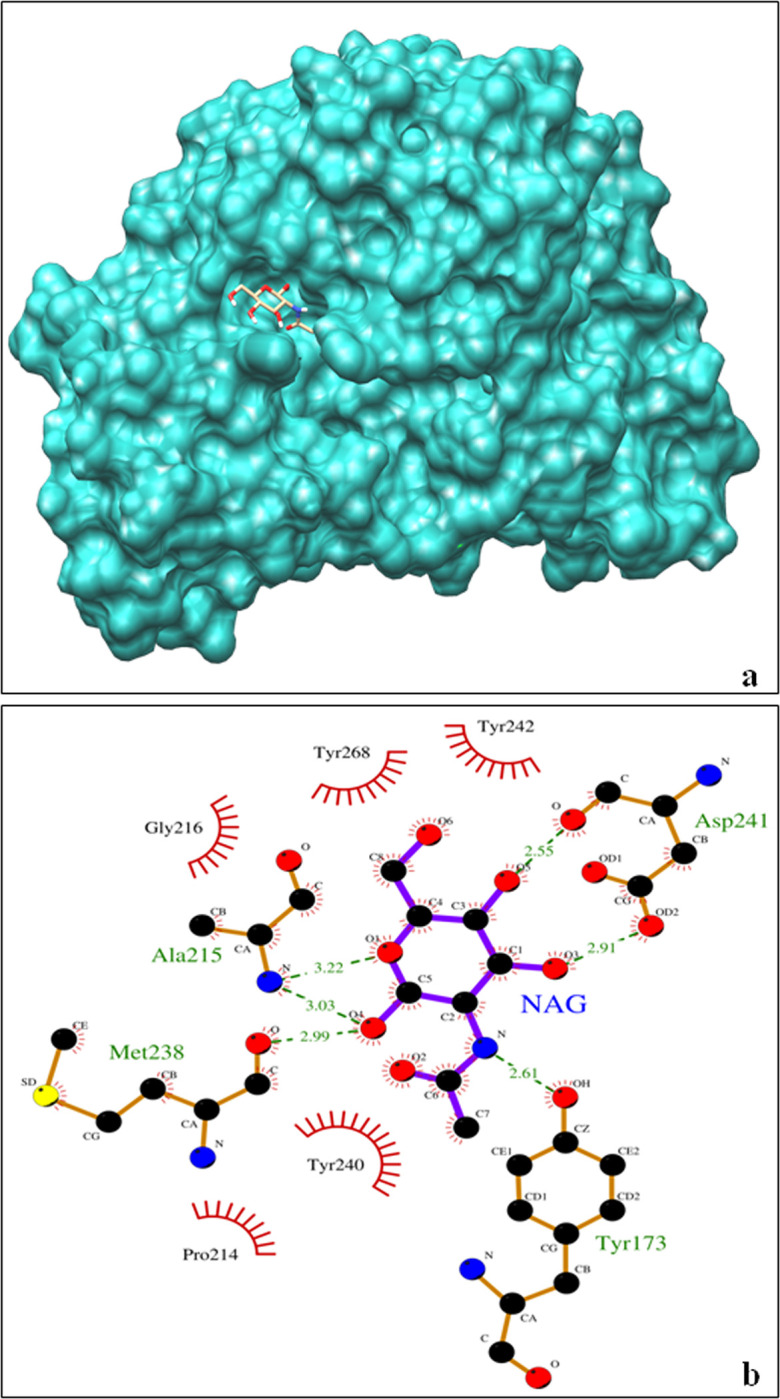
Docked complex of endochitinase with NAG. **(a)** Binding of six H-bonds with NAG. **(b)** Endochitinase protein docked with NAG.

The 3D structure of *endochitinase* was generated using PDB-ID 6EPB template. The template displayed 84% identity with 92% sequence coverage leading to generation of structure for 389 (36–424) residues. The structural analysis revealed that 90.5% residues were present in core region, 9.2% in allowed and 0.3% in generally allowed regions with overall ERRAT score value 86.61 (S10 Fig). No residues were observed in disallowed region suggesting that the model quality was high. The verify3D also demonstrated that more than 99.74% residues had score ≥0.1 (S11 Fig). The docking analysis revealed-4.41 KJ/mol binding free energy and a total of six H-bonds with NAG. Further, the results pointed towards involvement of Tyr173, Ala215, Asp241, Met238 in formation of H-bonds, while Gly216, Tyr268, Tyr242, Tyr240 and Pro214 were present in close vicinity of the ligand molecule (Fig 6A and 6B). The structural model revealed active sites having role in cleaving 1–4 β-glycoside bond of N-acetyl-d-glucosamine.

## Discussion

This study describes expression of non-plant *endochitinase* gene from *Trichoderma* spp.in sugarcane crop that improved resistance against red rot. The disease resistance was incorporated in Co 0238, an early maturing commercial sugarcane cultivar of north-western agro-climatic zone of India. This widely adapted cultivar occupied a land area of 2.58 m ha, cane area of 53.2% with a cane yield of 81.08 t/ha and sucrose content of 17.99% [34]. The cultivar has become highly susceptible to the red rot causing *C*. *falcatum* pathotypes [2]. The maximum damage(exceeding one billion US dollars)to Co 0238 cultivation in India during 2020 was caused by CF13, a relatively new pathotype of the pathogen [2,35]. Another pathotype prevalent in the zone CF08 is also foreseen to be harmful for newly developed sugarcane cultivars [36]. Therefore, the transgenic Co 0238plants expressing *endochitnase* developed in the present study were bio assayed with CF08 and CF13 responsible for epiphytotic conditions in the zone [35]. The results revealed susceptibility of non-transgenic plant upon inoculation with the pathotypes, whereas the transgenic plant designated as Chit 3–13 displayed resistant reaction. The remaining transgenic plants exhibited moderately resistant to moderately susceptible reaction against CF08, and susceptible to highly susceptible reaction against CF13 suggesting differential plant response towards the pathotypes, and CF13 to be more virulent as compared to CF08. Differential host-pathogen interaction studies by Viswanathan et al. [35] also showed CF13 to be highly virulent among other pathotypes. *Trichoderma* spp. *Endochitinase* has been demonstrated to improve resistance in transgenic plants against phytopathogens [17].

The transgenic sugarcane plants were delineated on the basis of leaf reflectance spectra through Hyperspectral Imaging that is known to vary with change in biochemical composition of leaves [37]. We observed that plants with enhanced *endochitinase* expression carried more chlorophyll content and also displayed higher reflectance in the near-IR range in comparison to low transgene expressing plants. Similar results were reported by Dolata badi et al. [38] in transgenic tomato exhibiting enhanced chlorophyll content due to metabolic changes, following expression of *chitinase* and *glucanase* genes as compared to non-transgenic plants. The chlorophyll content is documented to be positively correlated with reflectance spectra in the near-IR range in several plant species [39,40]. In our study, the transgenic plants with higher reflectance and expression (four-fold or higher) also displayed improved resistance against *C*. *falcatum* pathotypes during bioassay; implying early classification of plants into resistant and susceptible on the basis of HSI.

Abundant *C*. *falcatum* hyphae were observed in the middle lamella and vascular cells of inoculated non-transgenic sugarcane plant through optical microscopy in the present study. The pathogen hyphae inside the plant cells are known to secrete cellulases that degrade cell walls [5,6]. However, in the inoculated resistant transgenic plant Chit 3–13, only limited pathogen hyphae could invade the cells pointing towards the role of *Trichoderma* spp. *endochitinase* in restricting hyphal growth of virulent pathotypes. Further, the transgene expression at high level in this plant revealed its inverse correlation with red rot incidence. The observation is supported by study in transgenic banana plants where higher expression of *chit42*resulted in resistant reaction against *Fusarium* wilt [41] suggesting that transgenic plants expressing transgene(s) at high level had improved protection ability from phytopathogens. The red rot resistant transgenic plant Chit 3–13 was phenotypically normal and comparable to non-transgenic plant.

In sugarcane plant, sucrose synthesised in leaf tissue is transferred through phloem to stalk parenchyma cells for storage [42]. *C*. *falcatum* hydrolyses sucrose accumulated in parenchyma cells through production of invertase enzyme that breaks down sucrose into fructose and glucose thus reducing the sugarcane juice sucrose content, purity and commercial cane sugar [43,44]. The presence of turgid sucrose-filled stalk parenchyma cells was confirmed in transgenic plant Chit 3–13 through electron microscopy that displayed restrained trailing of pathogen hyphae and minimal stalk discolouration. The hyphal restrain was likely due to action of geneen coding endochitinase belonging to glycosyl hydrolase family having established role in hydrolysing β-glycoside bond of N-acetyl-d-glucosamine in chitin and glucan polymers [45]. The sucrose content in the inoculated resistant transgenic plant Chit 3–13 was significantly higher as compared to susceptible non-transgenic plant with up to 4-fold reduction in sucrose content losses. The report by Yao et al. [46] also highlighted that the transgenic lines displaying lower sugarcane mosaic virus incidence had considerably enhanced sucrose quantity in comparison to the non-transgenic plants confirming the role of transgene(s) in minimizing sucrose losses. The structural model of the protein in our study revealed active sites involved in the pathogen cell wall lysis. *Trichoderma* spp. Chitinases are more effective as compared to the corresponding enzymes produced by plants in providing resistance against foliar and soil-borne fungal pathogens [17].

The viable endorhizospheric microbial cell count in resistant transgenic plant Chit 3–13 (expressing antifungal gene under the control of CaMV 35S promoter) was low in comparison to non-transgenic (non-inoculated and inoculated) plants. The reduction in rhizospheric microbial diversity has been observed upon constitutive expression of antifungal genes in transgenic plants, this is likely due to modification in structures of microbial nodulation factors and their specificity [47,48]. The roots of transgenic plants expressing antifungal genes are known to exude end ochitinase among other defence proteins [49]; sloughing of cells near root cap zone also discharges transgenic antimicrobial proteins in rhizosphere [48]. Further, end ochitinase exuded from plants remain effective and stable in rhizosphere even in the presence of active soil microflora, with ability to inhibit fungal spore germination [49]. It is anticipated that *C*. *falcatum* entry into roots of resistant transgenic sugarcane plant might be inhibited under field conditions. In addition, indirect action of antifungal proteins including chitinases is through activation of defence responses in plants by generating elicitor-active oligosaccharides that induce phytoalexin build up in plants, thus increasing the defence response [50]. Thus, it is likely that a combination of protective activities might have got initiated in the resistant transgenic sugarcane plant in response to pathogen infection; additional research in this direction may reveal the intricate *endochitinase*-mediated mechanism of resistance in transgenic plants.

## Conclusion

Transgenic sugarcane displaying resistance to red rot was developed in background of Co 0238 cultivar by expressing *endochitinase* gene from *Trichoderma* spp.qRT-PCR revealed up to 6-fold increase in transgene expression as compared to non-transgenic plant. Hyperspectral Imaging of transgenic plants exhibited changed leaf reflectance spectra and vegetative indices that were directly correlated with the transgene expression. The bioassay with virulent *C*. *falcatum* pathotypes i.e. CF08 and CF13 known for epidemic occurrence led to identification of resistant transgenic plant Chit 3–13. The scanning electron microscopy demonstrated prevalence of a few pathogen hyphae in vascular and sucrose-filled stalk parenchyma cells of the resistant plant. Further, the sucrose content losses in inoculated plant Chit 3–13 were curtailed by up to 4-fold as compared to susceptible non-transgenic plant. The transgenic sugarcane is a valuable source of resistance against CF13 (responsible for Co 238 cultivar breakdown in the north-western zone of India) for use in sugarcane breeding programs.

## Supporting information

S1 Fig PCR analysis of putative transformed sugarcane plants. (PDF)

S2 Fig PCR analysis of putative sugarcane plants using *virG* specific primers. (PDF)

S3 Fig Semi-quantitative reverse transcription-PCRanalysis. (PDF)

S4 Fig Standard curve for a) *tubulin*. b) *endochitinase*. (PDF)

S5 Fig Melt curve analysis of a) *tubulin*.b) *endochitnase*. (PDF)

S6 Fig Amplification chart for both *tubulin* and *endochitinase*. (PDF)

S7 Fig Relative expression analysis *tubulin* and *endochitinase* in RT-PCR positive sugarcane plants along with non-transgenic control. (PDF)

S8 Fig Hyperspectral Imaging of sugarcane plants. (PDF)

S9 Fig Root segment culture and endophytic microbial cell countin roots of transgenic sugarcane plantsinoculated with CF13 pathotype. (PDF)

S10 Fig Ramachandran plot of modelled *endochitinase* gene. (PDF)

S11 Fig Verify 3D score of predicted structure. (PDF)

S1 Table Genetic transformation of sugarcane with EHA105 strain carrying *endochitinase* geneunder the control of CaMV35S promoter and NOS terminator. (PDF)

S2 Table Relative *endochitinase* expression in transgenic sugarcane plants. (PDF)

S3 Table C_T_ values of *endochitinase* and *tubulin* in sugarcane plants in triplicates. (PDF)

S4 Table Hyperspectral Imaging of transgenic sugarcane leaves. (PDF)

S5 Table Bioassay of transgenic sugarcane for red rot disease incidence. (PDF)

S6 Table Association between gene expression and disease incidence in transgenic plants assessed using Pearson’s correlation coefficient. (PDF)

S7 Table HR Brix in CF13-inoculated plants. (PDF)
