# Supplementary material for: Expression of Trichoderma spp. endochitinase gene improves red rot disease resistance in transgenic sugarcane
Source: PLoS One. 2024 Sep 16;19(9):e0310306. doi: 10.1371/journal.pone.0310306 (PMC11404804; doi:10.1371/journal.pone.0310306)

**S1 Fig** PCR analysis of putative transformed sugarcane plants. NC refers to negative control without DNA, PC is positive control and refers to plasmid pRI 101-ON harbouring *endochitinase*, NTC refers to non-transgenic control containing pRI 101-ON without *endochitinase*, N1 to N64 refers to putative transformed plants, L refers to benchtop 1 kb ladder (Promega, Cat No. G7541).

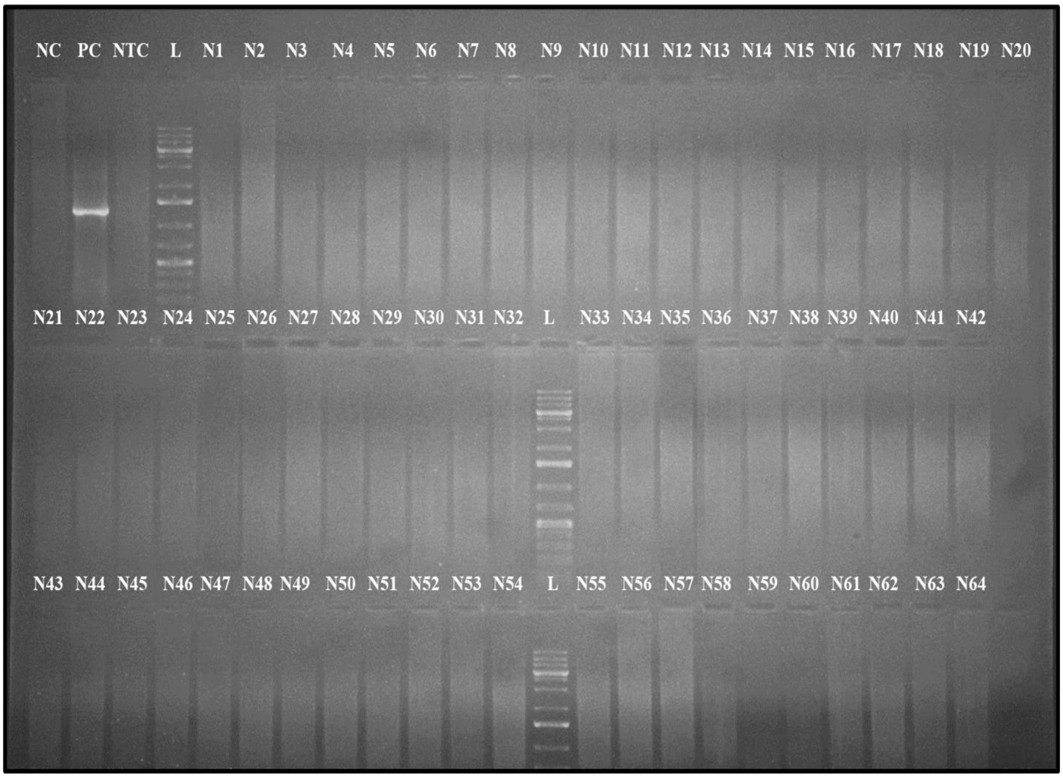


continued...

**S1 Fig** PCR analysis of putative transformed plants. NC refers to negative control without DNA, NTC refers to non-transgenic control containing pRI 101-ON without *endochitinase*, PC is positive control and refers to plasmid pRI 101-ON harbouring *endochitinase*, L refers to benchtop 1 kb ladder (Promega, Cat No. G7541), 1-1 to 1-47 refers to putative transformed plants, 1-9 refers to plant carrying *endochitinase.*

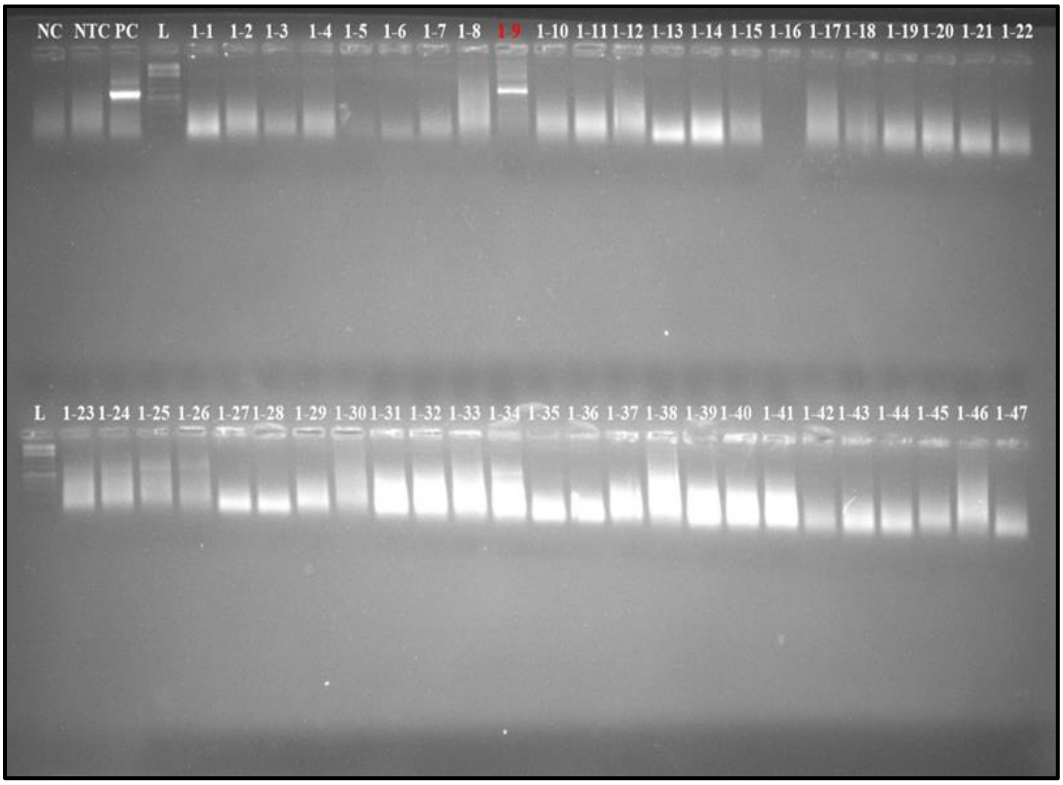


continued...

**S1 Fig** PCR analysis of putative transformed plants. L refers to benchtop 1 kb ladder (Promega, Cat No. G7541), 1-48 to 1-80 refers to putative transformed plants, 1-64 refers to plant carrying *endochitinase.*

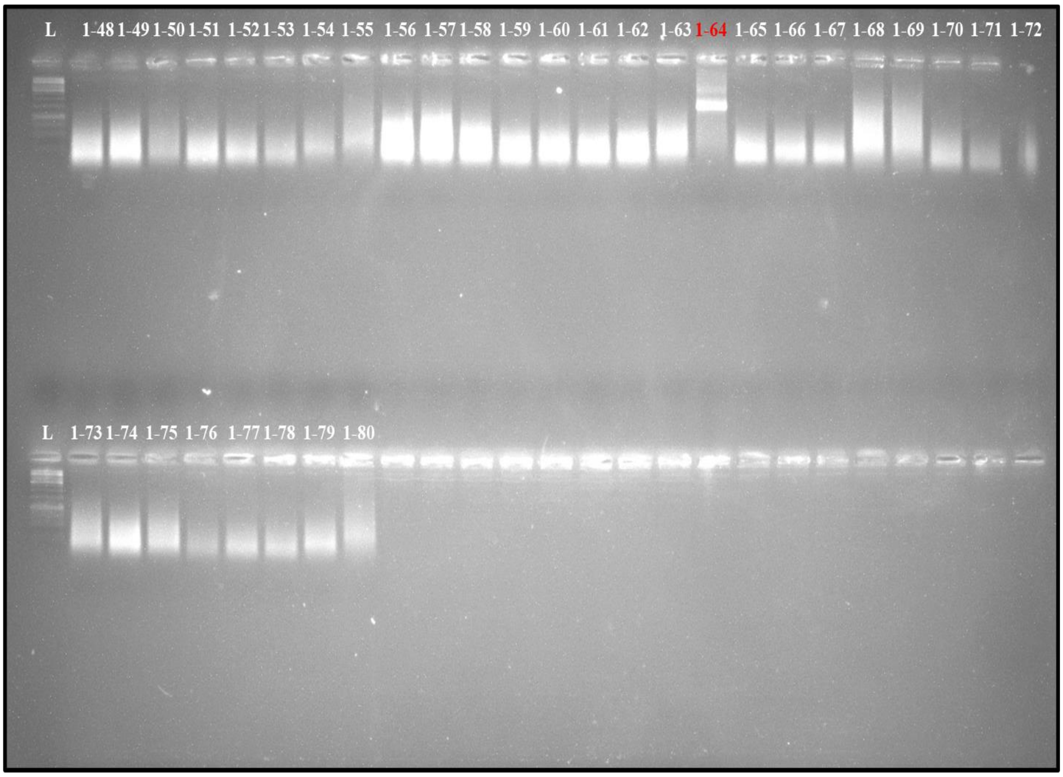


continued...

**S1 Fig** PCR analysis of putative transformed plants. NC refers to negative control without DNA, NTC refers to non-transgenic control containing pRI 101-ON without *endochitinase*, PC is positive control and refers to plasmid pRI 101-ON harbouring *endochitinase*, L refers to benchtop 1 kb ladder(Promega, Cat No. G7541), 2-1 to 2-47 refers to putative transformed plants, 2-39 refers to plant carrying *endochitinase*.

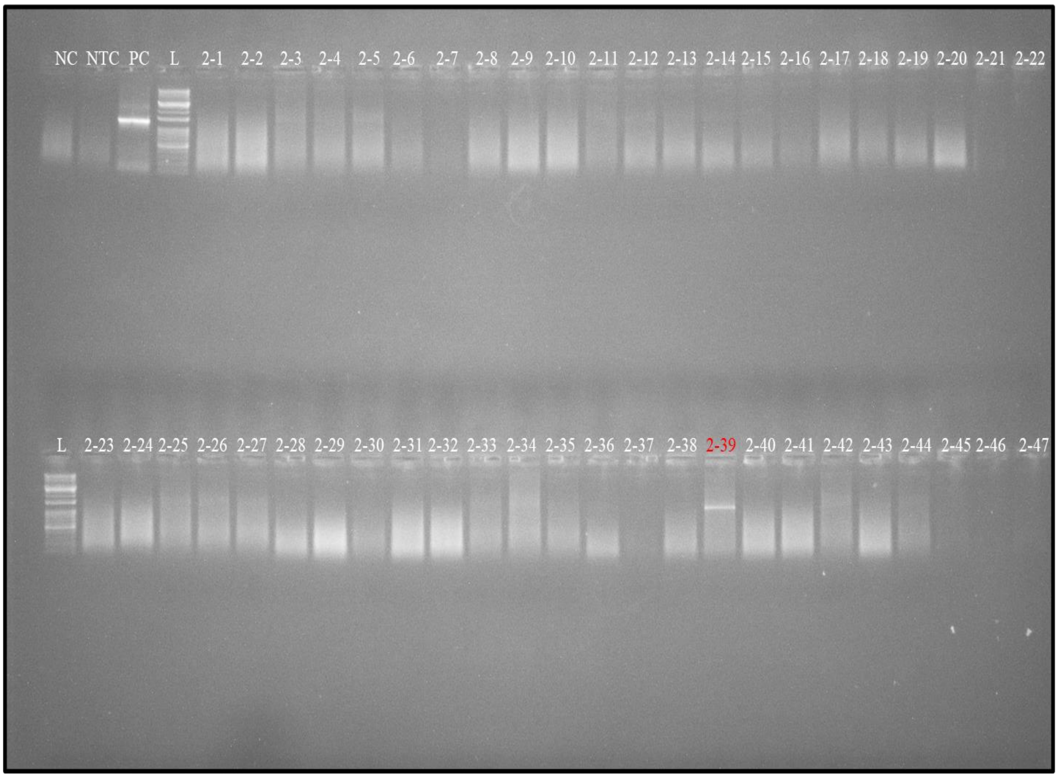


continued...

**S1 Fig** PCR analysis of putative transformed pugarcane plants. L refers to benchtop 1 kb ladder (Promega, Cat No. G7541), 2-48 to 2-83 refers to putative transformed plants, 2-56 refers to plant transformed with *endochitinase.*

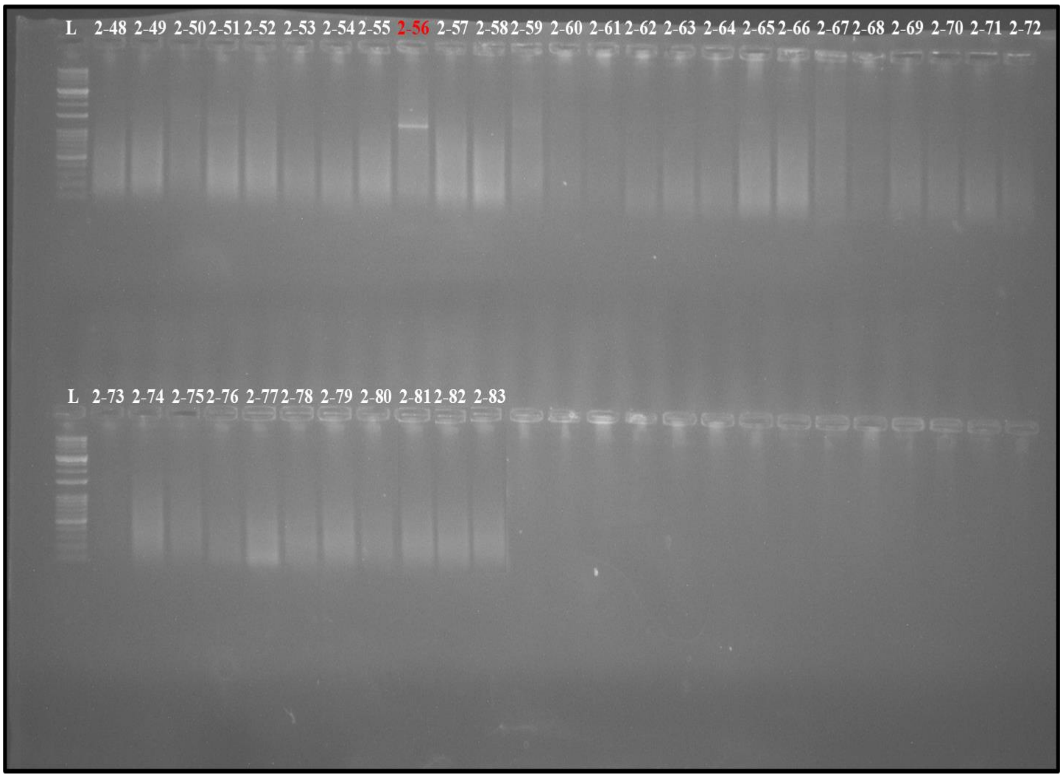


continued...

**S1 Fig** PCR analysis of putative transformed plants. NC refers to negative control without DNA, NTC refers to non-transgenic control containing pRI 101-ON without *endochitinase*, PC is positive control and refers to plasmid pRI 101-ON harbouring *endochitinase*, L refers to benchtop 1 kb ladder(Promega, Cat No. G7541), 3-1 to 3-47 refers to putative plants, and 3-13, 3-30, 3-45 refer to plants carrying *endochitinase.*

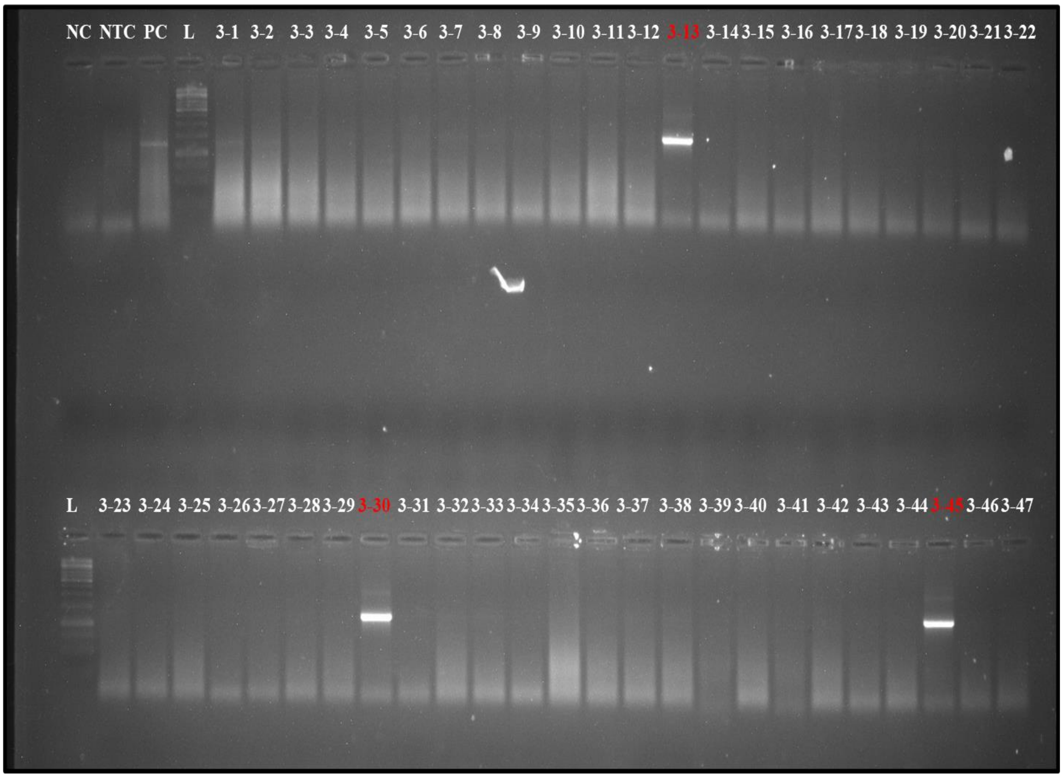


continued...

**S1 Fig** PCR analysis of putative transformed plants. L refers to benchtop 1 kb ladder(Promega, Cat No. G7541), 3-48 to 3-88 refers to putative plants.

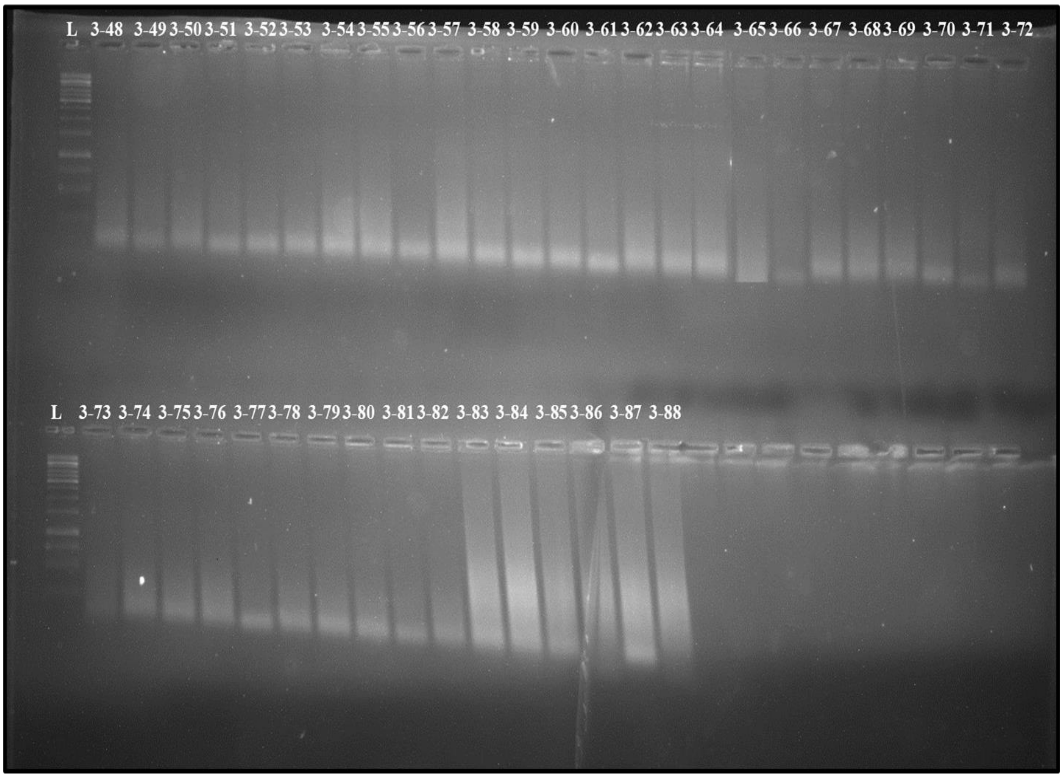


continued...

**S1 Fig** PCR analysis of putative transformed plants. NC refers to negative control without DNA, NTC refers to non-transgenic control containing pRI-101 ON without *endochitinase*, PC is positive control and refers to plasmid pRI 101-ON harbouring *endochitinase*, L refers to benchtop 1 kb ladder (Promega, Cat No. G7541), 4-1 to 4-47 refers to putative plants, 4-9 refers to plant carrying *endochitinase.*

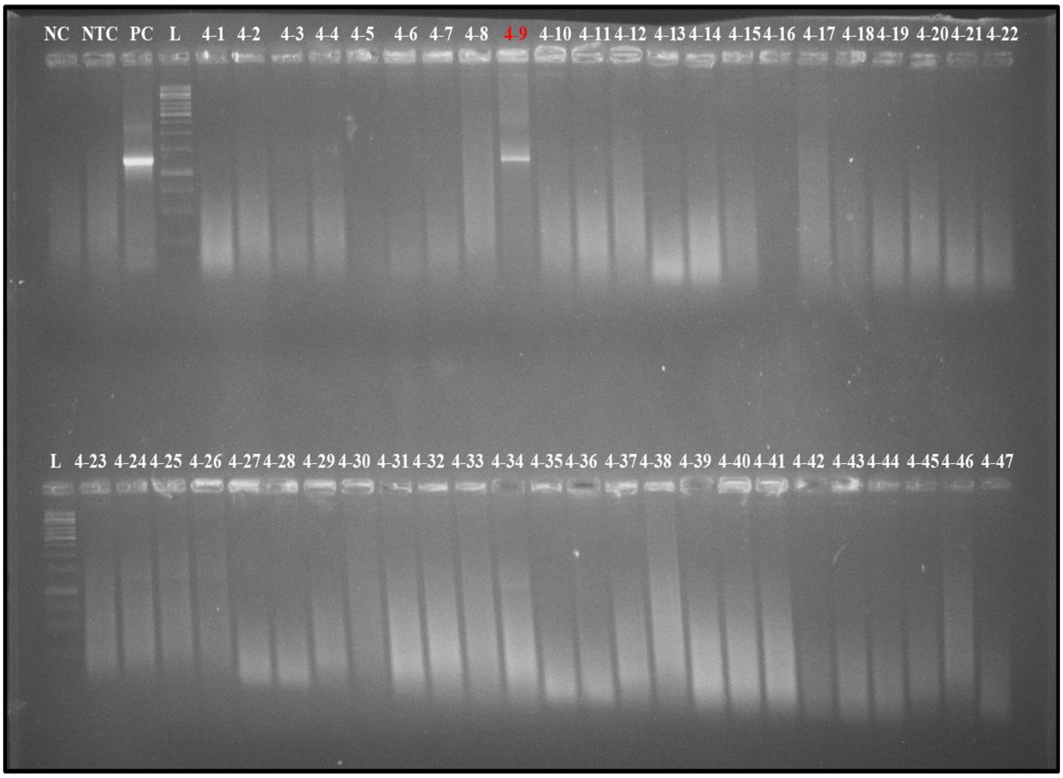


continued...

**S1 Fig** PCR analysis of putative transformed plants. L refers to benchtop 1 kb ladder (Promega, Cat No. G7541). 4-48 to 4-97 refers to putative plants, 4-81 refers to plant carrying *endochitinase.*

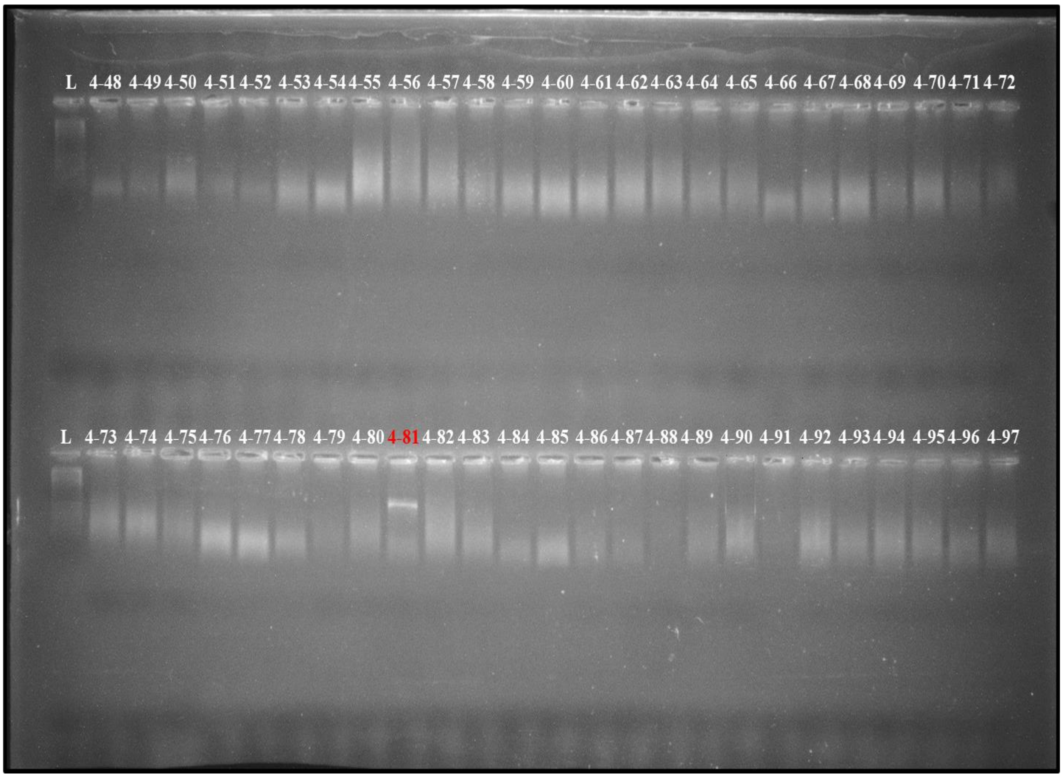


continued...

**S1 Fig** PCR analysis of putative transformed plants. NC refers to negative control without DNA, NTC refers to non-transgenic control containing pRI 101- ON without *endochitinase*, PC refers to plasmid pRI 101-ON harbouring *endochitinase*, L refers to benchtop 1 kb ladder (Promega, Cat No. G7541), 5-1 to 5-47 refers to putative plants.

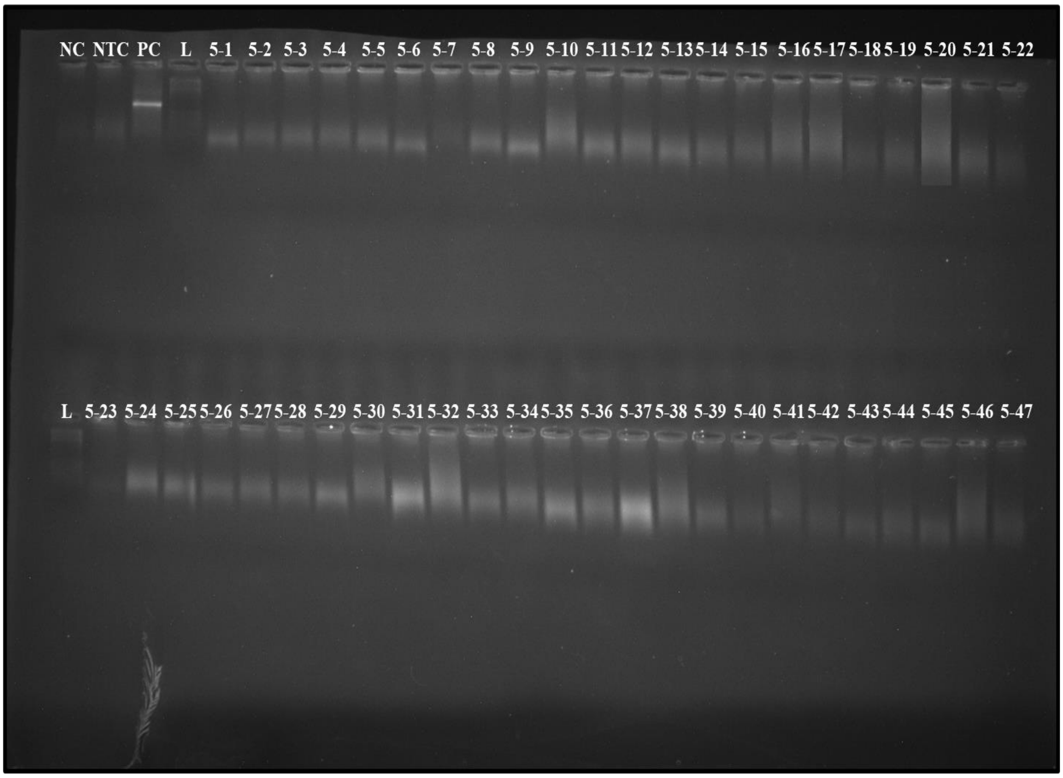


continued...

**S1 Fig** PCR analysis of putative transformed plants. L refers to benchtop 1 kb ladder (Promega, Cat No. G7541), 5-48 to 5-80 refers to putative plants, 5-64 refers to plant carrying *endochitinase.*

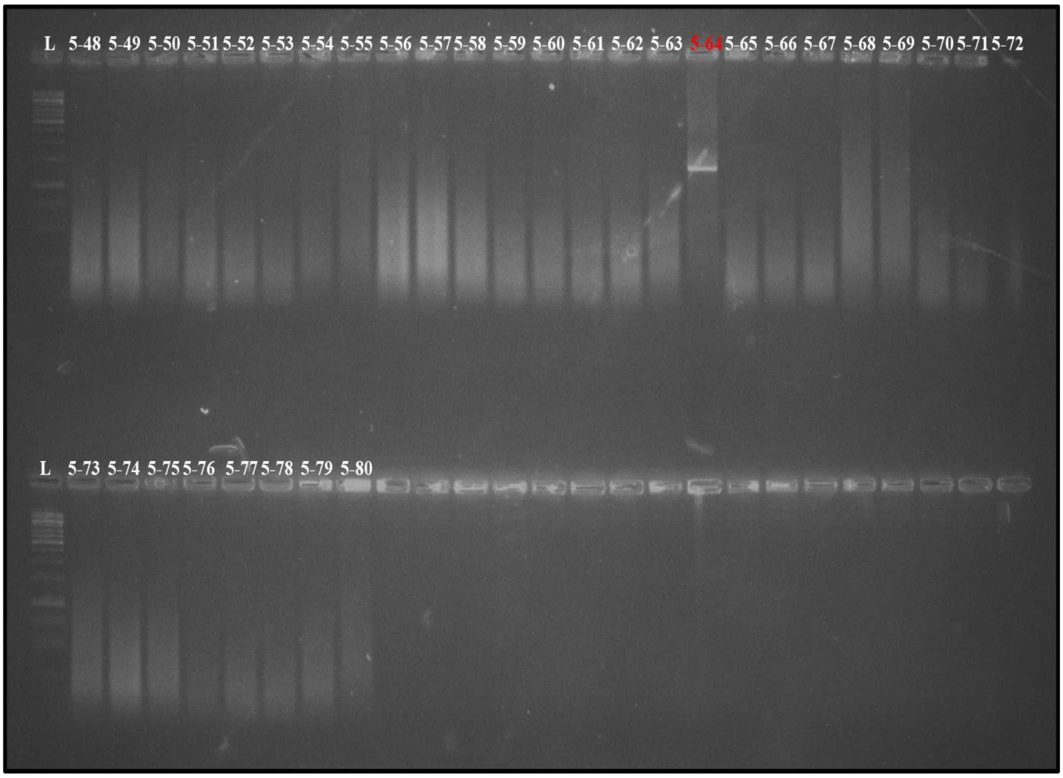

Supplement: S1 Fig — (PDF) [file pone.0310306.s001.pdf]
