# Supplementary material for: Expression of Trichoderma spp. endochitinase gene improves red rot disease resistance in transgenic sugarcane
Source: PLoS One. 2024 Sep 16;19(9):e0310306. doi: 10.1371/journal.pone.0310306 (PMC11404804; doi:10.1371/journal.pone.0310306)

S2 Fig PCR analysis of putative sugarcane plants using *virG* specific primers. L refers to 100 bp ladder (Smobio, Cat. No. DM2100), LBA4404 represents plasmid DNA of *Agrobacterium* strain, the numbers Chit 1-9 to Chit 5-65 refer to putative plants.

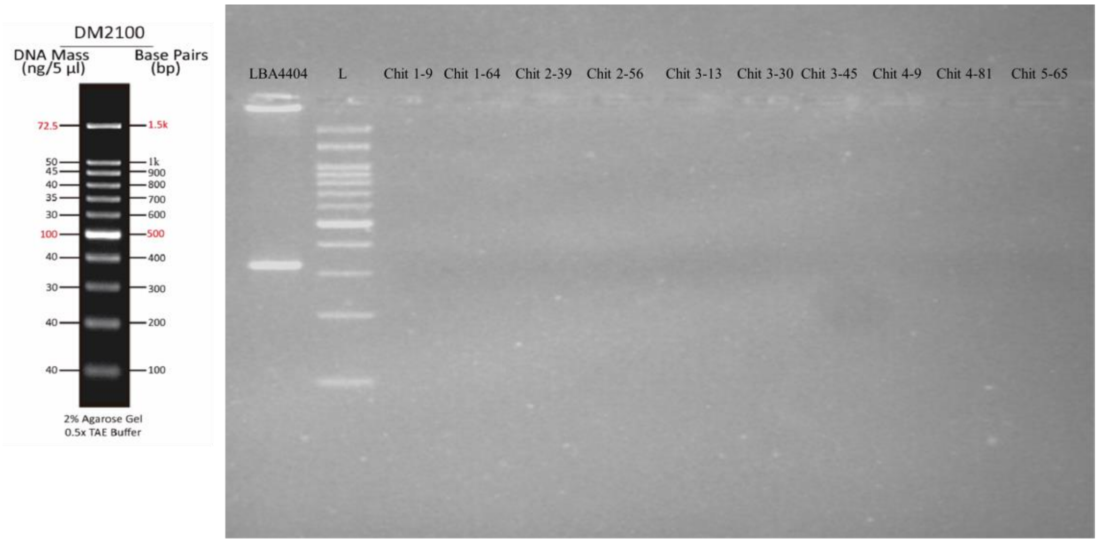

Supplement: S2 Fig — (PDF) [file pone.0310306.s002.pdf]
