# Supplementary material for: Expression of Trichoderma spp. endochitinase gene improves red rot disease resistance in transgenic sugarcane
Source: PLoS One. 2024 Sep 16;19(9):e0310306. doi: 10.1371/journal.pone.0310306 (PMC11404804; doi:10.1371/journal.pone.0310306)

S3 Fig Semi-quantitative reverse transcription-PCR analysis. **a)** RNA isolated from 10 PCR positive plants. 1-9 to 5-65 represents PCR positive sugarcane plants. **b)** cDNA amplified using *26S rRNA* gene specific primers (Ladder used Takara, Cat No. 3407A). **c)** cDNA amplified using *endochitinase* specific primers (Ladder used Promega, Cat No. G7541). NC refers to negative control, PC is positive control and refers to plasmid pRI 101-ON harbouring *endochitinase*, NTC refers to non-transgenic control containing pRI 101-ON without *endochitinase*.

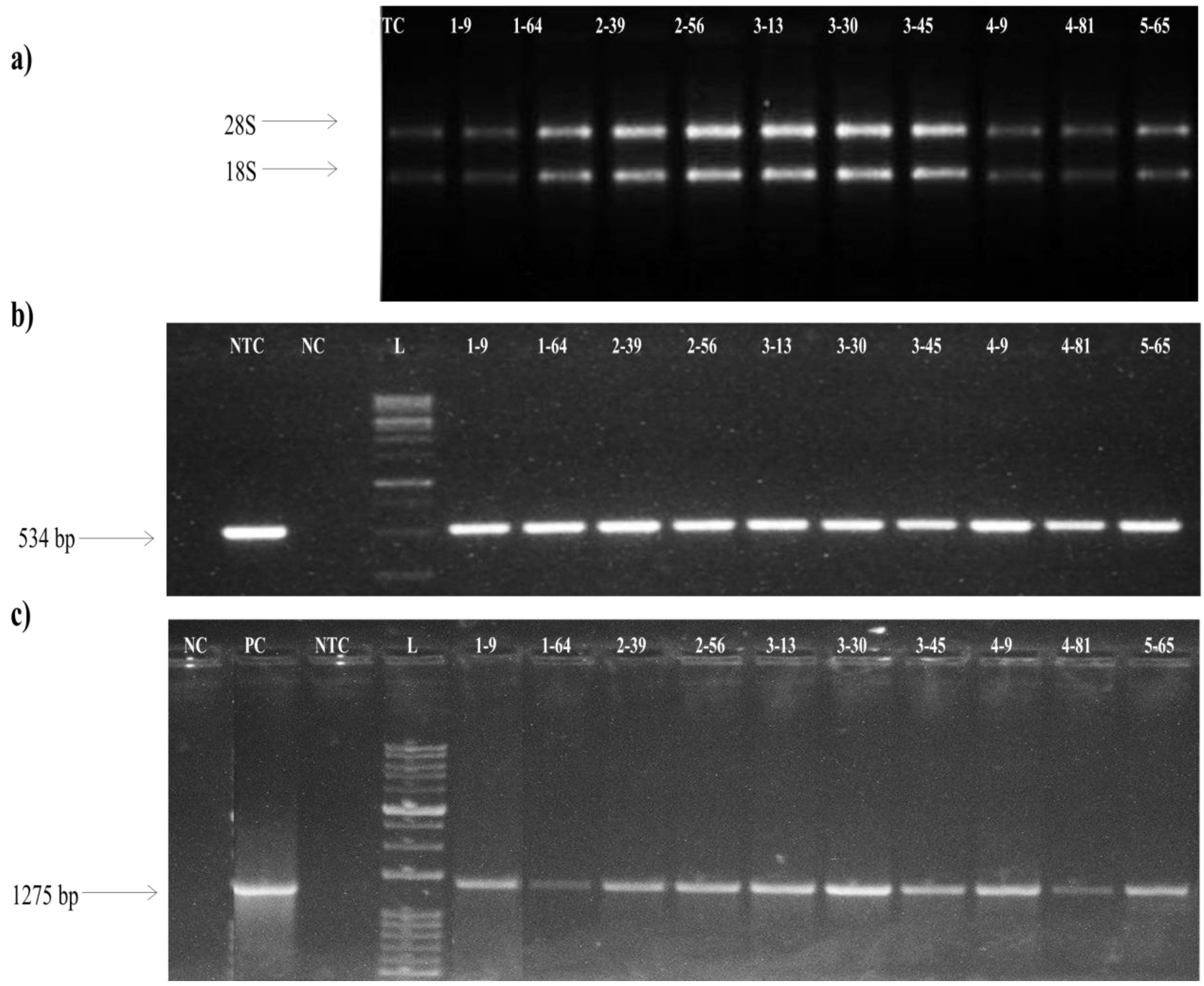

Supplement: S3 Fig — (PDF) [file pone.0310306.s003.pdf]
