# Supplementary figures and images for: Expression of Trichoderma spp. endochitinase gene improves red rot disease resistance in transgenic sugarcane
Source: PLoS One. 2024 Sep 16;19(9):e0310306. doi: 10.1371/journal.pone.0310306 (PMC11404804; doi:10.1371/journal.pone.0310306)

**S4 Fig** Standard curve for **a) tubulin**, **b) endochitinase**.

**a)**

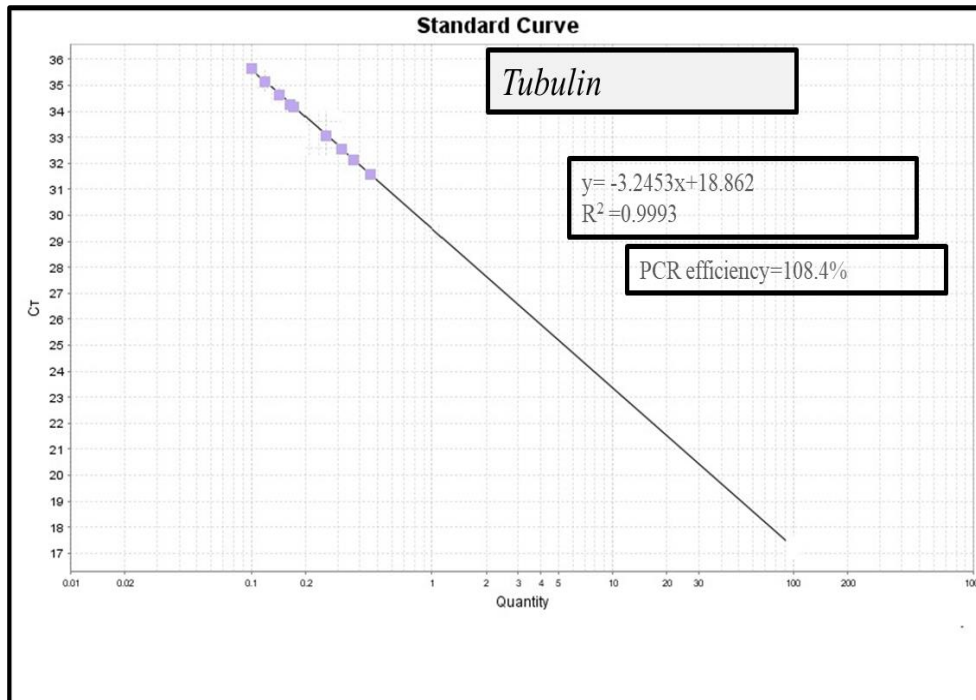

**b)**

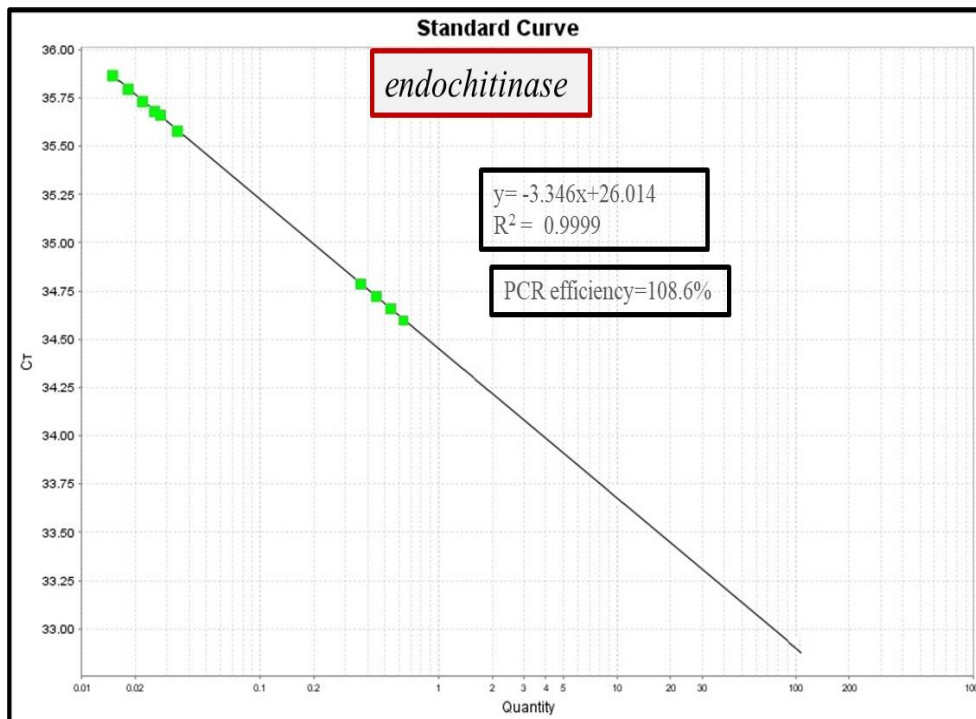

Supplement: S4 Fig — (PDF) [file pone.0310306.s004.pdf]

**S5 Fig** Melt curve analysis of **a) tubulin**, **b) endochitinase**.

**a)**

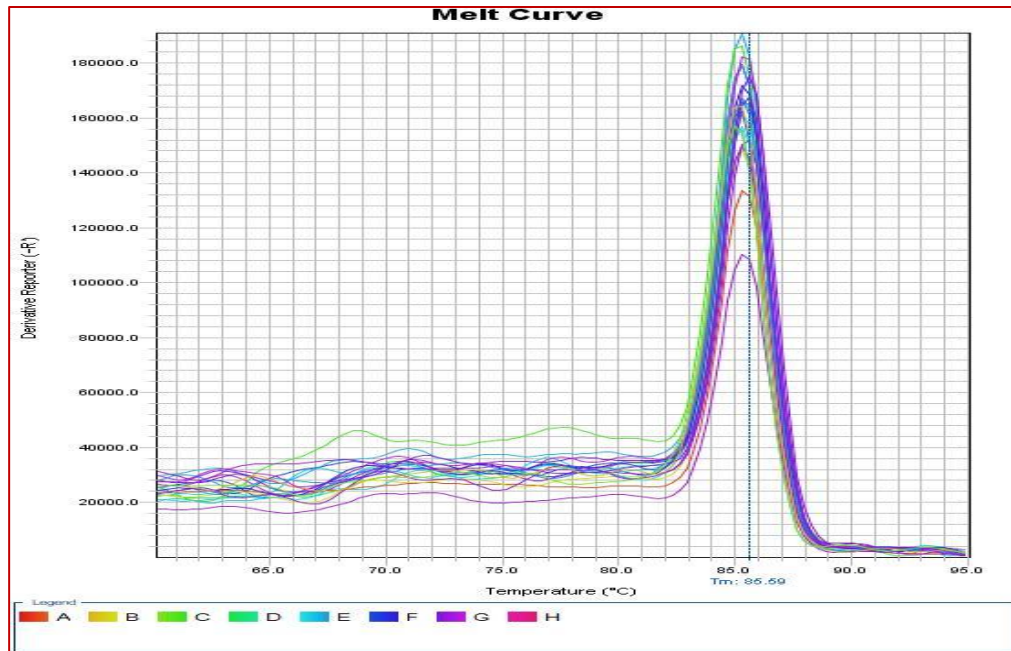

**b)**

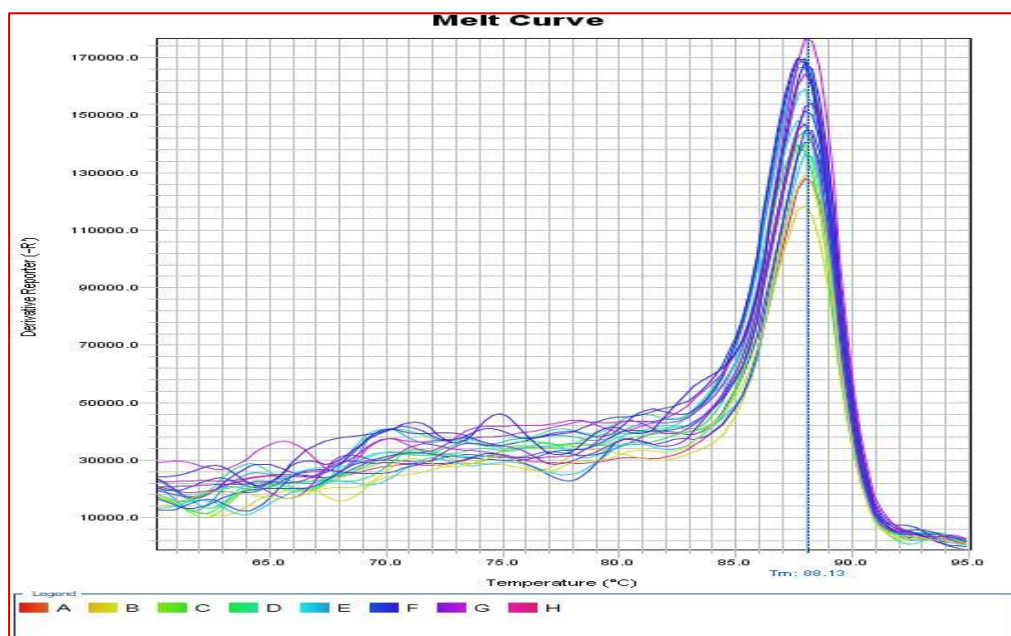

Supplement: S5 Fig — (PDF) [file pone.0310306.s005.pdf]

**S6 Fig** Amplification chart for both *tubulin* and *endochitinase*.

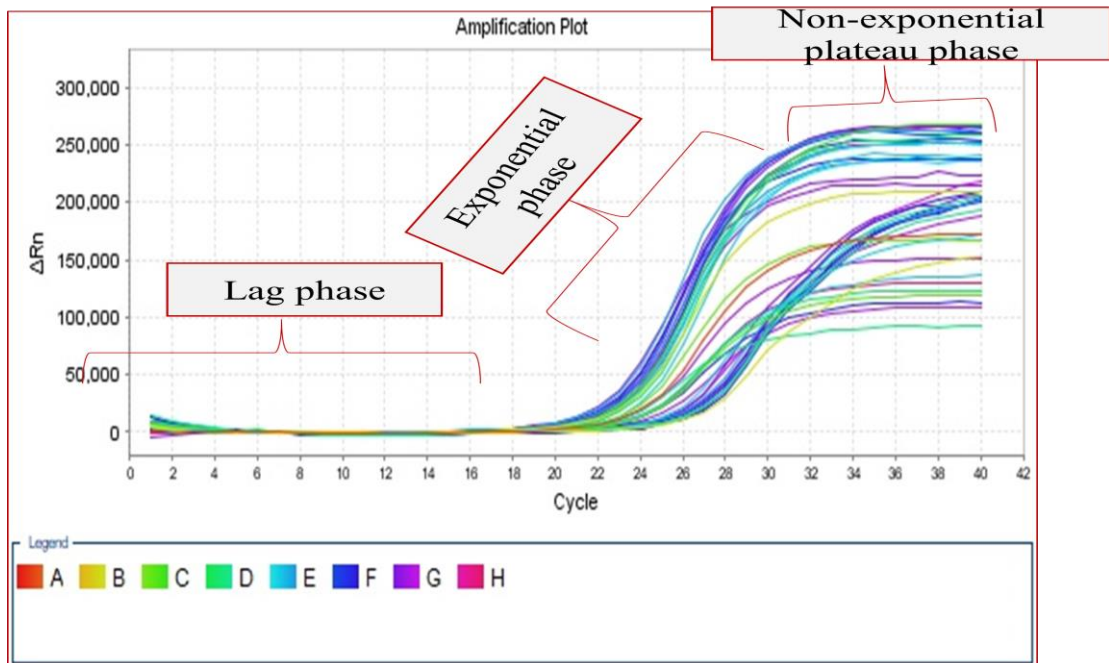

Supplement: S6 Fig — (PDF) [file pone.0310306.s006.pdf]

**S8 Fig** Hyperspectral Imaging of sugarcane plants.

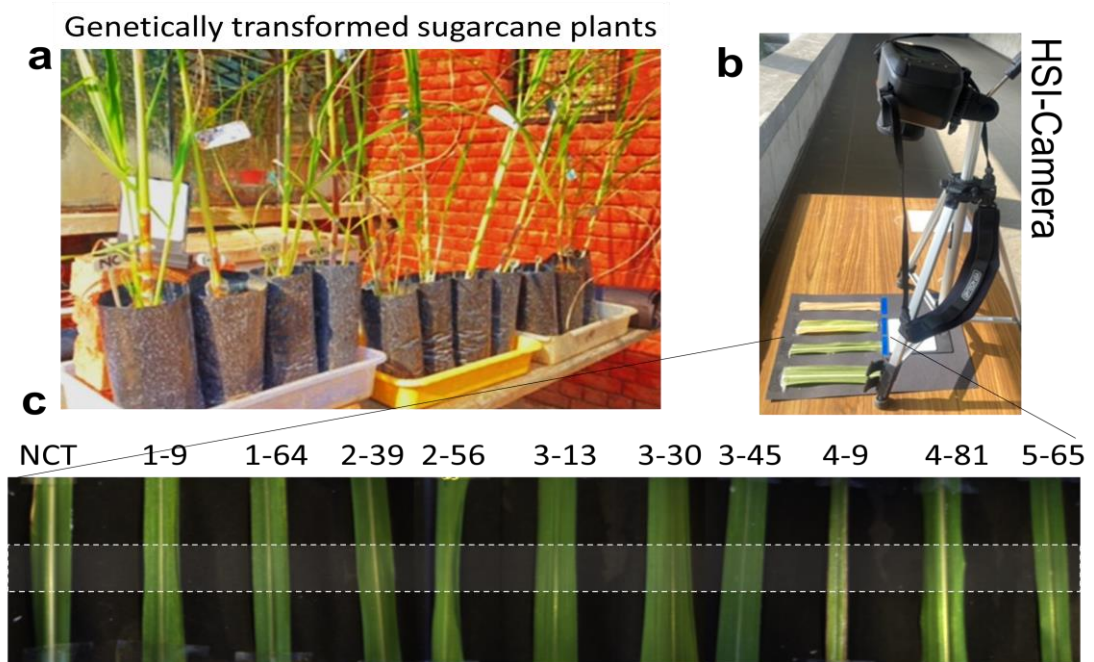

Supplement: S8 Fig — (PDF) [file pone.0310306.s008.pdf]

**S10 Fig** Ramachandran plot of modelled *endochitinase* gene.

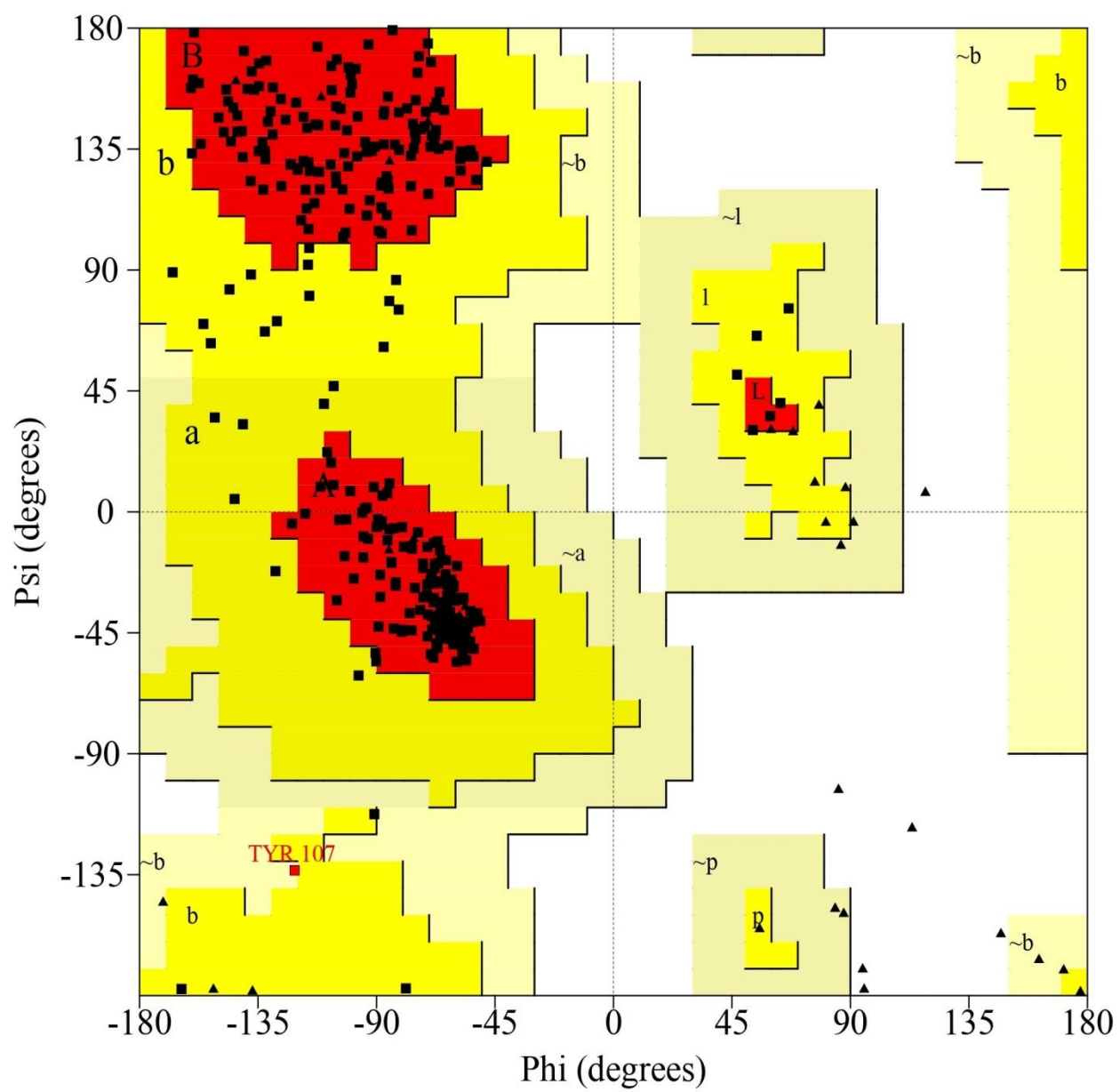

Supplement: S10 Fig — (PDF) [file pone.0310306.s010.pdf]

**S11 Fig** Verify 3D score of predicted structure.

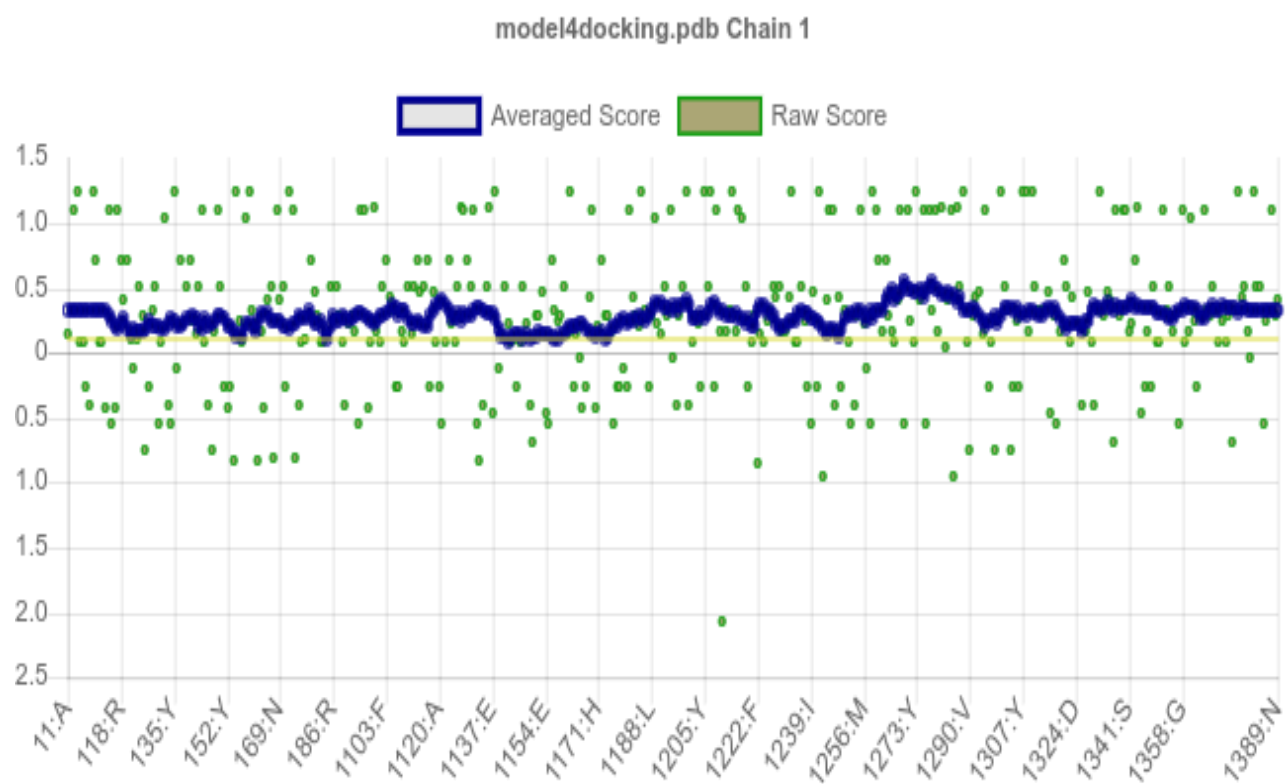

Supplement: S11 Fig — (PDF) [file pone.0310306.s011.pdf]
