# Supplementary material for: Expression of Trichoderma spp. endochitinase gene improves red rot disease resistance in transgenic sugarcane
Source: PLoS One. 2024 Sep 16;19(9):e0310306. doi: 10.1371/journal.pone.0310306 (PMC11404804; doi:10.1371/journal.pone.0310306)

S7 Fig Relative expression analysis of *tubulin* and *endochitinase* in RT-PCR positive sugarcane plants along with non-transgenic control.

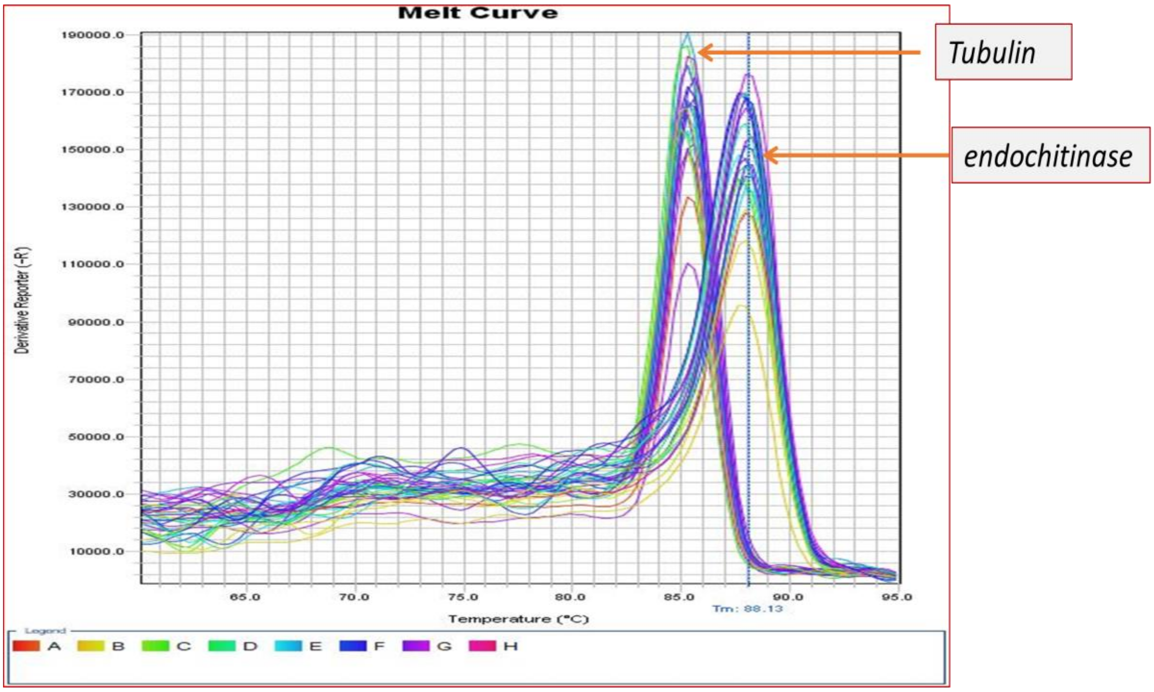

Supplement: S7 Fig — (PDF) [file pone.0310306.s007.pdf]
