# Supplementary material for: Expression of Trichoderma spp. endochitinase gene improves red rot disease resistance in transgenic sugarcane
Source: PLoS One. 2024 Sep 16;19(9):e0310306. doi: 10.1371/journal.pone.0310306 (PMC11404804; doi:10.1371/journal.pone.0310306)
Supplement: S9 Fig — (PDF) [file pone.0310306.s009.pdf]

S9 Fig Root segment culture and endophytic microbial cell count in roots of transgenic sugarcane plants inoculated with CF13 pathotype. **a** Non-transgenic non-inoculated exhibiting high bacterial and fungal growth. **b** Non-transgenic inoculated showing highest bacterial and considerable fungal growth. **c** Inoculated Chit 3-13, resistant to the pathogen, showing low bacterial growth and absence of fungal growth. **d** Endophytic microbial cell count in roots of transgenic sugarcane plants inoculated with CF13 pathotype. **e** Replicated data of endophytic microbial cell count in roots of transgenic sugarcane plants inoculated with CF13 pathotype.

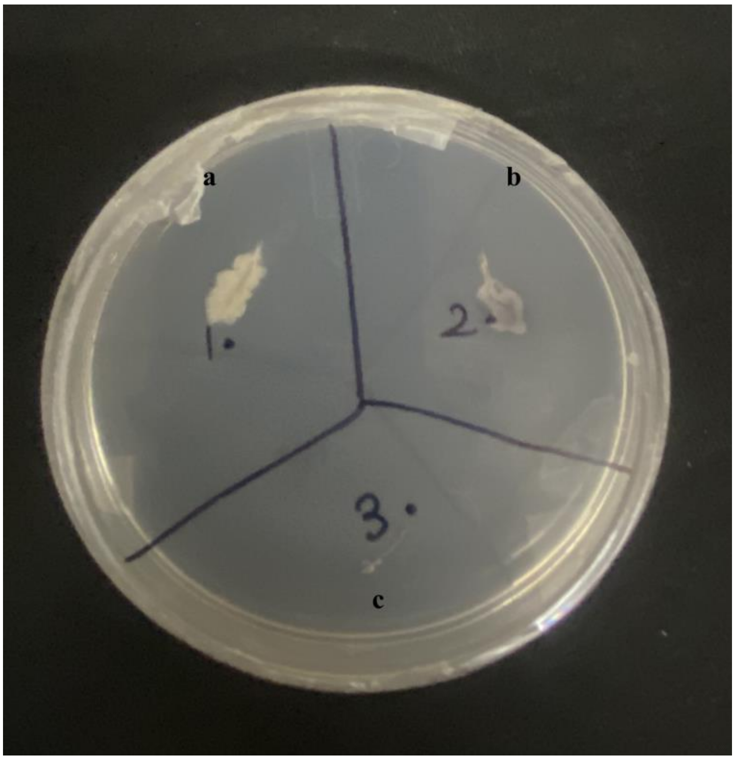

d

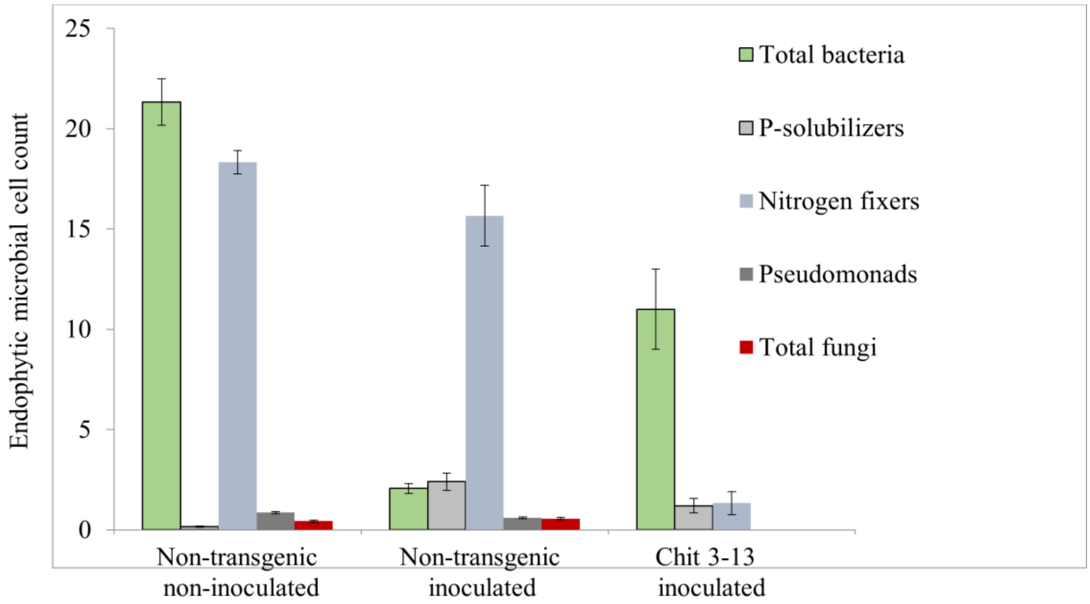

e Replicated data of endophytic microbial cell count in roots of transgenic sugarcane plants inoculated with CF13 pathotype.

	Plant designation	R1	R2	R3	Mean ± SE
**Total bacteria**	Non-transgenic (non-inoculated)	21	19	24	21.33±2.51
	Non-transgenic (inoculated)	1.8	2.1	2.3	2.06±0.25
	Transgenic plant Chit 3-13	9	13	11	11.0±2.00
	Transgenic plant Chit 3-30	12	11	13	12.0±1.0
**P-solubilizers**	Non-transgenic (non-inoculated)	0.1	0.2	0.2	0.16±0.05
	Non-transgenic (inoculated)	2.9	2.1	2.2	2.4±0.43
	Transgenic plant Chit 3-13	1.1	1.6	0.9	1.2±0.36
	Transgenic plant Chit 3-30	1.6	1.7	1.6	1.63±0.05
**Nitrogen fixers**	Non-transgenic (non-inoculated)	18	16	21	18.33±2.51
	Non-transgenic (inoculated)	17	14	16	15.66±1.52
	Transgenic plant Chit 3-13	2	1	1	1.33±0.57
	Transgenic plant Chit 3-30	1	2	1	1.33±0.57
**Pseudomonads**	Non-transgenic (non-inoculated)	1	0.7	0.9	0.86±0.15
	Non-transgenic (inoculated)	0.1	0.1	0	0.06±0.05
	Transgenic plant Chit 3-13	0	0	0	0.0±0.00
	Transgenic plant Chit 3-30	0	0	0	0.0±0.00
**Total fungi**	Non-transgenic (non-inoculated)	0.4	0.4	0.5	0.43±0.05
	Non-transgenic (inoculated)	0.6	0.5	0.6	0.56±0.05
	Transgenic plant Chit 3-13	0	0	0	0.0±0.00
	Transgenic plant Chit 3-30	0	0	0	0.0±0.00

Note: Microbial cell count is presented as 10^3 cfu/ml; Values (of three replicates) analyzed using Microsoft Excel 2007 are shown as mean ± SE.

Transgenic plant Chit 3-13 was having highest transgene expression.

Transgenic plant Chit 3-30 was having lowest transgene expression.
