# Supplementary material for: Expression of Trichoderma spp. endochitinase gene improves red rot disease resistance in transgenic sugarcane
Source: PLoS One. 2024 Sep 16;19(9):e0310306. doi: 10.1371/journal.pone.0310306 (PMC11404804; doi:10.1371/journal.pone.0310306)
Supplement: S1 Table — (PDF) [file pone.0310306.s012.pdf]

S1 Table Genetic transformation of sugarcane cultivar Co 238 with EHA105 strain carrying *endochitinase* gene under the control of CaMV35S promoter and NOS terminator.

No. of experiment	No. of cultured spindle leaf roll segments	No. of agro-infected segments	No. of segments showing shoot regeneration	No. of surviving plantlets
1	60	30	0	0
2	96	64	0	0
3	136	50	0	0
4	80	45	0	0
5	148	130	0	0
6	136	90	0	0
7	112	85	9	5
8	140	85	50	24
9	120	82	37	25
10	240	198	42	20
11	240	180	50	35
12	108	90	30	10
13	264	195	100	80
14	180	136	80	40
15	244	180	40	20
16	88	80	40	25
17	120	95	90	80
18	110	80	80	80
19	100	96	90	90
**Total**	2722	1911	738	534
% Survival		70.0	38.61	27.94
