# Supplementary material for: Expression of Trichoderma spp. endochitinase gene improves red rot disease resistance in transgenic sugarcane
Source: PLoS One. 2024 Sep 16;19(9):e0310306. doi: 10.1371/journal.pone.0310306 (PMC11404804; doi:10.1371/journal.pone.0310306)
Supplement: S2 Table — (PDF) [file pone.0310306.s013.pdf]

S2 Table Relative *endochitinase* expression in transgenic sugarcane plants.

**Plant designation**	**Average C_T *tubulin***	**Average C_T *endochitinase***	**ΔC_T**	**$\Delta\Delta C_T$**	**$2^{-\Delta\Delta C_T}$**
NTC	25.55± 0.01	33.55± 0.03	8.00± 0.02	0.00 ± 0.02	1 (0.90-1.01)
Chit 1-9	25.48± 0.02	32.61± 0.02	7.13± 0.00	-0.87± 0.00	1.83 (1.80-1.87)
Chit 1-64	25.69± 0.02	31.84± 0.02	6.15± 0.00	-1.85± 0.00	3.60 (3.41-3.81)
Chit 2-39	25.45± 0.03	31.18± 0.04	5.73±0.01	-2.27± 0.01	4.82 (4.79-4.86)
Chit 2-56	25.34± 0.01	31.46± 0.03	6.12± 0.02	-1.88± 0.02	3.68 (3.63-3.73)
Chit 3-13	25.13± 0.04	30.35± 0.03	5.22± 0.01	-2.78± 0.01	6.87 (6.82-6.92)
Chit 3-30	25.09± 0.02	33.20± 0.07	8.12± 0.05	0.14± 0.05	0.93 (0.89-0.96)
Chit 3-45	25.46± 0.03	33.30± 0.08	7.84± 0.05	-0.16± 0.05	1.12 (1.08-1.16)
Chit 4 -9	24.96± 0.03	30.53± 0.07	5.57± 0.04	-2.43± 0.04	5.39 (5.24-5.54)
Chit 4-81	26.15± 0.04	31.83± 0.04	5.68± 0.00	-2.32± 0.00	4.99 (4.89-5.09)
Chit 5-65	25.68± 0.01	31.58± 0.06	5.90± 0.05	-2.10± 0.05	4.29 (4.14-4.44)

Values are means of three replicates and the numbers in parentheses represent range of change in *endochitinase* expression as determined by $2^{-\Delta\Delta C_T}$ method. NTC denotes non-transgenic control plant.
