# Supplementary material for: Expression of Trichoderma spp. endochitinase gene improves red rot disease resistance in transgenic sugarcane
Source: PLoS One. 2024 Sep 16;19(9):e0310306. doi: 10.1371/journal.pone.0310306 (PMC11404804; doi:10.1371/journal.pone.0310306)
Supplement: S3 Table — (PDF) [file pone.0310306.s014.pdf]

S3 Table C_T values of *endochitinase* and *tubulin* in sugarcane plants in triplicates.

Well designation	Plant designation	Gene	Cycle threshold (C_T)	C_T mean
A1	NTC	*endochitinase*	33.65	33.55
B1			33.45	33.55
C1			33.55	33.55
D1		*tubulin*	25.49	25.54
E1			25.68	25.54
F1			25.47	25.54
G1	Chit 1-9	*endochitinase*	32.78	32.60
H1			32.62	32.60
A2			32.42	32.60
B2		*tubulin*	25.32	25.47
C2			25.96	25.47
D2			25.15	25.47
E2	Chit 1-64	*endochitinase*	31.85	31.84
F2			32.45	31.84
G2			31.23	31.84
H2		*tubulin*	25.56	25.68
A3			25.87	25.68
B3			25.63	25.68
C3	Chit 2-39	*endochitinase*	31.24	31.17
D3			30.75	31.17
E3			31.54	31.17
F3		*tubulin*	25.12	25.45
G3			25.36	25.45
H3			25.87	25.45
A4	Chit 2-56	*endochitinase*	30.85	31.45
B4			31.54	31.45
C4			31.98	31.45
D4		*tubulin*	25.40	25.34
E4			24.78	25.34
F4			25.85	25.34
G4	Chit 3-13	*endochitinase*	30.23	30.35
H4			30.25	30.35
A5			30.56	30.35
B5		*tubulin*	25.12	25.12
C5			25.24	25.12
D5			25.02	25.12
E5	Chit 3-30	*endochitinase*	32.95	33.20
F5			33.45	33.20
G5			33.21	33.20
H5		*tubulin*	24.59	25.09
A6			25.36	25.09
B6			25.34	25.09
C6	Chit 3-45	*endochitinase*	33.21	33.30
D6			33.41	33.30

E6			33.29	33.30
F6		*tubulin*	25.36	25.46
G6			25.61	25.46
H6			25.42	25.46
A7	Chit 4-9	*endochitinase*	30.25	30.53
B7			30.53	30.53
C7			30.82	30.53
D7		*tubulin*	24.56	24.96
E7			25.36	24.96
F7			24.98	24.96
G7	Chit 4-81	*endochitinase*	31.26	31.83
H7			32.36	31.83
A8			31.87	31.83
B8		*tubulin*	25.98	26.14
C8			26.45	26.14
D8			26.01	26.14
E8	Chit 5-65	*endochitinase*	32.65	31.58
F8			30.56	31.58
G8			31.54	31.58
H8		*tubulin*	26.02	25.67
A9			25.65	25.67
B9			25.36	25.67
