# Supplementary material for: Expression of Trichoderma spp. endochitinase gene improves red rot disease resistance in transgenic sugarcane
Source: PLoS One. 2024 Sep 16;19(9):e0310306. doi: 10.1371/journal.pone.0310306 (PMC11404804; doi:10.1371/journal.pone.0310306)
Supplement: S4 Table — (PDF) [file pone.0310306.s015.pdf]

S4 Table Hyperspectral Imaging of transgenic sugarcane leaves**. a)** Reflectance data. **b)** GCI and GNDVI. **c)** Fold change in expression, GCI and GNDVI. **d)** Covariance between expression, GCI and GNDVI. **e)** Pearson's correlation coefficient (Expression *vs* GCI). **f)** Pearson's correlation coefficient (Expression *vs* GNDVI).

a) Hyperspectral data from leaves of transgenic sugarcane plants

λ	Chit 5-65	λ	Chit 4-81	λ	Chit 4-9	λ	Chit 3-45	λ	Chit 3-30	λ	Chit 3-13	λ	Chit 2-56	λ	Chit 2-39	λ	Chit 1-64	λ	Chit 1-9	λ	Blank	λ	NTC
398.98	0.07	396.90	-0.01	396.01	0.09	397.78	0.04	396.58	0.29	396.94	0.35	396.94	0.36	398.66	0.16	396.94	0.33	401.01	0.15	398.36	0.13	398.98	0.15
400.57	0.05	398.18	-0.02	397.29	0.07	399.06	0.02	397.22	0.26	397.57	0.31	397.57	0.33	399.29	0.14	397.26	0.31	402.27	0.13	398.68	0.11	399.60	0.12
402.37	0.04	400.10	-0.04	399.22	0.05	400.66	0.00	397.86	0.23	398.20	0.27	398.20	0.29	399.91	0.12	397.57	0.29	403.52	0.11	398.99	0.10	400.22	0.10
406.47	0.03	404.39	-0.04	402.49	0.04	404.17	-0.01	398.49	0.20	398.83	0.22	398.83	0.25	400.53	0.09	398.20	0.27	404.77	0.08	399.30	0.08	400.85	0.08
410.07	0.03	407.78	-0.03	406.11	0.05	408.65	-0.01	399.13	0.18	399.45	0.18	399.45	0.22	401.15	0.07	398.20	0.24	407.28	0.06	399.61	0.07	401.47	0.06
414.49	0.03	410.45	-0.01	410.18	0.05	414.08	0.00	399.77	0.15	400.71	0.15	400.71	0.19	401.46	0.05	398.83	0.23	411.04	0.05	398.68	0.14	402.72	0.04
419.59	0.04	413.86	0.01	413.64	0.06	417.92	0.01	400.41	0.13	402.28	0.12	402.28	0.18	402.71	0.03	398.83	0.20	414.80	0.05	404.69	0.07	403.96	0.01
424.05	0.05	418.02	0.03	417.33	0.06	422.23	0.02	401.05	0.12	403.85	0.10	403.22	0.16	404.73	0.01	399.46	0.18	418.56	0.05	404.31	0.08	405.83	-0.01
428.30	0.05	421.33	0.03	421.66	0.06	426.39	0.03	401.69	0.10	406.05	0.08	403.85	0.15	408.31	0.01	399.46	0.15	422.32	0.05	408.07	0.05	408.95	-0.02
432.44	0.05	427.31	0.05	426.15	0.06	430.45	0.03	402.65	0.08	408.25	0.07	404.48	0.14	411.42	0.00	400.40	0.13	426.08	0.06	410.58	0.05	412.68	-0.02
436.27	0.05	431.58	0.06	431.28	0.06	434.54	0.04	403.93	0.06	412.34	0.05	405.74	0.11	415.46	0.00	401.66	0.11	429.84	0.06	413.40	0.07	416.42	-0.02
440.28	0.06	434.67	0.06	434.64	0.06	439.33	0.04	406.49	0.05	415.79	0.04	408.88	0.10	419.51	0.01	403.55	0.08	433.60	0.06	416.22	0.07	419.85	0.00
445.51	0.06	439.47	0.07	440.25	0.06	443.49	0.04	409.36	0.04	420.19	0.05	412.40	0.09	423.24	0.03	405.75	0.06	437.36	0.06	419.04	0.05	423.28	0.02
449.97	0.06	443.31	0.07	443.62	0.06	447.80	0.05	412.24	0.03	424.09	0.05	415.58	0.09	427.16	0.03	409.02	0.04	441.12	0.06	422.80	0.06	427.02	0.03
454.18	0.07	447.79	0.08	447.88	0.07	451.96	0.05	416.87	0.02	428.36	0.05	419.88	0.08	430.52	0.05	413.05	0.04	444.88	0.06	425.93	0.04	430.75	0.04
461.19	0.07	453.34	0.08	452.63	0.07	456.27	0.06	421.13	0.03	432.45	0.06	424.21	0.07	434.28	0.05	416.70	0.04	448.64	0.06	429.07	0.04	434.49	0.05
464.63	0.07	459.64	0.09	456.47	0.08	460.43	0.06	425.13	0.03	436.22	0.05	428.05	0.07	438.48	0.06	420.54	0.05	452.40	0.06	432.83	0.05	438.23	0.05
470.69	0.07	462.20	0.09	460.45	0.08	464.27	0.06	428.33	0.03	439.99	0.04	432.45	0.06	441.60	0.06	424.32	0.06	456.16	0.07	436.59	0.04	441.97	0.06
473.87	0.07	467.64	0.09	466.22	0.08	468.93	0.06	432.70	0.03	444.20	0.04	435.69	0.06	446.26	0.07	428.16	0.07	459.92	0.07	440.03	0.04	445.71	0.07
478.02	0.07	471.16	0.09	473.27	0.08	471.30	0.06	436.43	0.03	448.16	0.05	439.26	0.05	450.55	0.08	431.87	0.07	463.68	0.08	443.48	0.05	449.45	0.07
485.03	0.07	477.57	0.09	476.48	0.08	476.73	0.06	440.05	0.03	451.61	0.05	443.13	0.06	454.35	0.09	435.83	0.07	467.44	0.09	447.24	0.04	453.19	0.07
493.44	0.08	483.90	0.09	481.61	0.08	480.89	0.06	444.10	0.03	455.64	0.05	447.84	0.06	458.71	0.10	439.11	0.06	471.20	0.09	451.00	0.04	456.92	0.09
497.46	0.08	487.49	0.09	485.13	0.08	484.72	0.06	448.68	0.03	459.53	0.06	451.36	0.07	463.06	0.11	443.20	0.07	474.96	0.10	454.76	0.04	460.66	0.09
501.39	0.08	491.65	0.09	490.26	0.08	488.56	0.06	452.41	0.04	463.30	0.06	455.54	0.07	467.11	0.11	446.66	0.08	478.72	0.10	458.52	0.04	464.40	0.09
506.07	0.09	495.39	0.09	493.95	0.09	492.08	0.06	458.80	0.04	466.82	0.07	459.16	0.07	470.84	0.12	450.75	0.08	482.48	0.10	462.28	0.04	468.14	0.10
510.53	0.10	497.73	0.09	497.96	0.10	497.32	0.07	463.22	0.04	470.78	0.07	462.93	0.07	474.32	0.13	454.53	0.08	486.24	0.12	466.04	0.04	471.88	0.10
514.99	0.11	503.88	0.09	501.80	0.11	502.30	0.07	467.33	0.05	474.99	0.07	468.33	0.08	478.30	0.13	458.30	0.09	490.00	0.12	469.80	0.03	475.62	0.10
518.30	0.13	507.76	0.10	507.25	0.13	506.46	0.08	470.95	0.06	478.48	0.08	473.36	0.08	481.21	0.13	462.52	0.10	493.76	0.13	473.56	0.04	479.36	0.11
521.84	0.14	511.98	0.11	509.82	0.14	510.30	0.10	476.01	0.06	482.20	0.09	476.60	0.08	485.15	0.14	466.48	0.10	497.52	0.14	477.32	0.03	483.09	0.12
525.83	0.16	516.51	0.13	512.38	0.16	513.60	0.11	480.45	0.06	486.56	0.09	480.52	0.08	488.57	0.16	470.26	0.10	501.28	0.15	481.08	0.04	486.83	0.12
529.78	0.18	523.02	0.15	514.95	0.17	516.37	0.13	484.80	0.07	489.64	0.10	484.42	0.09	492.24	0.16	473.41	0.11	505.04	0.16	484.84	0.03	490.57	0.12
535.71	0.19	526.76	0.16	517.19	0.19	519.25	0.14	488.42	0.08	494.10	0.10	488.06	0.09	496.82	0.17	477.81	0.12	508.48	0.18	488.60	0.04	494.31	0.13
539.21	0.20	530.70	0.18	519.44	0.20	521.80	0.15	493.27	0.08	497.87	0.11	492.04	0.10	500.70	0.18	481.59	0.12	511.30	0.20	492.36	0.04	498.05	0.14
541.76	0.20	534.87	0.19	521.68	0.22	524.04	0.17	497.59	0.09	501.76	0.12	495.86	0.10	503.50	0.20	485.36	0.13	513.50	0.22	496.12	0.04	501.79	0.15
546.54	0.20	541.05	0.20	524.24	0.23	526.28	0.19	502.70	0.10	505.03	0.13	500.01	0.12	506.68	0.21	489.20	0.13	515.69	0.23	499.88	0.03	505.52	0.16
551.32	0.20	544.95	0.20	526.81	0.25	528.52	0.20	507.02	0.11	509.31	0.15	502.94	0.13	509.83	0.22	493.54	0.14	517.88	0.26	503.64	0.03	509.26	0.18
556.42	0.21	549.91	0.21	530.01	0.26	531.71	0.21	511.01	0.11	512.15	0.16	505.03	0.14	512.32	0.23	497.32	0.14	520.08	0.27	507.40	0.04	512.38	0.19
560.57	0.20	553.69	0.21	533.54	0.28	535.39	0.23	514.21	0.13	514.77	0.18	507.23	0.16	514.39	0.24	501.29	0.15	522.27	0.29	511.16	0.03	515.18	0.21
564.84	0.20	557.40	0.20	536.75	0.29	539.54	0.24	517.72	0.14	517.85	0.20	509.43	0.17	516.26	0.26	504.87	0.16	524.46	0.31	514.92	0.04	518.30	0.23
570.24	0.18	561.33	0.20	541.40	0.29	543.22	0.24	521.24	0.16	521.06	0.22	511.32	0.19	518.75	0.27	508.65	0.17	526.66	0.33	518.68	0.03	521.10	0.24
574.75	0.17	565.44	0.19	545.08	0.30	547.53	0.25	524.44	0.17	523.89	0.24	513.20	0.20	521.55	0.28	511.80	0.18	528.85	0.35	522.44	0.03	523.59	0.26
578.61	0.16	569.44	0.18	549.44	0.30	553.13	0.25	527.31	0.19	527.19	0.26	514.77	0.22	523.73	0.29	514.63	0.20	531.67	0.37	526.20	0.03	526.40	0.27
582.98	0.15	573.60	0.17	552.90	0.30	556.96	0.25	531.95	0.20	530.48	0.28	516.03	0.23	525.28	0.30	517.46	0.22	535.12	0.39	529.96	0.02	529.51	0.29
587.34	0.14	578.27	0.16	556.75	0.31	562.14	0.24	535.62	0.21	533.94	0.30	517.29	0.25	526.84	0.32	519.98	0.24	538.88	0.41	533.72	0.03	532.94	0.31

591.70	0.14	585.44	0.15	560.79	0.30	566.49	0.23	540.80	0.23	536.45	0.31	518.86	0.26	529.33	0.33	523.12	0.25	542.64	0.43	537.48	0.03	536.68	0.32
595.63	0.14	589.07	0.14	564.83	0.30	569.75	0.22	543.94	0.23	541.33	0.33	521.06	0.28	532.44	0.35	526.27	0.27	546.40	0.44	541.24	0.03	540.42	0.34
600.54	0.13	592.81	0.14	568.80	0.29	576.97	0.19	547.77	0.24	544.83	0.33	523.26	0.30	535.55	0.36	530.05	0.29	550.16	0.45	545.00	0.03	544.16	0.34
604.23	0.13	598.89	0.14	572.81	0.28	581.58	0.18	552.25	0.24	548.52	0.34	525.14	0.32	538.91	0.37	534.01	0.31	553.92	0.45	548.76	0.03	547.89	0.35
608.22	0.13	603.69	0.13	577.46	0.26	585.84	0.17	554.48	0.24	552.54	0.35	527.03	0.33	542.52	0.38	537.79	0.32	557.68	0.45	556.28	0.03	551.63	0.36
612.52	0.12	609.13	0.13	581.50	0.25	589.41	0.16	560.02	0.24	556.25	0.36	528.91	0.35	547.37	0.39	541.31	0.33	561.44	0.45	560.04	0.03	555.37	0.36
616.98	0.12	613.77	0.13	587.14	0.25	593.56	0.16	564.71	0.24	560.46	0.35	530.80	0.36	550.69	0.40	545.15	0.34	565.20	0.44	563.80	0.03	559.11	0.36
621.95	0.12	619.38	0.12	591.25	0.24	598.33	0.15	567.70	0.23	564.42	0.35	533.31	0.38	554.21	0.41	548.99	0.35	568.96	0.43	567.56	0.03	562.85	0.36
625.59	0.12	623.00	0.12	596.12	0.24	602.80	0.15	571.59	0.22	568.25	0.34	537.08	0.39	558.20	0.41	552.70	0.36	572.40	0.42	571.32	0.04	566.59	0.36
628.77	0.11	628.13	0.12	601.82	0.24	606.77	0.15	575.74	0.22	572.12	0.32	540.64	0.40	562.05	0.41	556.48	0.35	575.85	0.40	575.08	0.03	570.33	0.35
632.28	0.11	633.78	0.12	606.15	0.23	610.83	0.14	578.78	0.21	575.67	0.31	544.31	0.41	565.41	0.41	560.19	0.35	579.61	0.39	578.84	0.03	574.06	0.35
639.93	0.11	640.82	0.11	609.84	0.23	614.98	0.13	583.25	0.20	579.50	0.30	548.52	0.42	570.08	0.40	563.97	0.35	583.37	0.38	582.60	0.03	577.80	0.34
643.75	0.10	645.37	0.10	613.94	0.22	618.98	0.13	587.41	0.20	583.71	0.29	553.80	0.42	574.62	0.40	567.49	0.34	587.13	0.37	586.36	0.03	581.54	0.33
651.15	0.09	649.25	0.10	617.53	0.21	623.45	0.12	591.14	0.20	587.67	0.28	557.51	0.42	578.17	0.39	571.58	0.33	590.89	0.37	590.12	0.03	585.28	0.33
653.63	0.09	652.67	0.10	622.79	0.21	629.84	0.12	596.29	0.20	591.76	0.28	561.59	0.42	581.84	0.38	575.36	0.32	594.65	0.36	593.88	0.03	589.02	0.33
660.65	0.09	656.67	0.10	626.72	0.20	634.80	0.12	600.90	0.19	597.10	0.28	564.94	0.41	585.95	0.38	579.14	0.31	598.41	0.36	597.95	0.03	592.76	0.33
665.00	0.08	660.35	0.09	631.32	0.21	638.79	0.11	604.67	0.19	600.93	0.27	568.82	0.39	589.37	0.38	582.72	0.30	602.17	0.35	601.40	0.03	596.50	0.33
668.61	0.08	664.19	0.09	634.84	0.20	642.79	0.11	608.50	0.19	605.27	0.27	571.65	0.38	593.54	0.38	586.69	0.29	605.93	0.35	605.15	0.04	600.23	0.33
672.44	0.07	669.31	0.08	639.08	0.20	646.95	0.10	613.62	0.18	609.04	0.27	576.05	0.36	599.32	0.38	590.46	0.28	609.69	0.34	608.91	0.03	603.97	0.33
683.91	0.08	673.58	0.08	643.34	0.19	650.94	0.09	617.14	0.17	612.81	0.26	580.95	0.35	603.06	0.38	594.24	0.29	613.45	0.33	612.67	0.03	607.71	0.32
687.26	0.09	681.16	0.08	647.99	0.17	655.31	0.09	621.93	0.17	616.52	0.25	584.47	0.34	606.79	0.38	598.02	0.28	617.21	0.32	616.43	0.03	611.45	0.32
691.72	0.11	684.89	0.09	652.41	0.16	660.05	0.08	627.36	0.17	620.35	0.25	588.46	0.33	611.08	0.37	601.48	0.27	620.97	0.31	620.19	0.03	615.19	0.32
694.11	0.13	688.31	0.10	656.32	0.15	664.17	0.07	632.05	0.17	624.19	0.25	592.07	0.32	614.88	0.36	605.57	0.27	624.73	0.31	623.95	0.03	618.93	0.32
695.71	0.14	692.20	0.11	660.17	0.14	668.68	0.06	637.59	0.16	627.89	0.25	594.90	0.32	618.61	0.36	609.34	0.26	628.49	0.31	627.71	0.03	622.66	0.32
696.98	0.16	694.60	0.13	663.83	0.13	673.32	0.06	641.17	0.15	632.61	0.25	600.56	0.31	622.90	0.35	613.12	0.26	632.25	0.31	631.47	0.03	626.40	0.32
698.57	0.17	695.88	0.14	667.86	0.12	677.15	0.06	644.63	0.15	635.91	0.24	605.08	0.30	626.76	0.35	616.90	0.25	636.01	0.30	635.23	0.03	630.14	0.33
700.17	0.19	697.16	0.15	671.90	0.11	681.31	0.06	648.46	0.14	639.52	0.24	608.73	0.29	630.12	0.35	620.36	0.24	639.77	0.29	638.99	0.03	633.88	0.32
701.44	0.20	698.44	0.17	676.20	0.11	684.93	0.07	654.34	0.14	643.67	0.23	612.50	0.28	634.10	0.35	624.26	0.24	643.53	0.28	642.75	0.03	637.62	0.32
702.72	0.21	700.05	0.18	680.11	0.11	688.66	0.09	657.84	0.14	647.44	0.23	616.27	0.27	638.21	0.34	628.22	0.24	647.29	0.27	646.51	0.03	641.36	0.32
703.99	0.23	701.97	0.20	683.25	0.12	690.90	0.10	663.00	0.13	651.15	0.22	620.54	0.26	641.63	0.34	632.00	0.23	651.05	0.27	650.27	0.03	645.10	0.32
705.27	0.24	703.89	0.22	685.50	0.14	692.50	0.12	668.28	0.12	654.60	0.22	626.01	0.25	645.18	0.34	635.78	0.23	654.81	0.26	654.03	0.03	648.83	0.31
706.54	0.26	705.49	0.23	687.10	0.15	693.77	0.13	671.80	0.12	658.50	0.21	630.09	0.25	648.91	0.33	639.55	0.22	658.57	0.24	657.79	0.03	652.57	0.31
707.82	0.27	706.77	0.24	688.38	0.17	695.05	0.15	674.67	0.12	662.14	0.20	633.86	0.24	652.99	0.33	643.33	0.21	662.33	0.23	661.55	0.03	656.31	0.31
709.09	0.28	708.05	0.25	689.66	0.18	696.33	0.16	679.79	0.12	667.48	0.20	637.63	0.23	657.34	0.33	647.10	0.21	666.09	0.22	665.31	0.03	660.05	0.30
710.37	0.30	709.33	0.27	690.95	0.20	697.61	0.18	682.02	0.12	671.57	0.20	641.40	0.22	660.86	0.32	650.88	0.20	669.85	0.22	669.07	0.03	663.79	0.30
711.64	0.31	710.61	0.28	692.23	0.22	698.89	0.20	684.90	0.14	675.03	0.20	644.55	0.21	664.59	0.32	654.66	0.19	673.61	0.21	672.83	0.03	667.53	0.30
712.92	0.33	711.89	0.30	693.51	0.24	700.17	0.22	688.31	0.15	679.99	0.20	647.69	0.19	669.17	0.31	658.43	0.19	677.36	0.22	676.59	0.03	671.27	0.30
714.19	0.35	713.17	0.31	694.79	0.26	701.45	0.23	690.65	0.17	683.82	0.22	650.99	0.18	672.56	0.31	661.89	0.18	681.12	0.22	680.35	0.03	675.00	0.30
715.47	0.37	714.45	0.33	696.08	0.28	702.72	0.25	692.57	0.18	686.97	0.24	654.92	0.17	676.48	0.31	665.98	0.17	684.26	0.24	684.11	0.03	678.74	0.30
716.74	0.38	716.05	0.34	697.36	0.30	704.00	0.27	694.49	0.20	689.48	0.26	659.13	0.16	680.31	0.31	669.70	0.17	686.45	0.26	687.87	0.03	682.48	0.31
718.02	0.40	717.97	0.36	698.64	0.32	705.28	0.29	696.09	0.21	691.68	0.28	662.46	0.14	684.25	0.33	673.54	0.17	688.33	0.29	691.63	0.04	685.28	0.33
719.29	0.41	719.57	0.38	699.92	0.34	706.56	0.30	697.69	0.22	693.57	0.31	665.08	0.13	686.12	0.34	677.31	0.17	689.90	0.30	695.39	0.04	688.09	0.34
720.89	0.43	720.85	0.39	701.21	0.35	707.84	0.32	699.29	0.24	695.45	0.33	668.64	0.12	688.30	0.35	681.09	0.18	691.15	0.32	699.15	0.04	690.89	0.36
722.48	0.44	722.13	0.40	702.49	0.37	709.12	0.34	700.56	0.25	697.02	0.35	672.67	0.11	690.48	0.37	684.87	0.19	692.40	0.34	702.91	0.03	693.07	0.38
723.75	0.46	723.73	0.42	703.77	0.38	710.40	0.35	701.84	0.27	698.28	0.37	676.91	0.10	692.34	0.38	688.01	0.21	693.66	0.36	710.43	0.03	695.57	0.40
725.03	0.47	726.61	0.44	705.37	0.40	711.67	0.37	703.12	0.28	699.54	0.40	681.15	0.11	694.21	0.39	690.21	0.23	694.91	0.39	714.19	0.04	697.75	0.42
726.62	0.49	728.22	0.45	706.98	0.42	712.95	0.39	704.72	0.30	700.79	0.42	683.51	0.12	695.76	0.41	692.10	0.25	696.16	0.41	717.95	0.03	699.30	0.44
728.53	0.50	730.78	0.48	708.90	0.43	714.23	0.41	706.64	0.32	702.36	0.44	685.08	0.14	697.32	0.42	693.99	0.27	697.42	0.43	721.71	0.04	700.86	0.46
730.13	0.52	733.34	0.50	710.82	0.45	715.51	0.42	708.56	0.33	703.93	0.47	686.34	0.16	698.88	0.44	695.88	0.29	698.67	0.45	725.47	0.03	703.04	0.48
731.72	0.53	735.26	0.51	712.10	0.46	716.79	0.44	710.15	0.35	705.19	0.49	687.59	0.17	700.12	0.45	697.45	0.31	699.92	0.48	729.23	0.03	705.22	0.50
733.32	0.55	737.18	0.52	713.39	0.48	718.07	0.46	711.43	0.36	706.45	0.51	688.85	0.19	701.99	0.47	698.71	0.33	701.18	0.50	732.99	0.03	706.78	0.52
734.91	0.56	739.74	0.54	714.99	0.49	719.35	0.47	712.71	0.38	707.71	0.53	690.11	0.21	703.85	0.48	699.97	0.36	702.43	0.52	736.75	0.03	708.34	0.53
737.14	0.58	743.42	0.55	717.23	0.51	720.63	0.49	713.99	0.39	708.96	0.55	691.05	0.23	705.10	0.49	701.23	0.38	703.68	0.54	740.51	0.03	710.21	0.55
740.33	0.59	748.06	0.56	719.80	0.52	722.65	0.50	715.27	0.41	710.22	0.57	691.99	0.25	706.34	0.51	702.49	0.40	704.94	0.56	744.27	0.03	711.77	0.57
743.51	0.61	753.18	0.57	722.04	0.54	724.46	0.52	716.23	0.43	711.48	0.59	692.94	0.26	707.59	0.53	703.75	0.43	706.19	0.58	747.97	0.02	713.01	0.59
746.54	0.62	756.39	0.58	723.97	0.55	725.74	0.54	717.19	0.44	712.73	0.62	693.57	0.28	709.14	0.54	705.00	0.45	707.44	0.60	750.22	0.02	714.26	0.61

750.85	0.64	757.67	0.60	725.57	0.56	727.34	0.55	718.46	0.46	713.99	0.65	694.51	0.30	710.70	0.56	706.26	0.46	708.70	0.63	753.67	0.03	715.50	0.63
753.87	0.65	758.63	0.61	730.70	0.57	729.26	0.57	720.06	0.47	715.25	0.69	695.45	0.32	711.94	0.57	707.52	0.48	709.95	0.65	757.43	0.03	716.75	0.66
756.90	0.65	761.08	0.62	734.35	0.58	731.81	0.59	721.98	0.49	716.50	0.72	696.39	0.33	713.19	0.59	708.78	0.50	711.20	0.68	761.19	0.02	718.00	0.68
758.18	0.67	762.79	0.61	736.95	0.59	734.05	0.61	723.58	0.50	717.76	0.74	697.65	0.36	714.43	0.61	710.04	0.52	712.46	0.71	764.95	0.02	719.55	0.70
760.41	0.68	763.43	0.60	740.80	0.59	735.97	0.62	725.18	0.52	719.02	0.77	698.91	0.38	715.68	0.63	711.30	0.55	713.71	0.73	768.71	0.02	721.42	0.72
763.28	0.67	764.07	0.58	744.74	0.60	738.63	0.64	727.73	0.53	720.27	0.80	700.16	0.40	716.92	0.64	712.56	0.57	714.96	0.76	772.16	0.03	723.29	0.74
763.91	0.65	766.63	0.56	748.97	0.61	741.08	0.65	731.89	0.54	721.53	0.82	701.42	0.42	718.16	0.66	713.81	0.60	716.22	0.79	775.60	0.04	725.47	0.76
764.55	0.64	765.99	0.55	752.82	0.60	745.56	0.66	735.62	0.53	722.79	0.83	702.68	0.44	719.72	0.68	715.07	0.62	717.47	0.82	779.36	0.03	728.59	0.77
766.53	0.62	771.43	0.57	757.18	0.60	748.75	0.67	739.40	0.52	724.04	0.85	703.93	0.46	721.59	0.69	716.33	0.66	718.72	0.85	783.12	0.04	732.33	0.77
765.83	0.61	776.55	0.57	760.51	0.61	752.06	0.68	742.60	0.51	725.30	0.88	705.19	0.48	723.45	0.70	717.28	0.68	720.29	0.88	786.88	0.03	736.07	0.78
770.18	0.63	780.39	0.58	764.74	0.61	757.07	0.68	746.75	0.50	726.87	0.89	706.45	0.49	725.01	0.72	718.22	0.71	722.17	0.90	790.64	0.03	739.80	0.79
775.07	0.65	784.43	0.57	767.89	0.61	758.34	0.70	749.79	0.48	729.70	0.91	707.71	0.51	727.19	0.73	719.48	0.73	724.05	0.92	793.78	0.03	743.54	0.78
778.47	0.65	788.72	0.56	772.05	0.61	760.58	0.71	752.99	0.47	733.47	0.90	708.96	0.52	728.64	0.74	720.74	0.76	726.87	0.94	796.91	0.02	747.28	0.77
783.36	0.64	792.72	0.56	776.54	0.61	763.46	0.70	755.54	0.49	737.24	0.89	710.53	0.54	731.23	0.73	722.00	0.78	729.69	0.93	800.67	0.03	751.02	0.77
787.82	0.64	797.57	0.56	779.96	0.60	764.74	0.69	756.50	0.51	740.86	0.88	712.10	0.56	732.79	0.71	723.26	0.81	732.51	0.92	804.43	0.02	754.14	0.77
792.28	0.63	801.20	0.55	783.72	0.59	766.97	0.67	757.14	0.52	743.21	0.86	713.36	0.57	735.77	0.70	724.51	0.84	735.64	0.91	807.56	0.03	755.69	0.79
795.25	0.63	806.07	0.55	787.70	0.60	770.17	0.67	757.78	0.54	745.10	0.84	714.62	0.59	739.32	0.70	725.77	0.86	738.46	0.89	810.69	0.03	756.63	0.81
799.50	0.62	809.65	0.55	791.80	0.59	774.01	0.67	758.42	0.55	748.49	0.83	715.87	0.61	744.30	0.69	727.35	0.88	741.60	0.87	814.45	0.05	757.56	0.83
805.98	0.61	813.75	0.55	795.30	0.59	778.74	0.67	759.06	0.57	751.38	0.82	717.13	0.63	748.22	0.68	730.18	0.90	744.42	0.85	817.27	0.05	758.50	0.86
808.98	0.62	816.89	0.53	799.62	0.59	782.96	0.66	759.70	0.58	754.21	0.80	718.70	0.65	751.76	0.67	733.95	0.91	747.55	0.83	818.84	0.03	761.46	0.88
812.04	0.61	821.85	0.53	803.79	0.59	786.92	0.66	762.26	0.60	756.41	0.80	721.47	0.67	754.25	0.69	737.73	0.91	751.31	0.82	821.03	0.02	760.68	0.90
816.88	0.60	825.79	0.55	807.96	0.58	791.59	0.66	760.98	0.61	757.04	0.83	725.30	0.68	755.50	0.71	741.19	0.91	754.44	0.82	823.85	0.03	760.99	0.91
820.71	0.59	830.23	0.54	811.16	0.57	795.42	0.65	761.94	0.62	757.67	0.86	729.26	0.68	756.74	0.73	745.28	0.90	756.32	0.87	826.99	0.02	762.86	0.86
824.15	0.60	835.65	0.54	814.37	0.58	798.94	0.65	764.17	0.57	758.29	0.90	732.53	0.67	757.67	0.74	749.69	0.89	756.01	0.84	830.75	0.03	763.48	0.84
828.45	0.61	839.23	0.55	818.73	0.58	804.05	0.64	764.81	0.55	758.92	0.93	736.55	0.66	758.30	0.76	753.46	0.88	756.95	0.90	834.51	0.02	764.42	0.82
832.01	0.61	842.50	0.55	824.31	0.58	808.40	0.63	765.45	0.54	759.55	0.96	740.70	0.66	760.47	0.78	756.30	0.89	757.58	0.93	838.27	0.03	765.04	0.80
836.58	0.62	848.45	0.55	827.83	0.58	812.04	0.62	766.09	0.52	761.75	0.99	744.15	0.65	760.47	0.80	757.87	0.93	758.20	0.96	842.03	0.04	765.66	0.78
839.88	0.62	851.46	0.56	832.19	0.58	815.40	0.62	766.73	0.51	762.07	1.00	747.11	0.63	760.47	0.81	757.55	0.91	758.83	0.98	845.16	0.03	766.29	0.76
845.98	0.62	854.21	0.57	835.21	0.59	820.25	0.62	767.37	0.49	762.07	1.02	750.91	0.63	762.65	0.76	758.50	0.95	760.40	1.00	848.29	0.02	768.47	0.74
849.97	0.63	853.06	0.58	838.09	0.60	823.66	0.62	768.33	0.47	762.38	1.04	754.68	0.63	763.27	0.75	759.13	0.98	761.34	1.03	852.10	0.02	772.21	0.72
854.43	0.64	858.18	0.56	842.26	0.61	826.75	0.64	769.93	0.46	763.95	0.97	757.04	0.65	763.90	0.73	759.76	1.00	761.34	1.05	855.81	0.02	775.94	0.71
857.94	0.63	861.70	0.56	845.15	0.61	830.90	0.63	772.96	0.45	764.58	0.93	757.67	0.66	764.52	0.72	760.39	1.03	762.90	0.98	859.57	0.02	779.68	0.71
862.27	0.63	865.54	0.57	847.71	0.60	835.12	0.64	776.00	0.44	765.21	0.90	758.29	0.68	765.14	0.71	762.75	1.06	763.22	0.95	863.33	0.02	782.49	0.70
865.75	0.63	869.39	0.56	851.99	0.60	839.60	0.65	779.52	0.43	765.84	0.88	759.24	0.70	765.76	0.70	763.01	1.09	763.84	0.92	866.15	0.01	785.29	0.68
869.73	0.63	872.27	0.58	855.79	0.60	844.07	0.64	782.71	0.43	766.46	0.85	761.69	0.71	766.70	0.68	764.48	1.03	764.47	0.89	868.97	0.02	789.03	0.68
873.66	0.63	876.91	0.58	859.57	0.61	848.48	0.65	788.25	0.42	767.09	0.83	762.07	0.72	767.94	0.67	765.11	1.00	765.10	0.87	868.97	0.04	792.14	0.67
878.02	0.64	880.91	0.59	863.36	0.62	851.68	0.65	790.07	0.43	767.72	0.81	763.95	0.68	770.43	0.65	765.42	0.98	765.72	0.85	872.11	0.01	795.26	0.66
881.52	0.65	884.43	0.60	866.30	0.62	856.48	0.65	792.62	0.41	768.66	0.78	764.58	0.67	773.23	0.65	766.05	0.95	766.35	0.83	875.05	0.03	798.37	0.66
884.07	0.63	886.99	0.58	869.19	0.61	860.06	0.65	794.86	0.40	769.92	0.76	765.52	0.65	775.41	0.64	766.68	0.93	766.98	0.80	875.24	0.04	800.56	0.64
887.26	0.65	891.15	0.60	873.04	0.62	862.87	0.66	798.38	0.38	771.18	0.73	766.78	0.63	778.52	0.62	767.31	0.91	768.86	0.78	879.00	0.02	803.36	0.62
889.49	0.67	894.03	0.61	877.59	0.62	865.75	0.67	801.89	0.38	773.38	0.71	768.35	0.60	782.25	0.62	767.94	0.89	770.74	0.76	882.13	0.02	807.10	0.63
894.43	0.67	897.72	0.62	881.37	0.64	868.94	0.66	804.58	0.37	776.21	0.69	771.49	0.59	786.29	0.62	768.57	0.87	771.99	0.74	884.33	0.01	809.59	0.63
898.41	0.68	902.36	0.61	885.15	0.64	872.46	0.66	808.61	0.37	779.50	0.68	775.26	0.58	789.41	0.61	769.83	0.85	774.50	0.72	885.89	0.03	811.15	0.65
902.24	0.67	905.35	0.61	890.16	0.64	874.86	0.68	811.96	0.37	783.18	0.68	778.40	0.57	793.45	0.61	771.09	0.83	778.25	0.72	888.40	0.04	813.02	0.68
906.38	0.67	908.50	0.63	894.52	0.65	878.98	0.68	814.36	0.39	786.89	0.66	781.04	0.55	796.87	0.59	773.60	0.80	781.07	0.71	891.22	0.04	815.20	0.69
909.57	0.68	908.12	0.64	897.72	0.66	882.94	0.69	816.60	0.40	789.97	0.64	784.81	0.56	799.67	0.59	777.32	0.79	783.27	0.69	894.04	0.02	817.07	0.67
911.91	0.69	911.96	0.61	901.70	0.66	887.03	0.68	820.56	0.40	793.64	0.64	789.40	0.55	800.92	0.57	781.15	0.79	786.40	0.68	896.86	0.02	819.56	0.66
915.43	0.68	915.80	0.62	904.77	0.66	890.61	0.69	823.63	0.40	797.26	0.64	792.86	0.55	803.09	0.55	784.93	0.79	789.53	0.69	899.68	0.00	822.36	0.64
918.18	0.68	921.18	0.62	906.38	0.68	895.15	0.70	825.97	0.38	801.03	0.63	796.00	0.53	805.58	0.55	788.08	0.78	792.35	0.67	903.44	0.02	824.54	0.62
919.77	0.70	926.05	0.63	908.62	0.69	898.93	0.71	830.02	0.38	803.54	0.61	799.93	0.53	807.14	0.56	791.22	0.76	795.80	0.65	905.95	0.01	827.66	0.61
926.14	0.69	928.29	0.65	912.15	0.67	899.31	0.73	832.58	0.38	806.68	0.59	803.79	0.53	808.69	0.58	795.00	0.74	798.62	0.64	908.45	0.00	831.40	0.62
928.69	0.71	929.57	0.67	915.03	0.68	902.83	0.69	835.26	0.37	810.20	0.59	807.82	0.53	810.87	0.59	798.78	0.73	801.44	0.63	908.45	-0.01	835.14	0.63
931.24	0.73	931.17	0.68	917.92	0.67	902.83	0.68	838.33	0.37	813.91	0.61	811.08	0.53	814.61	0.60	802.49	0.71	805.20	0.65	912.21	0.02	838.88	0.63
934.11	0.74	933.09	0.70	921.44	0.69	905.70	0.70	841.53	0.36	817.68	0.62	814.23	0.54	818.34	0.60	806.27	0.71	807.71	0.63	914.82	0.03	842.61	0.62
936.66	0.73	935.65	0.71	924.33	0.68	907.62	0.72	847.60	0.34	820.51	0.61	817.68	0.53	820.52	0.61	810.10	0.71	810.21	0.61	915.97	0.05	846.35	0.61
938.89	0.71	937.25	0.69	926.25	0.70	909.86	0.73	850.16	0.32	823.02	0.63	821.61	0.55	822.07	0.59	812.62	0.73	812.72	0.63	918.25	0.07	850.09	0.62

942.08	0.72	937.89	0.67	927.86	0.71	913.76	0.74	852.40	0.34	825.22	0.60	824.70	0.54	823.63	0.57	815.14	0.75	813.97	0.65	920.98	0.06	853.83	0.63
943.67	0.74	939.49	0.66	929.46	0.73	917.59	0.74	854.32	0.36	827.11	0.58	826.79	0.53	825.49	0.56	818.92	0.74	816.48	0.68	922.24	0.05	857.26	0.62
944.95	0.75	939.17	0.64	930.42	0.75	921.05	0.73	856.55	0.37	830.19	0.57	829.62	0.51	828.36	0.55	822.69	0.74	820.24	0.67	924.74	0.02	859.75	0.61
947.18	0.77	941.73	0.68	931.06	0.77	926.03	0.74	858.15	0.36	833.08	0.56	833.08	0.51	832.03	0.55	825.21	0.73	822.75	0.65	928.50	0.02	861.31	0.63
947.82	0.78	943.01	0.70	932.99	0.79	928.40	0.75	862.31	0.35	835.91	0.54	837.16	0.51	835.14	0.56	826.47	0.71	824.00	0.63	930.49	0.04	863.86	0.66
949.73	0.76	945.25	0.72	932.99	0.81	930.00	0.77	863.59	0.37	839.68	0.55	840.31	0.51	839.43	0.56	828.67	0.69	825.88	0.61	931.12	0.06	863.49	0.67
951.96	0.74	946.53	0.72	932.99	0.82	931.59	0.78	865.50	0.38	843.13	0.55	845.02	0.51	843.23	0.56	829.61	0.68	829.01	0.60	931.74	0.09	866.91	0.63
957.38	0.74	949.41	0.71	934.91	0.76	932.87	0.80	868.70	0.36	847.22	0.55	847.22	0.50	846.96	0.56	832.45	0.70	832.15	0.59	932.37	0.11	869.72	0.62
958.97	0.72	951.98	0.69	937.47	0.74	934.15	0.81	872.54	0.37	850.93	0.56	850.99	0.50	850.69	0.58	833.96	0.71	835.28	0.57	933.20	0.13	873.15	0.64
961.20	0.72	951.98	0.68	937.79	0.73	936.39	0.83	877.97	0.37	854.82	0.55	853.97	0.51	854.11	0.57	837.80	0.71	838.10	0.58	935.40	0.14	876.88	0.62
966.05	0.71	954.54	0.71	941.32	0.72	939.59	0.81	880.21	0.39	858.85	0.56	856.96	0.50	857.23	0.57	841.26	0.70	840.92	0.59	936.55	0.12	880.62	0.62
968.85	0.72	957.10	0.72	941.96	0.70	939.59	0.80	883.72	0.38	862.30	0.52	859.60	0.49	860.59	0.58	843.14	0.69	844.68	0.60	937.28	0.10	884.36	0.61
971.40	0.74	959.66	0.70	943.89	0.74	941.82	0.83	886.60	0.39	862.30	0.50	862.62	0.48	864.19	0.57	847.24	0.70	848.44	0.60	936.96	0.09	887.16	0.61
972.04	0.75	962.38	0.69	944.53	0.76	946.94	0.84	889.80	0.38	864.82	0.56	864.19	0.50	865.94	0.56	851.01	0.71	852.20	0.59	939.16	0.08	888.72	0.64
975.23	0.73	964.46	0.68	946.83	0.78	949.49	0.86	892.99	0.39	867.52	0.57	866.64	0.52	867.80	0.58	854.47	0.70	855.96	0.58	939.16	0.05	889.97	0.67
975.86	0.71	965.10	0.67	949.34	0.79	951.73	0.88	894.27	0.41	871.41	0.56	870.47	0.51	873.40	0.57	857.93	0.72	859.72	0.58	939.16	0.03	892.27	0.69
979.37	0.73	965.74	0.65	951.58	0.81	951.73	0.89	895.55	0.42	874.24	0.54	874.87	0.49	876.83	0.56	861.71	0.70	863.48	0.58	941.14	0.11	893.40	0.70
983.19	0.74	967.66	0.64	951.58	0.82	953.97	0.85	896.83	0.44	878.64	0.54	878.01	0.51	880.50	0.57	865.49	0.70	867.24	0.60	941.77	0.13	896.20	0.67
985.74	0.76	971.82	0.64	954.79	0.79	954.93	0.84	898.11	0.45	881.47	0.54	882.73	0.51	883.67	0.57	869.26	0.70	871.00	0.57	944.17	0.15	896.20	0.66
988.13	0.77	974.70	0.65	954.79	0.78	961.00	0.84	900.98	0.46	884.99	0.56	885.24	0.52	886.78	0.59	872.72	0.69	874.76	0.57	947.62	0.14	899.94	0.68
992.76	0.77	976.62	0.67	957.03	0.82	962.28	0.82	904.50	0.48	888.63	0.57	888.38	0.53	886.78	0.59	875.56	0.70	877.58	0.59	948.56	0.13	903.68	0.65
996.47	0.76	979.51	0.69	960.88	0.82	964.68	0.80	907.70	0.46	891.52	0.59	891.84	0.53	889.89	0.58	878.39	0.68	880.40	0.61	949.18	0.11	905.86	0.65
997.86	0.78	983.03	0.66	962.16	0.81	968.35	0.80	909.29	0.48	893.10	0.61	894.04	0.55	891.76	0.60	881.91	0.70	884.16	0.60	949.71	0.09	906.48	0.67
998.49	0.80	988.15	0.67	963.44	0.79	973.89	0.80	909.93	0.50	894.35	0.65	894.67	0.56	894.18	0.62	881.53	0.72	886.98	0.62	950.33	0.06	907.41	0.69
1000.62	0.81	989.75	0.69	965.68	0.77	979.54	0.80	911.21	0.52	895.61	0.68	895.61	0.58	897.67	0.63	884.68	0.69	888.86	0.64	950.96	0.04	908.04	0.71
1000.09	0.83	990.07	0.70	966.33	0.76	983.38	0.80	913.77	0.53	898.81	0.69	897.81	0.60	898.29	0.64	886.25	0.70	890.74	0.66	952.94	0.02	908.66	0.73
1002.64	0.79	991.03	0.72	968.25	0.78	985.93	0.81	914.41	0.52	902.52	0.67	898.44	0.62	900.16	0.61	888.46	0.72	893.24	0.68	954.93	0.04	909.91	0.75
		992.95	0.74	970.81	0.80	987.21	0.82	915.05	0.50	905.35	0.69	900.32	0.59	900.78	0.59	891.29	0.73	895.44	0.70	955.55	0.07	912.09	0.77
		995.19	0.73	970.17	0.82	988.49	0.84	915.69	0.49	908.18	0.71	902.52	0.57	902.96	0.58	892.86	0.75	898.01	0.72	956.18	0.10	914.27	0.74
		997.59	0.71	973.06	0.79	990.73	0.86	916.33	0.47	911.01	0.68	902.96	0.56	905.14	0.60	895.70	0.77	897.63	0.73	956.81	0.12	915.52	0.72
		997.91	0.70	974.34	0.77	991.37	0.87	918.56	0.45	912.26	0.70	905.35	0.58	905.76	0.61	899.47	0.78	901.70	0.70	958.58	0.14	918.07	0.70
		999.99	0.74	976.58	0.76	993.29	0.84	920.80	0.47	914.15	0.73	906.29	0.60	906.38	0.62	902.30	0.81	905.46	0.69	960.78	0.14	917.70	0.69
		999.67	0.73	980.11	0.78	995.84	0.82	921.44	0.49	914.78	0.75	908.49	0.63	907.00	0.63	904.82	0.83	907.97	0.72	961.09	0.12	921.75	0.73
		1001.59	0.76	982.68	0.78	994.88	0.80	922.08	0.51	918.23	0.72	910.00	0.63	907.94	0.65	905.76	0.84	909.22	0.75	961.61	0.10	924.24	0.74
		1002.23	0.77	985.24	0.76	998.08	0.85	923.04	0.53	920.75	0.72	913.77	0.64	910.11	0.67	908.91	0.81	911.73	0.76	962.34	0.08	925.17	0.76
				985.24	0.75	999.36	0.87	925.06	0.54	922.00	0.74	914.15	0.65	910.74	0.69	909.02	0.78	915.49	0.76	962.03	0.07	926.11	0.78
				989.09	0.78	1000.64	0.89	926.88	0.56	924.33	0.75	916.35	0.63	912.91	0.66	911.11	0.83	918.62	0.77	962.87	0.05	926.73	0.81
				991.65	0.79	1001.92	0.91	928.15	0.58	926.72	0.75	917.60	0.61	915.40	0.65	912.06	0.86	920.50	0.79	964.63	0.02	927.35	0.85
				992.93	0.80	1003.20	0.93	929.11	0.59	927.35	0.79	918.86	0.59	919.14	0.65	913.32	0.88	921.76	0.81	967.46	0.05	927.04	0.83
				994.22	0.82	1004.15	0.94	929.75	0.61	927.97	0.83	921.06	0.58	921.62	0.66	915.52	0.90	923.01	0.83	967.04	0.03	927.98	0.90
				995.50	0.84			930.39	0.63	928.60	0.87	923.26	0.60	922.87	0.68	918.98	0.86	924.26	0.85	968.09	0.07	927.67	0.88
				996.78	0.86			931.03	0.64	929.23	0.91	924.83	0.62	923.80	0.70	921.50	0.85	925.52	0.89	968.71	0.09	927.67	0.87
				999.35	0.87			931.67	0.66	929.86	0.97	926.40	0.63	924.42	0.72	923.70	0.83	925.20	0.87	969.34	0.11	928.70	0.96
				1001.91	0.89			932.31	0.68	929.86	0.95	927.35	0.65	925.05	0.74	924.33	0.82	926.25	0.93	971.74	0.12	928.29	0.94
				1003.19	0.92			932.95	0.70	930.49	1.02	927.97	0.67	925.67	0.75	926.53	0.85	925.83	0.91	973.52	0.10	928.29	0.92
				1004.15	0.93			933.59	0.72	930.49	1.00	928.60	0.69	926.29	0.77	927.69	0.88	926.87	0.96	974.15	0.08	930.16	1.01
								934.23	0.74	931.12	1.07	929.54	0.71	926.91	0.79	928.42	0.93	927.40	1.00	974.77	0.06	928.91	0.99
								934.87	0.76	931.12	1.06	929.86	0.73	927.53	0.80	928.11	0.91	928.02	1.04	975.40	0.03	930.16	1.05
								935.51	0.78	931.74	1.13	930.49	0.75	928.16	0.82	928.74	0.96	927.71	1.01	977.61	0.02	930.47	1.03
								936.15	0.81	931.74	1.11	931.12	0.78	930.02	0.84	929.68	1.00	928.65	1.08	979.99	0.05	929.53	1.02
								938.38	0.84	932.37	1.19	931.74	0.81	934.38	0.84	929.36	0.97	928.34	1.05	979.58	0.03	933.07	0.99
								938.38	0.86	932.37	1.16	932.37	0.85	939.67	0.84	930.31	1.04	929.28	1.12	980.62	0.07	934.10	1.03
								938.49	0.89	933.00	1.21	933.00	0.88	944.33	0.84	929.99	1.01	928.96	1.10	981.25	0.09	934.83	1.06
								940.62	0.81	933.63	1.25	933.63	0.90	944.96	0.81	930.94	1.08	929.90	1.16	982.08	0.12	935.45	1.08
								942.63	0.78	935.31	1.29	934.26	0.93	945.58	0.79	930.62	1.05	929.59	1.14	984.69	0.13	937.32	1.10
								946.37	0.78	935.52	1.32	934.89	0.95	947.45	0.77	931.57	1.12	930.53	1.21	986.05	0.09	939.50	1.10

								948.29	0.80	935.83	1.35	936.46	0.98	947.45	0.74	931.25	1.10	930.22	1.19	987.41	0.07	939.50	1.08
								948.93	0.83	938.03	1.27	943.37	0.99	947.45	0.72	932.20	1.16	931.16	1.25	988.44	0.05	940.13	1.06
								950.85	0.85	939.91	1.24	945.57	0.97	950.25	0.80	931.88	1.14	930.84	1.23	988.22	0.03	940.75	1.03
								950.85	0.87	942.43	1.22	946.20	0.94	950.25	0.83	933.77	1.21	932.41	1.28	990.54	0.10	941.37	1.01
								950.85	0.89	943.06	1.19	946.83	0.91	951.80	0.78	932.51	1.18	934.92	1.31	990.23	0.08	943.55	0.98
								952.77	0.82	944.94	1.17	947.46	0.89	952.42	0.75	933.77	1.24	937.42	1.30	992.42	0.12	947.29	0.98
								953.41	0.79	944.94	1.14	949.66	0.87	954.08	0.72	934.09	1.22	938.05	1.28	996.18	0.12	950.72	0.98
								955.64	0.78	944.94	1.12	956.57	0.87	954.60	0.70	937.86	1.19	938.68	1.25	999.84	0.12	954.08	1.00
								958.84	0.78	948.08	1.19	958.14	0.90	957.71	0.72	940.27	1.21	939.30	1.23	1002.39	0.10	954.46	1.01
								960.44	0.74	948.08	1.22	960.86	0.92	961.13	0.74	941.01	1.24	939.93	1.20	1002.92	0.08	956.95	0.99
								961.08	0.70	948.08	1.23	960.34	0.95	963.00	0.73	943.08	1.26	941.81	1.18	1002.76	0.06	958.82	0.96
								961.72	0.66	949.97	1.16	960.34	0.98	963.62	0.71	942.74	1.29	944.21	1.21	1003.70	0.03	960.38	0.95
								963.00	0.61	950.60	1.14	962.54	0.90	965.64	0.69	944.78	1.22	945.88	1.25	1004.02	0.14	961.00	0.93
								963.00	0.57	952.80	1.11	963.17	0.88	965.90	0.66	945.41	1.20	946.51	1.27	1004.02	0.13	961.62	0.90
								963.64	0.59	956.57	1.11	965.68	0.86	965.80	0.65	947.93	1.18	946.51	1.29			962.25	0.88
								964.91	0.64	960.34	1.11	967.57	0.83	967.98	0.71	951.71	1.18	948.39	1.23			962.87	0.85
								965.55	0.66	962.85	1.09	968.19	0.81	969.84	0.74	954.70	1.19	949.02	1.21			964.74	0.83
								966.83	0.68	963.80	1.07	968.82	0.79	972.33	0.72	956.01	1.22	951.21	1.19			968.48	0.83
								968.75	0.70	964.42	1.05	970.71	0.77	972.96	0.70	958.16	1.25	954.97	1.18			972.22	0.83
								970.03	0.67	965.05	1.03	973.22	0.75	974.82	0.68	958.16	1.28	958.10	1.18			975.95	0.83
								970.67	0.65	965.68	1.00	973.85	0.73	975.03	0.66	958.00	1.30	959.36	1.16			979.69	0.83
								971.31	0.63	966.31	0.98	974.48	0.71	974.82	0.65	960.20	1.22	959.98	1.13			982.19	0.84
								973.22	0.61	966.94	0.96	975.11	0.69	976.69	0.70	960.83	1.20	960.61	1.09			982.81	0.86
								976.10	0.59	967.57	0.94	977.31	0.67	977.31	0.72	961.78	1.18	961.24	1.06			983.43	0.88
								978.72	0.58	968.19	0.91	979.51	0.68	979.49	0.74	963.03	1.16	963.12	1.03			984.05	0.90
								978.34	0.57	968.82	0.89	980.13	0.70	984.78	0.74	964.29	1.14	966.25	1.02			985.92	0.93
								982.18	0.60	971.02	0.86	980.76	0.72	989.13	0.74	964.61	1.12	969.38	1.01			988.10	0.92
								986.65	0.60	974.79	0.86	981.39	0.75	992.55	0.74	964.92	1.09	973.14	1.01			988.73	0.90
								991.76	0.60	978.56	0.86	983.91	0.76	996.29	0.73	965.24	1.06	976.28	1.01			989.35	0.88
								995.60	0.59	981.39	0.86	985.79	0.74	996.60	0.71	967.44	1.03	977.84	0.98			989.97	0.86
								995.92	0.58	982.65	0.89	987.99	0.72	997.22	0.69	966.92	1.01	978.47	0.96			991.93	0.83
								996.88	0.57	984.85	0.91	987.99	0.69	997.84	0.67	967.96	0.99	979.10	0.94			994.54	0.85
								998.16	0.61	984.85	0.94	987.99	0.67	998.47	0.65	967.13	0.96	979.93	0.92			995.27	0.88
								1000.08	0.64	984.85	0.95	991.13	0.73	999.09	0.62	967.44	0.95	982.54	0.91			997.45	0.90
								1000.08	0.66	987.05	0.90	991.13	0.75			967.44	0.92	986.30	0.91			997.14	0.93
								1000.08	0.67	987.99	0.87	993.96	0.72			970.90	1.08	990.06	0.92			997.14	0.94
								1001.99	0.61	990.50	0.86	993.96	0.69			970.69	1.05	993.82	0.91			999.32	0.87
								1002.63	0.59	993.65	0.86	994.27	0.67			970.59	1.10	997.27	0.92			1000.15	0.85
								1003.27	0.57	995.22	0.89	995.22	0.73			974.05	1.03	999.46	0.95				
										997.42	0.92	995.85	0.76			975.94	1.01	999.15	0.93				
										997.42	0.95	997.31	0.81			977.20	0.99	1000.09	0.98				
										997.42	0.97	997.52	0.85			977.82	0.97	1000.71	1.01				
										999.62	0.89	997.52	0.84			980.09	0.95	1001.34	1.03				
										1001.82	0.86	999.62	0.79			979.71	0.92	1001.97	1.05				
												1001.59	0.75			983.36	0.97						
																982.54	1.00						
																986.01	0.94						
																985.59	0.92						
																987.58	0.97						
																987.89	0.99						
																989.87	1.02						
																992.30	1.00						
																992.93	0.98						
																993.56	0.96						
																994.19	0.94						
																995.76	0.92						

																997.18	0.91						
																998.91	0.93						
																999.54	0.96						
																1001.74	0.98						
																1001.42	1.00						
																1001.42	1.03						
																1003.63	0.95						
																1004.57	0.93						

b)

Plant	Expression	SD (±)	554 nm	554 nm	554 nm	Avg_Green	747 nm	747 nm	747 nm	Avg_NIR	*GCI	**GNDVI
**NTC**	1	0.02	0.25342	0.107	0.107	0.15581	0.68343	0.60097	0.60097	0.62846	3.033502	0.602662
**1-9**	1.84	0.04	0.44242	0.572	0.5601	0.52484	0.87262	0.93956	0.93545	0.91588	0.745065	0.27142
**1-64**	3.6	0.4	0.43415	0.4555	0.53433	0.47466	0.96096	0.94961	0.92692	0.94583	0.992647	0.331695
**2-39**	4.89	0.1	0.01062	0.55543	0.63754	0.4012	0.96149	0.94492	0.98126	0.96256	1.399202	0.411627
**2-56**	3.73	0.02	0.25275	0.16686	0.16084	0.19348	0.54037	0.41934	0.41259	0.45743	1.364224	0.405509
**3-13**	6.86	0.25	0.20848	0.24104	0.2295	0.22634	0.57697	0.63609	0.63879	0.61728	1.727225	0.463408
**3-30**	0.93	0.03	0.22	0.24	0.26	0.24	0.6	0.6	0.69	0.63	1.625	0.448276
**3-45**	1.12	0.07	0.26442	0.23424	0.23872	0.24579	0.67373	0.65473	0.6767	0.66839	1.719354	0.462272
**4-9**	5.42	0.31	0.1959	0.1586	0.121	0.1585	0.4379	0.3373	0.3262	0.36713	1.316278	0.396914
**4-81**	4.99	0.1	0.20991	0.26596	0.23213	0.236	0.77305	0.69543	0.78038	0.74962	2.176356	0.521114
**5-65**	4.28	0.03	0.25798	0.20446	0.3544	0.27228	0.75008	0.81866	0.84294	0.80389	1.952439	0.493983

*Green Chlorophyll Index= (Avg NIR/Avg Green) -1

**Green Normalized Vegetative Index= (Avg_NIR –Avg_Green)/ (Avg_NIR + Avg_Green)

c)

Plant designation	Fold change (Expression)	GCI	GNDVI
NTC	1.00	3.03357	0.60267
1-9	1.84	0.74506	0.27142
1-64	3.6	0.99265	0.3317
2-39	4.89	1.39921	0.41163
2-56	3.73	1.3642	0.40551
3-13	6.86	1.72724	0.46341
3-30	0.93	1.87664	0.44828
3-45	1.12	1.7193	0.46226
4-9	5.42	1.13853	0.39692

4-81	4.99	2.17636	0.52111
5-65	4.28	1.95245	0.49398

d)

Plant designation	Fold change (Expression)	Covariance (GCI)	Covariance (GNDVI)
NTC	1.00	-0.31684	-0.03783
1-9	1.84	-0.13739	-0.02523
1-64	3.6	-0.00509	-0.00082
2-39	4.89	0.03105	0.00319
2-56	3.73	-0.00555	-0.00062
3-13	6.86	0.02424	0.00798
3-30	0.93	-0.05383	-0.00261
3-45	1.12	-0.01562	-0.00546
4-9	5.42	0.08817	0.00697
4-81	4.99	0.07093	0.01126
5-65	4.28	0.02122	0.00395

Covariance amongst two variables was be calculated as

$$\frac{\sum(\boldsymbol{x}-\overline{\boldsymbol{x}})*(\boldsymbol{y}-\overline{\boldsymbol{y}})}{\boldsymbol{N}}$$

where on x-axis was expression (chitinase) change and y was green chlorophyll index(GCI) and x and y was mean of Expression change and mean of GCI, respectively. Similarly, covariance amongst GNDVI and expression was estimated. In a similar manner, covariance amongst GNDVI and fold change in expression was estimated.

e) Pearson's correlation coefficient (Expression *vs* Green Chlorophyll Index [GCI])

X Values: Geno	Expression	Y Values (GCI)
1-9	1.84	0.74506
1-64	3.6	0.99265
2-39	4.89	1.39921
2-56	3.73	1.3642
3-13	6.86	1.72724
4-9	5.42	1.13853
4-81	4.99	2.17636
5-65	4.28	1.95245

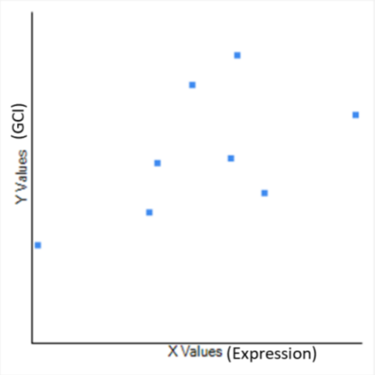

$X - M_x$	$Y - M_y$	$(X - M_x)^2$	$(Y - M_y)^2$	$(X - M_x)(Y - M_y)$
-2.611	-0.692	6.819	0.479	1.807
-0.851	-0.444	0.725	0.197	0.378
0.439	-0.038	0.193	0.001	-0.017
-0.721	-0.073	0.520	0.005	0.052
2.409	0.290	5.802	0.084	0.699
0.969	-0.298	0.938	0.089	-0.289
0.539	0.739	0.290	0.547	0.398
-0.171	0.515	0.029	0.266	-0.088
Mx: 4.451	My: 1.437	Sum: 15.316	Sum: 1.669	Sum: 2.941

Result Details & Calculation

X Values
∑ = 35.61
Mean = 4.451
$\sum(X - M_x)^2 = SS_x = 15.316$

Y Values
∑ = 11.496
Mean = 1.437
$\sum(Y - M_y)^2 = SS_y = 1.669$

X and Y Combined
N = 8
$\sum(X - M_x)(Y - M_y) = 2.941$

R Calculation
$r = \sum((X - M_y)(Y - M_x)) / \sqrt{((SS_x)(SS_y))}$

$r = 2.941 / \sqrt{((15.316)(1.669))} = 0.5818$

Meta Numerics (cross-check)
r = 0.5818

Key

X: X Values
Y: Y Values
M_x: Mean of X Values
M_y: Mean of Y Values
$X - M_x$ & $Y - M_y$: Deviation scores
$(X - M_x)^2$ & $(Y - M_y)^2$: Deviation Squared
$(X - M_x)(Y - M_y)$: Product of Deviation Scores

$$r = \frac{\sum_i (x_i - \overline{x})(y_i - \overline{y})}{\sqrt{\sum_i (x_i - \overline{x})^2}\sqrt{\sum_i (y_i - \overline{y})^2}}$$

The value of R is 0.5818.

This is a moderate positive correlation, which means there is a tendency for high X variable scores go with high Y variable scores (and vice versa).

*Mean of X (M_x) = $\bar{x}$, Mean of Y (M_y) = $\bar{y}$

f) Pearson's correlation coefficient (Expression *vs* GNDVI)

X Values

```
Geno : Expression
1-9  : 1.84
1-64 : 3.6
2-39 : 4.89
2-56 : 3.73
3-13 : 6.86
4-9  :  5.42
4-81 : 4.99
5-65 : 4.28
```

Y Values

```
(GNDVI)
]0.27142
0.3317
0.41163
0.40551
0.46341
0.39692
0.52111
0.49398
```

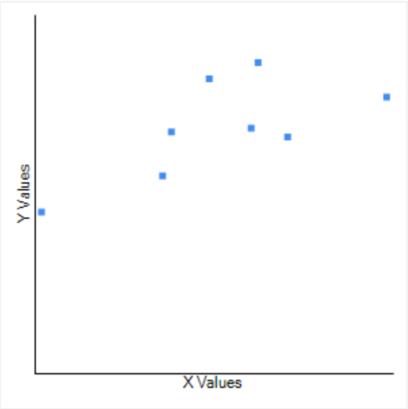

$X - M_x$	$Y - M_y$	$(X - M_x)^2$	$(Y - M_y)^2$	$(X - M_x)(Y - M_y)$
-2.611	-0.141	6.819	0.020	0.367
-0.851	-0.080	0.725	0.006	0.068
0.439	0.000	0.193	0.000	0.000
-0.721	-0.006	0.520	0.000	0.005
2.409	0.051	5.802	0.003	0.124
0.969	-0.015	0.938	0.000	-0.015
0.539	0.109	0.290	0.012	0.059
-0.171	0.082	0.029	0.007	-0.014
Mx: 4.451	My: 0.412	Sum: 15.316	Sum: 0.048	Sum: 0.594

Result Details & Calculation

X Values
$\sum = 35.61$
Mean = 4.451
$\sum(X - M_x)^2 = SS_x = 15.316$

Y Values
$\sum = 3.296$
Mean = 0.412
$\sum(Y - M_y)^2 = SS_y = 0.048$

X and Y Combined
$N = 8$
$\sum(X - M_x)(Y - M_y) = 0.594$

R Calculation
$r = \sum((X - M_y)(Y - M_x)) / \sqrt{((SS_x)(SS_y))}$

$r = 0.594 / \sqrt{((15.316)(0.048))} = 0.6945$

Meta Numerics (cross-check)
r = 0.6945

Key

X: X Values
Y: Y Values
M_x: Mean of X Values
M_y: Mean of Y Values
$X - M_x$ & $Y - M_y$: Deviation scores
$(X - M_x)^2$ & $(Y - M_y)^2$: Deviation Squared
$(X - M_x)(Y - M_y)$: Product of Deviation Scores

$$r = \frac{\sum_i (x_i - \bar{x})(y_i - \bar{y})}{\sqrt{\sum_i (x_i - \bar{x})^2}\sqrt{\sum_i (y_i - \bar{y})^2}}$$

The value of R is 0.6945.

This is a moderate positive correlation, which means there is a tendency for high X variable scores go with high Y variable scores (and vice versa).

*Mean of X (M_x) = $\bar{x}$, Mean of Y (M_y) = $\bar{y}$
