# Supplementary material for: Expression of Trichoderma spp. endochitinase gene improves red rot disease resistance in transgenic sugarcane
Source: PLoS One. 2024 Sep 16;19(9):e0310306. doi: 10.1371/journal.pone.0310306 (PMC11404804; doi:10.1371/journal.pone.0310306)
Supplement: S5 Table — (PDF) [file pone.0310306.s016.pdf]

S5 Table Bioassay of sugarcane for red rot disease incidence.

Plant no.	Condition on top[1]				Lesion width[2]				White spots[3]				Nodal transgression[4]				Scale (0-9)[5]				Category
**Inoculation using CF08 inoculum**																					
Plant no.	R1	R2	R3	S.E	R1	R2	R3	S.E	R1	R2	R3	S.E	R1	R2	R3	S.E	R1	R2	R3	S.E	
NTC	0	0	0	0±0	3	3	3	3±0	1	2	2	1.67±0.58	3	3	3	3±0	7.9	8	8	8 ± 0.06	S
Chit 1-9	0	0	0	0±0	3	3	3	3±0	1	1	1	1±0	1	2	2	1.67±0.58	4.1	4.1	4.4	4.2 ± 0.17	MS
Chit 1-64	0	0	0	0±0	3	3	3	3±0	1	1	1	1±0	1	2	2	1.67±0.58	4.1	4.1	4.2	4.1 ± 0.06	MS
Chit 2-39	0	0	0	0±0	2	2	3	2.33±0.58	0	1	1	0.67±0.58	1	2	2	1.67±0.58	3.7	3.2	4	3.6 ± 0.40	MR
Chit 2-56	0	0	0	0±0	3	3	3	3±0	1	1	1	1±0	2	2	2	2±0	4.1	4.2	5	4.4 ± 0.49	MS
Chit 3-13	0	0	0	0±0	2	2	2	2±0	0	0	0	0±0	1	1	2	1.33±0.58	2	2	2	2.0 ± 0.0	R
Chit 3-30	0	0	0	0±0	3	3	3	3±0	1	1	2	1.33±0.58	3	3	3	3±0	6.1	6.3	6.5	6.3 ± 0.2	S
Chit 3-45	0	0	0	0±0	3	3	3	3±0	1	1	1	1±0	2	2	2	2±0	5.1	4.1	5	4.7 ± 0.55	MS
Chit 4-9	0	0	0	0±0	2	3	3	2.67±0.58	0	0	1	0.33±0.58	1	1	2	1.33±0.58	2.4	3.2	3.1	2.9 ± 0.43	MR
Chit 4-81	0	0	0	0±0	2	2	3	2.33±0.58	0	0	0	0±0	1	2	2	1.67±0.58	3.1	3.2	3.6	3.3 ± 0.26	MR
Chit 5-65	0	0	0	0±0	2	3	3	2.67±0.58	0	0	1	0.33±0.58	1	1	2	1.33±0.58	2.4	3	3.4	2.9 ± 0.50	MR
**Inoculation using CF13 inoculum**																					
NTC	1	1	1	1±0	3	3	3	3±0	2	2	2	2±0	3	3	3	3±0	8.5	9.1	9.4	9 ± 0.46	HS
Chit 1-9	0	1	1	0.67±0.58	2	2	3	2.33±0.58	1	1	2	1.33±0.58	2	2	3	2.33±0.58	7.8	7.1	8	7.6 ± 0.47	S
Chit 1-64	1	1	1	1±0	3	3	3	3±0	2	2	2	2±0	3	3	3	3±0	8.5	8.8	8.5	8.6 ± 0.17	HS
Chit 2-39	1	1	1	1±0	3	3	3	3±0	2	2	2	2±0	3	3	3	3±0	8.1	8.2	8.6	8.3 ± 0.26	HS
Chit 2-56	0	1	1	0.67±0.58	2	2	3	2.33±0.58	1	1	2	1.33±0.58	2	2	3	2.33±0.58	8	7.1	8	7.7 ± 0.52	S
Chit 3-13	0	0	0	0±0	2	2	2	2±0	0	0	0	0±0	1	2	2	1.67±0.58	2	2	2	2.0 ± 0.0	R
Chit 3-30	1	1	1	1±0	2	3	3	2.67±0.58	1	1	2	1.33±0.58	2	3	3	2.67±0.58	8	8	8	8.0 ± 0.0	S
Chit 3-45	1	1	1	1±0	3	3	3	3±0	2	2	2	2±0	3	3	3	3±0	8.1	8.2	8.6	8.3 ± 0.26	HS
Chit 4-9	1	1	1	1±0	3	3	3	3±0	2	2	2	2±0	3	3	3	3±0	8.4	8.8	8.6	8.6 ± 0.2	HS
Chit 4-81	1	1	1	1±0	2	3	3	2.67±0.58	1	1	2	1.33±0.58	2	3	3	2.67±0.58	7.9	8	8	8.0 ± 0.06	S
Chit 5-65	1	1	1	1±0	3	3	3	3±0	2	2	2	2±0	3	3	3	3±0	8.4	8.1	8.4	8.3 ± 0.17	HS
