# Supplementary material for: Expression of Trichoderma spp. endochitinase gene improves red rot disease resistance in transgenic sugarcane
Source: PLoS One. 2024 Sep 16;19(9):e0310306. doi: 10.1371/journal.pone.0310306 (PMC11404804; doi:10.1371/journal.pone.0310306)
Supplement: S6 Table — (PDF) [file pone.0310306.s017.pdf]

S6 Table Association between gene expression and disease incidence in transgenic plants assessed using Pearson's correlation coefficient **a)** CF08. **b)** CF13.

**Plant designation**	**Relative *endochitinase* gene expression $2^{-\Delta\Delta C_T}$**	**Disease incidence**	
		**CF08**	**CF13**
NTC	1 (0.90-1.01)	8.0	9.0
Chit 1-9	1.83 (1.80-1.87)	4.2	7.6
Chit 1-64	3.60 (3.41-3.81)	4.1	8.6
Chit 2-39	4.82 (4.79-4.86)	3.6	8.3
Chit 2-56	3.68 (3.63-3.73)	4.4	7.7
Chit 3-13	6.87 (6.82-6.92)	2.0	2.0
Chit 3-30	0.93 (0.89-0.96)	5.2	8.0
Chit 3-45	1.12 (1.08-1.16)	4.7	8.3
Chit 4 -9	5.39 (5.24-5.54)	2.9	8.6
Chit 4-81	4.99 (4.89-5.09)	3.3	8.0
Chit 5-65	4.29 (4.14-4.44)	2.9	8.3

The association between fold-change in gene expression (x) and disease incidence (y) was assessed by Pearson's correlation coefficient using the formula:

$$r = \frac{\sum(x_i - \bar{x})(y_i - \bar{y})}{\sqrt{\sum(x_i - \bar{x})^2 \sum(y_i - \bar{y})^2}}$$

where, $r =$ Pearson's correlation coefficient; $x_i =$ values of x-variable in a sample; $\bar{x} =$ mean of the values of x-variable; $y_i =$ values of y-variable in a sample; $\bar{y} =$ mean of the values of y-variable.

a) CF08

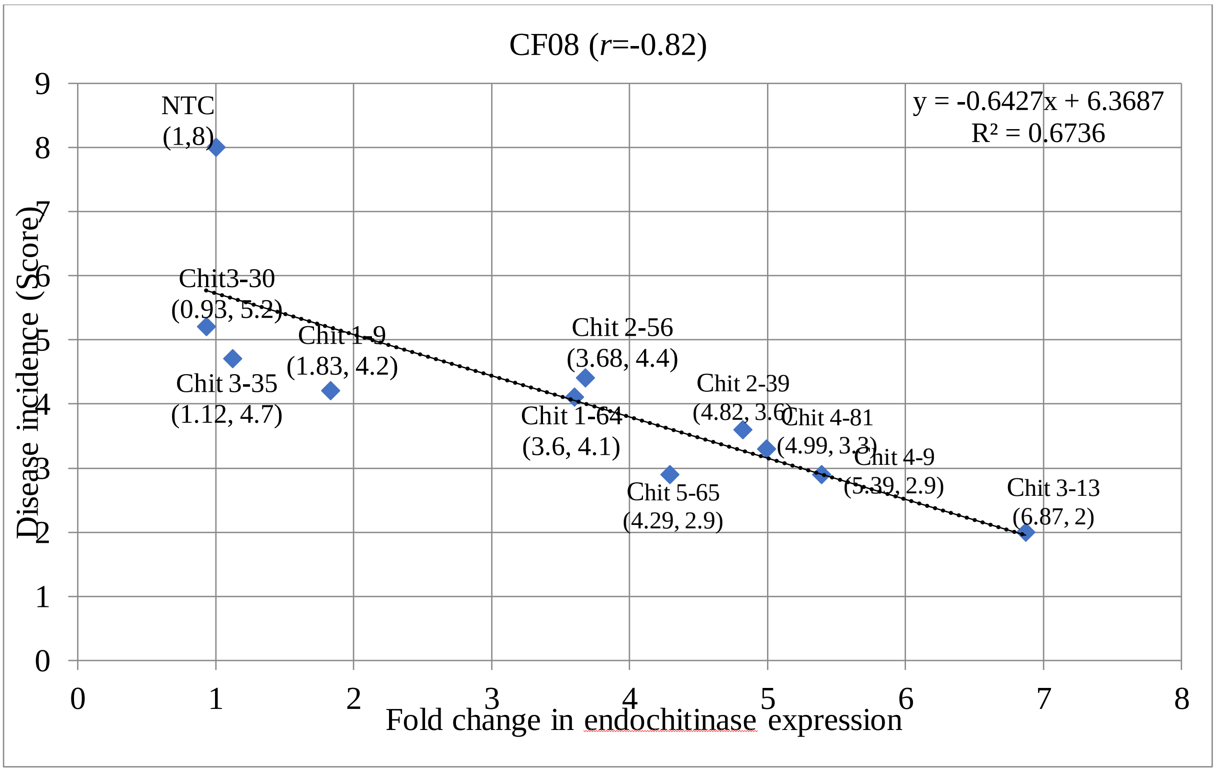

b) CF13

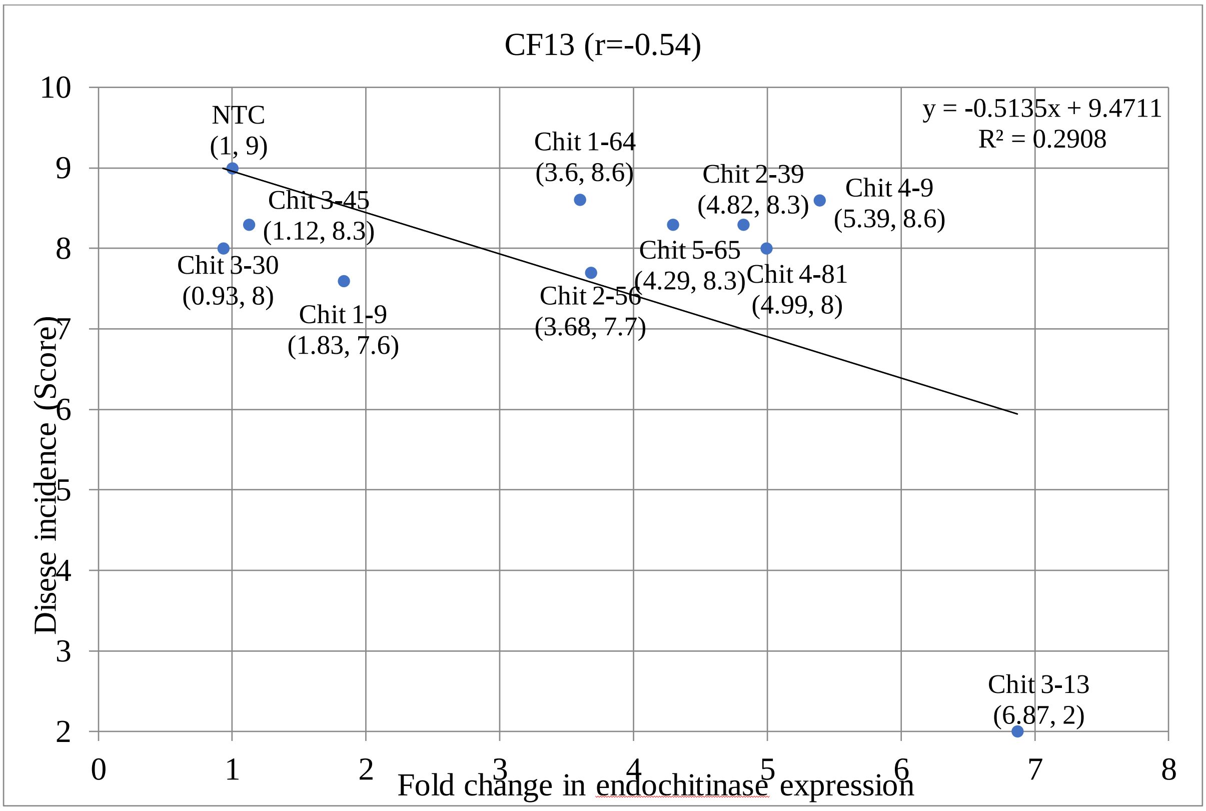

CF13 (r=-0.54)
y = -0.5135x + 9.4711
R² = 0.2908
NTC (1, 9)
Chit 3-45 (1.12, 8.3)
Chit 3-30 (0.93, 8)
Chit 1-9 (1.83, 7.6)
Chit 1-64 (3.6, 8.6)
Chit 2-56 (3.68, 7.7)
Chit 5-65 (4.29, 8.3)
Chit 2-39 (4.82, 8.3)
Chit 4-81 (4.99, 8)
Chit 4-9 (5.39, 8.6)
Chit 3-13 (6.87, 2)
Disese incidence (Score)
Fold change in endochitinase expression
