# Supplementary material for: Expression of Trichoderma spp. endochitinase gene improves red rot disease resistance in transgenic sugarcane
Source: PLoS One. 2024 Sep 16;19(9):e0310306. doi: 10.1371/journal.pone.0310306 (PMC11404804; doi:10.1371/journal.pone.0310306)
Supplement: S7 Table — (PDF) [file pone.0310306.s018.pdf]

**S7 Table** HR Brix in CF13-inoculated plants.

| Plant Designation | Brix (%) | | | | Sucrose losses (%) | | | |
|---|---|---|---|---|---|---|---|---|
| | **R1** | **R2** | **R3** | **Mean±SD** | **L1** | **L2** | **L3** | **Mean±SD** |
| NT (Non-inoculated) | 20.3 | 19.8 | 20.0 | 20.03±0.25 | 0 | 0 | 0 | NT |
| NT (Inoculated with CF13) | 10.5 | 11.5 | 11.0 | 11.0±0.50 | 48.27 | 41.92 | 45.00 | 45.06±3.17 |
| Chit1-9 | 14.8 | 15.2 | 15.0 | 15.0±0.20 | 27.09 | 23.23 | 25.00 | 25.11±1.93 |
| Chit 1-64 | 12.4 | 12.0 | 11.7 | 12.03±0.35 | 38.92 | 39.39 | 41.50 | 39.94±1.37 |
| Chit 2-39 | 12.5 | 13.7 | 12.4 | 12.86±0.72 | 38.42 | 30.81 | 38.00 | 35.74±4.28 |
| Chit 2-56 | 14.9 | 15.4 | 15.6 | 15.30±0.36 | 26.60 | 22.22 | 22.00 | 23.61±2.59 |
| Chit 3-13 | 17.6 | 18.2 | 17.8 | 17.86±0.30 | 13.30 | 8.08 | 11.00 | 10.79±2.62 |
| Chit 3-30 | 14.6 | 14.3 | 14.7 | 14.53±0.20 | 28.08 | 27.78 | 26.50 | 27.45±0.84 |
| Chit 3-45 | 12.7 | 13.2 | 12.9 | 12.93±0.25 | 37.44 | 33.33 | 35.50 | 35.42±2.06 |
| Chit 4-9 | 12.2 | 12.8 | 12.5 | 12.50±0.30 | 39.90 | 35.35 | 37.50 | 37.58±2.28 |
| Chit 4-81 | 14.6 | 14.8 | 14.7 | 14.70±0.10 | 28.08 | 25.25 | 26.50 | 26.61±1.42 |
| Chit 5-65 | 12.0 | 12.5 | 12.6 | 12.36±0.32 | 40.89 | 36.87 | 37.00 | 38.25±2.28 |

The sucrose losses (%) were calculated as:

$$\frac{\text{Brix value (\%) of NT non inoculated plant - Brix value (\%) of transgenic inoculated plant} \times 100}{\text{Brix value (\%) of NT non inoculated plant}}$$
